# Cellular connectomes as arbiters of local circuit models in the cerebral cortex

**DOI:** 10.1038/s41467-021-22856-z

**Published:** 2021-05-13

**Authors:** Emmanuel Klinger, Alessandro Motta, Carsten Marr, Fabian J. Theis, Moritz Helmstaedter

**Affiliations:** 1 Department of Connectomics, Max Planck Institute for Brain Research, Frankfurt, Germany; 2 Helmholtz Zentrum München, German Research Center for Environmental Health, Institute of Computational Biology, Neuherberg, Germany; 3 Technische Universität München, Center for Mathematics, Chair of Mathematical Modelling of Biological Systems, Garching, Germany

**Keywords:** Network models, Neural circuits

## Abstract

With the availability of cellular-resolution connectivity maps, connectomes, from the mammalian nervous system, it is in question how informative such massive connectomic data can be for the distinction of local circuit models in the mammalian cerebral cortex. Here, we investigated whether cellular-resolution connectomic data can in principle allow model discrimination for local circuit modules in layer 4 of mouse primary somatosensory cortex. We used approximate Bayesian model selection based on a set of simple connectome statistics to compute the posterior probability over proposed models given a to-be-measured connectome. We find that the distinction of the investigated local cortical models is faithfully possible based on purely structural connectomic data with an accuracy of more than 90%, and that such distinction is stable against substantial errors in the connectome measurement. Furthermore, mapping a fraction of only 10% of the local connectome is sufficient for connectome-based model distinction under realistic experimental constraints. Together, these results show for a concrete local circuit example that connectomic data allows model selection in the cerebral cortex and define the experimental strategy for obtaining such connectomic data.

## Abstract (web summary)

Large-scale connectomes from the mammalian brain are becoming available, but it remains unclear how informative these are for the distinction of circuit models. Here, the authors use connectome statistics to test competing models of local cortical circuits with approximate Bayesian computation.

## Introduction

In molecular biology, the use of structural (x-ray crystallographic or single-particle electron microscopic) data for the distinction between kinetic models of protein function constitutes the gold standard (e.g.,^1,2^). In Neuroscience, however, the question whether structural data of neuronal circuits is informative for computational interpretations is still heavily disputed^3–6^, with the extreme positions that cellular connectomic measurements are likely uninterpretable^6^ or indispensable^5^. In fact, structural circuit data has been decisive in resolving competing models for the computation of directional selectivity in the mouse retina^7^.

**Fig. 1:**
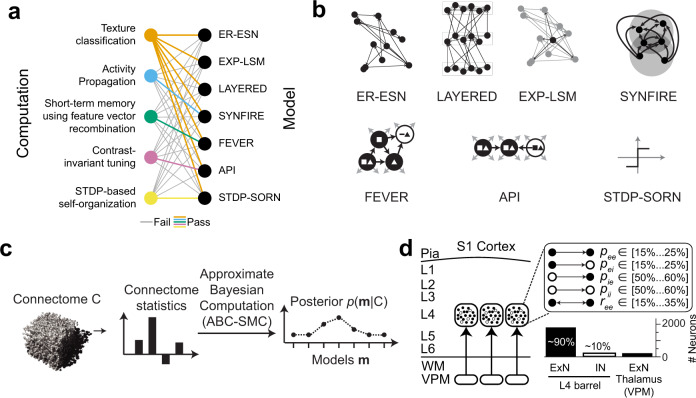
Relationship between models and possible computations in cortical circuits, and proposed strategy for connectomic model distinction in local circuit modules of the cerebral cortex. **a** Relationship between computations suggested for local cortical circuits (left) and possible circuit-level implementations (right). Colored lines indicate successful performance in the tested computation; gray lines indicate failure to perform the computation (see Supplementary Fig. 1 for details). **b** Enumeration of candidate models possibly implemented in a barrel-circuit module. See text for details. **c** Flowchart of connectomic model selection approach to obtain the posterior *p* (**m**|C) over hypothesized models **m** given a connectome C. ABC-SMC: approximate Bayesian computation using sequential Monte–Carlo sampling. **d** Sketch of mouse primary somatosensory cortex with presumed circuit modules (“barrels”) in cortical input layer 4 (L4). Currently known constraints of pairwise connectivity and cell prevalence of excitatory (ExN) and inhibitory (IN) neurons (${p}_{{ee}}$: pairwise excitatory-excitatory connectivity^30–33,36^, ${p}_{{ei}}$: pairwise excitatory-inhibitory connectivity^31,33^, ${p}_{{ii}}$: pairwise inhibitory-inhibitory connectivity^31,34^, ${p}_{{ie}}$: pairwise inhibitory-excitatory connectivity^31,33,35^, ${r}_{{ee}}$: pairwise excitatory-excitatory reciprocity^30,31,33^).

For the mammalian cerebral cortex, the situation can be considered more complicated: it can be argued that it is not even known which computation a given cortical area or local circuit module carries out. In this situation, hypotheses about the potentially relevant computations and about their concrete implementations are to be explored simultaneously. To complicate the investigation further, the relation between a given computation and its possible implementations is not unique. Take, for example pattern distinction (of tactile or visual inputs) as a possible computation in layer 4 of sensory cortex. This computation can be carried out by multi-layer perceptrons^8^, but also by random pools of connected neurons in an “echo state network”^9^ (Fig. 1a, Supplementary Fig. 1a–g) and similarly by networks configured as “synfire chains”^10^ (Fig. 1a). If one considers different computational tasks, however, such as the maintenance of sensory representations over time scales of seconds (short-term memory), or the stimulus tuning of sensory representations, then the relation between the computation and its implementation becomes more distinct (Fig. 1a). Specifically, a network implementation of antiphase inhibition for stimulus tuning^11^ is not capable of performing the short-term memory task (Supplementary Fig. 1k, l), and a network proposed for a short-term memory task (FEVER^12^), fails to perform stimulus tuning (Fig. 1a, Supplementary Figs. 1–3). Together, this illustrates that while it is impossible to uniquely equate computations with their possible circuit-level implementations, the ability to discriminate between proposed models would allow to narrow down the hypothesis space both about computations and their circuit-level implementations in the cortex.

With this background, the question whether purely structural connectomic data is sufficiently informative to discriminate between several possible previously proposed models and thus a range of possible cortical computations is of interest.

Here we asked whether for a concrete cortical circuit module, the “barrel” of a cortical column in mouse somatosensory cortex, the measurement of the local connectome can in principle serve as an arbiter for a set of possibly implemented local cortical models and their associated computations.

We developed and tested a model selection approach (using Approximate Bayesian Computation with Sequential Monte-Carlo Sampling, ABC-SMC^13–15^, Fig. 1c) on the main models proposed so far for local cortical circuits (Fig. 1b) ranging from pairwise random Erdős–Rényi (ER^16^) to highly structured “deep” layered networks used in machine learning^17,18^. We found that connectomic data alone is in principle sufficient for the discrimination between these investigated models, using a surprisingly simple set of connectome statistics. The model discrimination is stable against substantial measurement noise, and only partly mapped connectomes have already high discriminative power.

## Results

To develop our approach we focus on a cortical module in mouse somatosensory cortex, a “barrel” in layer 4 (L4), a main input layer to the sensory cortex^19–21^. The spatial extent of this module (roughly *d*_*b*_ = 300 μm along each dimension) makes it a realistic goal of experimentally mapped dense connectomes using state-of-the-art 3D electron microscopy^22,23^ and circuit reconstruction approaches^24–27^. A barrel is composed of about 2,000 neurons^28,29^. Of these about 90% are excitatory, and about 10% inhibitory^28,29^ (Fig. 1d), which establish a total of about 3 million chemical synapses within L4. The ensuing average pairwise synaptic connectivity within a barrel has been estimated based on data from paired whole-cell recordings^30–35^: excitatory neurons connect to about 15–25% of the other intra-barrel neurons; inhibitory neurons connect to about 50–60% of the other intra-barrel neurons (Fig. 1d). Moreover, the probability of a connection to be reciprocated ranges between 15% and 35%^29–31,33,36^. Whether intracortical connections in L4 follow only such pairwise connection statistics or establish higher-order circuit structure is not known^23,37–39^. Furthermore, it is not understood whether the effect of layer 4 circuits is primarily the amplification of incoming thalamocortical signals^30,40^, or whether proper intracortical computations commence within L4^41–43^. A L4 circuit module is therefore an appropriate target for model selection in local cortical circuits.

**Fig. 2:**
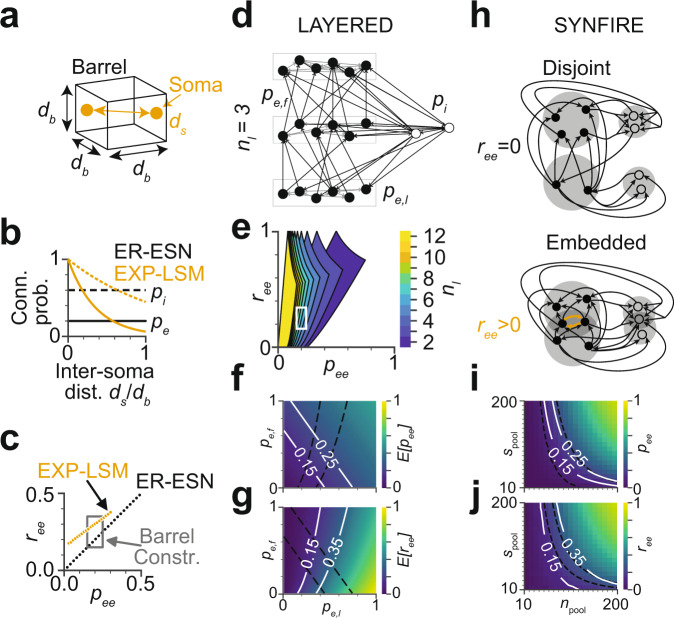
Compliance of candidate models with the so-far experimentally determined pairwise barrel circuit constraints in L4 (see Fig. 1d). **a** Illustration of a simplified cortical barrel of width ${d}_{b}$ and somata with inter soma distance ${d}_{s}$. **b** Pairwise excitatory and inhibitory connection probabilities ${p}_{e}$ and ${p}_{i}$ are constant over inter soma distance ${d}_{s}$ in the Erdős–Rényi echo state network (ER-ESN) and decay in the exponentially decaying connectivity - liquid state machine model (EXP-LSM). **c** Possible pairwise excitatory-excitatory connectivity ${p}_{{ee}}$ and excitatory-excitatory reciprocity ${r}_{{ee}}$ in the ER-ESN and EXP-LSM model satisfy the so-far determined barrel constraints (box). **d**–**g** Layered model: **d** example network with three layers (${n}_{l}=3$), excitatory forward (between-layer) connectivity ${p}_{e,f}$, excitatory lateral (within-layer) connectivity ${p}_{e,l}$ and inhibitory connectivity ${p}_{i}$. **e** Range of ${p}_{{ee}}$ and ${r}_{{ee}}$ in the LAYERED model for varying number of layers ${n}_{l}$ (white box: barrel constraints as in **c**). **f**, **g** Expected excitatory pairwise connectivity $E\left[{p}_{{ee}}\right]$ and reciprocity $E\left[{r}_{{ee}}\right]$ as function of ${p}_{e,l}$ and ${p}_{e,f}$ for ${n}_{l}=3$. Isolines indicate barrel constraints, model parameters in compliance with these constraints: area between intersecting isolines. Note that constraints are fulfilled only for within-layer connectivity ${p}_{e,l} \, > \, 0$, refuting a strictly feedforward network. **h**–**j** Embedded synfire chain model (SYNFIRE). **h** Two subsequent synfire pools in the disjoint (top) and embedded (bottom) synfire chain. Since intra-pool connectivity ${p}_{e,l}$ is strictly zero, reciprocal connections do not exist in the disjoint case (${r}_{{ee}}=0$) but in the embedded configuration. **i**, **j** Pairwise excitatory connectivity ${p}_{{ee}}$ and pairwise excitatory reciprocity ${r}_{{ee}}$ as function of the number of pools ${n}_{{{{\rm{pool}}}}}$ and the pool size ${s}_{{{{\rm{pool}}}}}$ for a SYNFIRE network with $N=2000$ neurons. Respective barrel constraints (white and dashed line). See Supplementary Fig. 2 for analogous analysis of FEVER, API, and STDP-SORN models.

The simplest model of local cortical circuits assumes pairwise random connectivity between neurons, independent of their relative spatial distance in the cortex (Erdős–Rényi^16^, Fig. 2a–c). This model has been proposed as Echo State Network (ESN^9,44^). As a slight modification, random networks with a pairwise connectivity dependent on the distance between the neurons’ cell bodies are the basis of liquid state machines (LSMs^45,46^, Fig. 2a–c). At the other extreme, highly structured layered networks are successfully used in machine learning and were originally inspired by neuronal architecture (multi-layer perceptrons^8^, Fig. 2d–g). Furthermore, embedded synfire chains have been studied (SYN^10,47^, Fig. 2h–j), which can be considered an intermediate between random and layered connectivity. In addition to these rather general model classes, particular suggestions of models for concrete cortical operations have been put forward that make less explicit structural assumptions (feature vector recombination network (FEVER^12^), proposed to achieve stimulus representation constancy on macroscopic timescales within a network; and antiphase inhibition (API^11,48^), proposed to achieve contrast invariant stimulus tuning), or that are based on local learning rules (spike timing-dependent plasticity/self-organizing recurrent neural network (STDP-SORN^49,50^)).

We first had to investigate whether the so far experimentally established circuit constraints of local cortical modules in S1 cortex (Fig. 1d; number of neurons, pairwise connectivity, and reciprocity; see above) were already sufficient to refute any of the proposed models.

Both the pairwise random ER model (Fig. 2c) and the pairwise random but soma-distance dependent EXP-LSM model are directly compatible with measured constraints on pairwise connectivity and reciprocity (Fig. 2c). A strictly layered multilayer perceptron model, however, does not contain any reciprocal connections and would in the strict form have to be refuted for cortical circuit modules, in which the reciprocity range is 0.15–0.35. Instead of rejecting such a “deep” layered model altogether, we studied a layered configuration of locally randomly connected ensembles (Fig. 2d). We found that models with up to ten layers are consistent with the circuit constraints of barrel cortex (Fig. 2e). In subsequent analyses we considered configurations with 2–4 layers. In this regime, the connectivity within layers is 0.2–0.6 and between layers 0.3–⁠0.6 (Fig. 2f, g; *n*_*l*_ = 3 layers). Similarly, disjoint synfire chains^10^ (Fig. 2h) would have to be rejected for the considered circuits due to lack of reciprocal connections. Embedded synfire chains (e.g., ref. ^47^), however, yield reciprocal connectivity for the sets of neurons overlapping between successive pools (Fig. 2h). This yields a range of pool sizes for which the SYNFIRE model is compatible with the known circuit constraints (Fig. 2i, j). The other models were investigated analogously (Supplementary Fig. 2), finding slight (API, Supplementary Fig. 2d–g) or substantial modifications (FEVER, STDP-SORN, Supplementary Fig. 2a–c, h–m) that make the models compatible with a local cortical circuit in L4. Notably, the FEVER model as originally proposed^12^ yields substantially too low connectivity and too high reciprocity to be realistic for local cortical circuits in L4 (Supplementary Fig. 2b). A modification in which FEVER rules are applied on a pre-drawn random connectivity rescues this model (Supplementary Fig. 2a, b).

### Structural model discrimination via connectome statistics

We then asked whether these local cortical models could be distinguished on purely structural grounds, given a binary connectome of a barrel circuit.

**Fig. 3:**
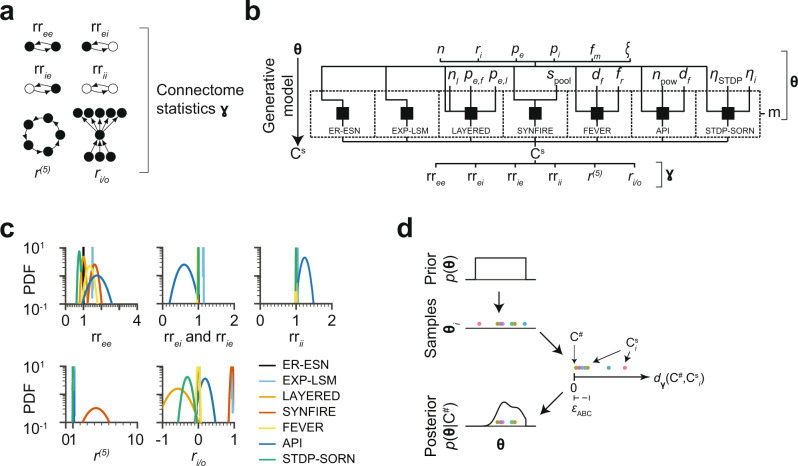
Connectome statistics and generative models for approximate Bayesian inference. **a** Connectome statistics ${{{\boldsymbol{\gamma }}}}$ used for model distinction: relative excitatory-excitatory reciprocity ${{{{\rm{rr}}}}}_{{ee}}$, relative excitatory-inhibitory reciprocity ${{{{\rm{rr}}}}}_{{ei}}$, relative inhibitory-excitatory reciprocity ${{{{\rm{rr}}}}}_{{ie}}$, relative inhibitory-inhibitory reciprocity ${{{{\rm{rr}}}}}_{{ii}}$, relative cycles of length 5, ${r}^{(5)}$, and in-out degree correlation of excitatory neurons ${r}_{i/o}.$ **b** Generative model for Bayesian inference: shared set of parameters (top: number of neurons $n$, fraction of inhibitory neurons ${r}_{i}$, excitatory connectivity ${p}_{e}$, inhibitory connectivity ${p}_{i}$, fractional connectome measurement ${f}_{m}$, noise $\xi$) and model-specific parameters (middle: model choice $m$, number of layers ${n}_{l}$, excitatory forward connectivity ${p}_{e,f}$, excitatory lateral connectivity ${p}_{e,l}$, pool size ${s}_{{{{\rm{pool}}}}}$, STDP learning rate ${\eta }_{{{{\rm{STDP}}}}}$, intrinsic learning rate ${\eta }_{i}$, feature space dimension ${d}_{f}$, feverization ratio ${f}_{r}$, selectivity ${n}_{{{{\rm{pow}}}}}$, see Supplementary Fig. 4), generated sampled connectome C^s^ described by the summary statistics ${{{\boldsymbol{\gamma }}}}=({{{{\rm{rr}}}}}_{{ee}},{{{{\rm{rr}}}}}_{{ei}},{{{{\rm{rr}}}}}_{{ie}},{{{{\rm{rr}}}}}_{{ii}},{r}^{\left(5\right)},{r}_{i/o})$. **c** Gaussian fits of probability density functions (PDFs) of the connectome statistics ${{{\boldsymbol{\gamma }}}}$ (**a**) for all models (see Fig. 1b). **d** Sketch of ABC-SMC procedure: given a measured connectome ${{{{\rm{C}}}}}^{{{{\rm{\#}}}}}$, parameters ${{{{\boldsymbol{\theta }}}}}_{i}$ (colored dots) are sampled from the prior $p({{{\boldsymbol{\theta }}}})$. Each ${{{{\boldsymbol{\theta }}}}}_{i}$ generates a connectome ${{{{\rm{C}}}}}_{i}^{{{{\rm{s}}}}}$ that has a certain distance ${d}_{{{{\boldsymbol{\gamma }}}}}\left({{{{\rm{C}}}}}^{{{{\rm{\#}}}}},{{{{\rm{C}}}}}_{i}^{{{{\rm{s}}}}}\right)$ to ${{{{\rm{C}}}}}^{{{{\rm{\#}}}}}$ in the space defined by the connectome statistics ${{{\boldsymbol{\gamma }}}}$ (**a**). If this distance is below a threshold ${\epsilon }_{{{{\rm{ABC}}}}}$, the associated parameters ${{{{\boldsymbol{\theta }}}}}_{i}$ are added as mass to the posterior distribution $p\left({{{\boldsymbol{\theta }}}}|{{{{\rm{C}}}}}^{{{{\rm{\#}}}}}\right)$, and are rejected otherwise.

We first identified circuit statistics ${{{\boldsymbol{\gamma }}}}$ that could serve as potentially distinctive connectome descriptors (Fig. 3a). We started with the relative reciprocity of connections within (${{{{\rm{rr}}}}}_{{ee}}$ and ${{{{\rm{rr}}}}}_{{ii}}$) and across (${{{{\rm{rr}}}}}_{{ei}}$ and ${{{{\rm{rr}}}}}_{{ie}}$) the populations of excitatory and inhibitory neurons. Since we had already found that some of the models would likely differ in reciprocity (see above, Fig. 2c, g, j Supplementary Fig. 2b, f, g), these statistics were attractive candidates. We further explored the network recurrency ${r}^{(l)}$ at cycle length $l$, which is a measure for the number of cycles in a network (Fig. 3a). This measure can be seen as describing how much of the information flow in the network is fed back to the network itself. So a LAYERED network would be expected to achieve a low score in this measure, while a highly recurrent network, such as SYNFIRE is expected to achieve a high score. We used ${r}^{(l)}$ with $l=5$ since for smaller $l$ this measure is more equivalent to the reciprocity ${r}_{{ee}}$ and for larger $l$, the measure is numerically less stable. Moreover, we investigated the in/out-degree correlation of the excitatory population ${r}_{i/o}$ (Fig. 3a). This measure was motivated by the notion that ${r}_{i/o} \, < \, 0$ should point towards a separation of input and output subpopulations of L4, as for example expected in the LAYERED model.

For a first assessment of the distinctive power of these six connectome statistics ${{{\boldsymbol{\gamma }}}}$, we sampled 50 L4 connectomes from each of the 7 models (Fig. 3b). The free parameters of the models were drawn from their respective prior distributions (Fig. 3b; priors shown in Supplementary Fig. 4). For example, for the LAYERED model, the prior parameters were the number of layers ${n}_{l}\in [2,4]$, the forward connectivity ${p}_{e,f}\in [0.19,0.57]$ and the lateral connectivity ${p}_{e,l}\in [0.26,0.43]$. The proposed network statistics ${{{\boldsymbol{\gamma }}}}$ (Fig. 3a) were then evaluated for each of the 350 sampled connectomes (Fig. 3b, c). While the statistics had some descriptive power for certain combinations of models (for example, ${{{\rm{r}}}}{{{{\rm{r}}}}}_{{ei}}$ seemed to separate API from EXP-LSM, Fig. 3c), none of the six statistics alone could discriminate between all the models (see the substantial overlap of their distributions, Fig. 3c), necessitating a more rigorous approach for model selection.

### Discrimination via Bayesian model selection

We used an Approximate Bayesian Computation-Sequential Monte Carlo (ABC-SMC) model selection scheme^13–15^ to compute the posterior probability over a range of models given a to-be-measured connectome ${{{{\rm{C}}}}}^{\#}$.

In this approach, example connectomes ${{{{\rm{C}}}}}^{{{{\rm{s}}}}}$ are generated from the models **m** in question (using the priors over the model parameters ${{{\boldsymbol{\theta }}}}$ (Fig. 3b, d; see Supplementary Fig. 4 for plots of all priors)). For each sampled connectome ${{{{\rm{C}}}}}^{{{{\rm{s}}}}}$, the dissimilarity ${d}_{{{{\boldsymbol{\gamma }}}}}({{{{\rm{C}}}}}^{{{{\rm{s}}}}},{{{{\rm{C}}}}}^{\#} )$ to the measured connectome ${{{{\rm{C}}}}}^{\#}$ was computed (formalized as a distance ${d}_{{{{\boldsymbol{\gamma }}}}}({{{{\rm{C}}}}}^{{{{\rm{s}}}}},{{{{\rm{C}}}}}^{\#} )$ between ${{{{\rm{C}}}}}^{{{{\rm{s}}}}}$ and ${{{{\rm{C}}}}}^{\#}$). The connectome distance was defined as an L1 norm over the six connectome statistics ${{{\boldsymbol{\gamma }}}}$ (Fig. 3a), normalized by the 20%-to-80% percentile per connectome statistic (see Methods). If the sampled connectome ${{{\rm{C}}}}^{{{\rm{s}}}}$ was sufficiently similar to the measured connectome ${{{\rm{C}}}}^{\#}$ (i.e. their distance ${d}_{{{{\boldsymbol{\gamma }}}}} ({{{\rm{C}}}}^{{{\rm{s}}}}, {{{\rm{C}}}}^{\#} )$ was below a preset threshold ${\epsilon }_{{{{\rm{ABC}}}}}$, see Methods), the sample was accepted and considered as evidence towards the model that had generated ${{{\rm{C}}}}^{{{\rm{s}}}}$ (Fig. 3d). With this, an approximate sample from the posterior $p ({{{\boldsymbol{\theta }}}} | {{{\rm{C}}}}^{\#} )$ was obtained (Fig. 3d). The posterior $p({{{\boldsymbol{\theta }}}}{{{\rm{|}}}}{{{{\rm{C}}}}}^{\#} )$ was iteratively refined by resampling and perturbing the parameters of the accepted connectomes and by sequentially reducing the distance threshold ${\epsilon }_{{{{\rm{ABC}}}}}$.

We then tested our approach on simulated connectomes ${{{{\rm{C}}}}}^{\#}$. These were again generated from the different model classes (as in Fig. 3b); however in the ABC method, only the distances ${d}_{{{{\boldsymbol{\gamma }}}}}({{{{\rm{C}}}}}^{{{{\rm{s}}}}},{{{{\rm{C}}}}}^{\#} )$ between the sampled connectomes ${{{{\rm{C}}}}}^{{{{\rm{s}}}}}$ and the simulated connectomes ${{{{\rm{C}}}}}^{\#}$ were used (Fig. 3d). It was therefore not clear a-priori whether the statistics ${{{\boldsymbol{\gamma }}}}$ are sufficiently descriptive to distinguish between the models; and whether this would be the case for all or only some of the models.

**Fig. 4:**
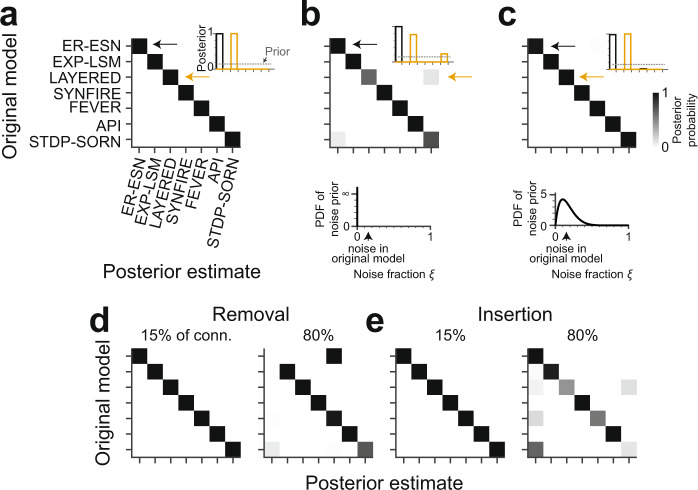
Identification of models using Bayesian model selection under ideal and noisy connectome measurements. **a** Confusion matrix reporting the posteriors over models given example connectomes. Example connectomes were sampled from each model class (rows; Fig. 3b) and then exposed to the ABC-SMC method (Fig. 3d) using only the connectome statistics (Fig. 3a). Note that all model classes are uniquely identified from the connectomes (inset: average posteriors for ER-ESN and LAYERED connectomes, respectively; *n* = 3 repetitions). **b** Posteriors over models given example connectomes to which a random noise of 15% (inset, dashed line) was added before applying the ABC-SMC method. The generative model (Fig. 3b) was ignorant of this noise (*n* = 3 repetitions; bottom: noise prior $p\left(\xi \right)={{{{\rm{\delta }}}}}_{\xi ,0}$). **c** Same analysis as in **b**, this time including a noise prior into the generative model (*n* = 3 repetitions). Bottom: The noise prior was modeled as $p\left(\xi \right)={{{\rm{Beta}}}}({{{\mathrm{2,10}}}})$. Note that in most connectome measurements, the level of reconstruction errors is quantifiable, such that the noise can be rather faithfully incorporated into the noise prior (see text). Model identification is again accurate under these conditions (compare **c** and **a**). **d** Confusion matrix when simulating split errors in neuron reconstructions by randomly removing 15% (left) or 80% (right) of connections before ABC-SMC inference. **e** Confusion matrix when simulating merge errors in neuron reconstructions by insertion of additional 15% (left) and 80% (right) of the original number of connections into random locations in the connectome before ABC-SMC inference. **d**, **e** Noise prior during ABC-SMC inference was of the same type as the simulated reconstruction errors (*n* = 1 repetition; noise prior $p\left(\xi \right)={{{\rm{Beta}}}}({{{\mathrm{2,10}}}})$). Color bar in **c** applies to all panels.

We first considered the hypothetical case of a dense, error-free connectomic reconstruction of a barrel circuit under the ER-ESN model yielding a connectome ${{{{\rm{C}}}}}^{\#}$. The ABC-SMC scheme correctly identified this model as the one model class at which the posterior probability mass was fully concentrated compared to all other models (Fig. 4a). ABC-SMC inference was repeated for *n* = 3 ER-ESN models, resulting in three consistent posterior distributions. Similarly, connectomes ${{{{\rm{C}}}}}^{\#}$ obtained from all other investigated models yielded posterior probability distributions concentrated at the correct originating model (Fig. 4a). Thus, the six connectome statistics ${{{\boldsymbol{\gamma}}}}$ together with ABC-based model selection were in fact able to distinguish between the tested set of models given binary connectomes.

### Discrimination of noisy connectomes

We next explored the stability of our approach in the face of connectome measurements in which ${{{{\rm{C}}}}}^{\#}$ was simulated to contain noise from biological sources, or errors resulting from connectomic reconstruction inaccuracies. The latter would be caused by the remaining errors made when reconstructing neuronal wires in dense nerve tissue^24,26,27,51^ and by remaining errors in synapse detection, especially when using automated synapse classifiers^52–57^. To emulate such connectome noise, we first randomly removed 15% of the connections in ${{{{\rm{C}}}}}^{\#}$ and reinserted them again randomly. We then computed the posterior on such noisy connectomes ${{{{\rm{C}}}}}^{\#}$, which in fact became less stable (Fig. 4b; shown is average of *n* = 3 repetitions with accuracies of 83.0%, 99.8%, and 100.0%, respectively).

However, in this setting, we were pretending to be ignorant about the fact that the connectome measurement was noisy (see noise prior in Fig. 4b), and had assumed a noise-free measurement. In realistic settings, however, the rate of certain reconstruction errors can be quantitatively estimated. For example, the usage of automated synapse detection^57^ and neurite reconstructions with quantified error rates^24,26,27,58–60^, provide such error rates explicitly. We therefore next investigated whether prior knowledge about the reconstruction error rates would improve the model posterior (Fig. 4c). For this, we changed our prior assumption about reconstruction errors $\xi$ from noise-free (Fig. 4b) to a distribution with substantial probability mass around 0–30% noise (modeled as $p(\xi )\sim {{{\rm{Beta}}}}(2,10)$, Fig. 4c). When we applied the posterior computation again to connectomes ${{{{\rm{C}}}}}^{\#}$ with 15% reconstruction noise, these were now as discriminative as in the noise-free case (Fig. 4c, cf. Fig. 4a, b).

To further investigate the effect of biased noise, we also tested conditions in which synaptic connections were only randomly removed or only randomly added (corresponding to cases in which reconstruction of the connectome may be biased towards neurite splits (Fig. 4d) or neurite mergers (Fig. 4e)); and cases in which errors were focused on a part of the connectome (corresponding to cases in which certain neuronal connections may be more difficult to reconstruct than others, Supplementary Fig. 5a). These experiments indicate a rather stable range of faithful model selection under various types of measurement errors.

### Incomplete connectome measurement

**Fig. 5:**
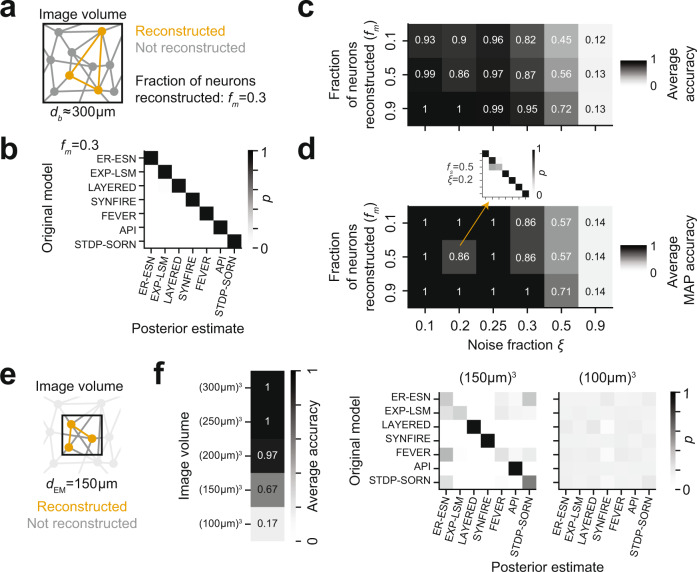
Model selection for partially measured and noisy connectomes. **a** Fractional (incomplete) connectome measurement when reconstructing only a fraction *f*_*m*_ of the neurons in a given circuit, thus obtaining a fraction *f*_*m*_^*2*^ of the complete connectome. **b** Effect of incomplete connectome measurement on model selection performance for *f*_*m*_ = 0.3 (no noise; *n* = 1 repetition). Note that model selection is still faithfully possible. **c**, **d** Combined effects of noisy and incomplete connectome measurements on model selection accuracy reported as average posterior probability (**c**; *n* = 1 repetition per entry) and maximum-a-posteriori accuracy (**d**; *n* = 1 repetition per entry). Note that model selection is highly accurate down to 10% fractional connectome measurement at up to 25% noise, providing an experimental design for model distinction that is realistic under current connectome measurement techniques (see text). Model selection used a fixed $p\left(\xi \right)={{{\rm{Beta}}}}({{{\mathrm{2,10}}}})$ noise prior. More informative noise priors result in more accurate model selection (Supplementary Fig. 5b). **e** Effect of fractional dense circuit reconstruction: Locally dense connectomic reconstruction of the neurons and of their connections in a circuit subvolume. **f** Effect of partial imaging and dense reconstruction of the circuit subvolume on average model selection accuracy (left: *n* = 1 repetition per entry). Note that model selection based on dense reconstruction of a (150 μm)^3^ volume (12.5% of circuit volume) is substantially less accurate than model selection based on complete reconstructions of 10% in the complete circuit volume (see **c**). Right: Posterior distributions over models for image volumes of (150 μm)^3^ and (100 μm)^3^, respectively (*n* = 1 repetition, each).

In addition to reconstruction noise, a second serious practical limitation of connectomic measurements is the high resource consumption (quantified in human work hours, which are in the range of 90,000–180,000 h for a full barrel reconstruction today, assuming 1.5 mm/h reconstruction speed, 5–10 km path length per cubic millimeter and a barrel volume of (300 µm)^3^ ^24,61^). Evidently, the mapping of connectomes for model discrimination would be rendered substantially more feasible if the measurement of only a fraction of the connectome was already sufficient for model discrimination. We therefore next investigated the stability of our discrimination method under two types of fractional measurements (Fig. 5).

We first tested whether reconstruction of only *f*_*m*_ = 30% of neurons and of their connectivity is sufficient for model selection (Fig. 5a). We found model discrimination to be 100% accurate in the absence of reconstruction errors (Fig. 5b). This reconstruction assumes the 3D EM imaging of a tissue volume that comprises an entire barrel, followed by a fractional circuit reconstruction (see sketch in Fig. 5a). Such an approach is realistic since the speed of 3D EM imaging has increased more quickly than that of connectomic reconstruction^61–64^.

We then screened our approach for stability against both measurement noise and incomplete connectome measurement by applying our method on connectomes of varying noise rates $\xi$ and measurement fractions *f*_*m*_ with a fixed noise prior ($p(\xi )\sim {{{\rm{Beta}}}}(2,10)$). For evaluating classification performance, we used two approaches: first, we averaged the model posterior along the diagonal of the classification matrix (e.g., Fig. 5b), yielding the average accuracy for a given noise and fractional measurement combination (Fig. 5c). In addition, we evaluated the quality of the maximum-a-posteriori (MAP) classification, which takes the peak of the posterior as binary classification result (Fig. 5d). The MAP connectome classification was highly accurate even in a setting in which only 10% of the connectome were sampled, and at a substantial level of reconstruction error of 25%. This implies that we will be able to perform the presented model distinction in a partially mapped barrel connectome consuming 18,000 instead of 180,000 work hours^24,57,61^ (Fig. 5c, d). Evidently, this makes a rather unrealistic reconstruction feasible (note the largest reconstructions to date consumed 14,000–25,000 human work hours^58–60,65^).

We then asked whether complete connectomic reconstructions of small EM image volumes^27^ could serve as an alternative to the fractional reconstruction of large image volumes (Fig. 5e, f). This would reduce image acquisition effort and thereby make it realistic to rapidly compare how brain regions, species or disease states differ in terms of circuit models. To simulate locally dense reconstructions, we first restricted the complete noise-free connectome to the neurons with their soma located within the imaged barrel subvolume (Fig. 5e). Importantly, connections between the remaining neurons may be established outside the image volume. To account for the loss of these connections, we further subsampled the remaining connections. We found model selection from dense connectomic reconstruction of a (150 µm)^3^ volume (12.5% of the barrel volume) to be unstable (67% average accuracy; Fig. 5f) due to the confusion between the ER-ESN, EXP-LSM, FEVER, and STDP-SORN models (Fig. 5f). For the dense reconstruction of (100 µm)^3^, accuracy of model selection was close to chance level for all models (17% average accuracy; Fig. 5f). So our tests indicate that an experimental approach in which the image volume comprises an entire local cortical circuit module (barrel), but the reconstruction is carried out only in a subset of about 10–15% of neurons is favored over a dense reconstruction of only 12.5% of the barrel volume. Since the imaging of increasingly larger volumes in 3D EM from the mammalian brain is becoming feasible^64,66^, while its reconstruction is still a major burden, these results propose a realistic experimental setting for connectomic model selection in the cortex.

### Incomplete set of hypotheses

**Fig. 6:**
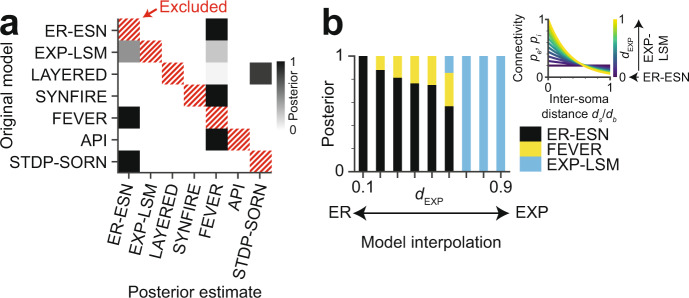
Effect of incomplete hypothesis space and of model interpolation on Bayesian model selection. **a** Confusion matrix reporting the posterior distribution when excluding the true model (hatched) from the set of tested model hypotheses (*n* = 1 repetition). Note that posterior probability is non-uniformly distributed and concentrated at plausibly similar models even when the true model is not part of the hypothesis space. **b** Posterior distributions for connectome models interpolated between ER-ESN and EXP-LSM (*n* = 1 repetition per bar). Inset: Space constant *d*_EXP_ acts as interpolation parameter between ER-ESN (*d*_EXP_ = 0) and EXP-LSM (*d*_EXP_ = 1). Note that the transition between the two models is captured by the estimated model posterior, with an intermediate (non-dominant) confusion with the FEVER model.

Bayesian analyses can only compare evidence for hypotheses known to the researcher. But what if the true model is missing from the set of tested hypotheses? To investigate this question, we excluded the original model during inference of the posterior distribution from a complete noise-free barrel connectome (Fig. 6a). In these settings, rather than obtaining uniformly distributed posteriors, we found that the probability mass of the posterior distributions was concentrated at one or two of the other models. The FEVER model, for example, which is derived from pairwise random connectivity (ER-ESN) while imposing additional local constraints that result in heightened relative excitatory-excitatory reciprocity, resembles the EXP-LSM model (see Fig. 3c). Accordingly, these three models (ER-ESN, EXP-LSM, FEVER) showed a high affinity for mutual confusion when the original model was excluded during ABC-SMC (Fig. 6a). This may indicate that our Bayesian model selection approach assigns the posterior probability mass to the most similar tested models, thus providing a ranking of the hypotheses. Notably, models with zero posterior probability in the confusion experiment (Fig. 6a) were in fact almost exclusively those at largest distance from the original model. As a consequence, rejecting the models with zero posterior probability mass may provide falsification power even when the “true” model is not among the hypotheses.

In order to investigate whether our approach provided sensible model interpolation in cases of mixed or weak model evidence (Fig. 6b), we considered the following example. The EXP-LSM model turns into an ER-ESN model in the limit of large decay constants $\lambda$ of pairwise connectivity (that is modeled to depend on inter-soma distance, see inset Fig. 6b). This allowed us to test our approach on connectomes that were sampled from models interpolated between these two model classes. When we exposed such “mixed” connectomes to our model discrimination approach, the resulting posterior had most of its mass at the EXP-LSM model for samples with ${d}_{{{{\rm{EXP}}}}}$ close to 1 and much of its mass at the ER-ESN model for samples with ${d}_{{{{\rm{EXP}}}}}$ close to 0. For intermediate model mixtures, the Bayesian model selection approach in fact yielded interpolated posterior probability distributions. This result gave an indication that the approach had in fact some stability against model mixing.

### Connectomic separability of sparse recurrent neural networks trained on different tasks

**Fig. 7:**
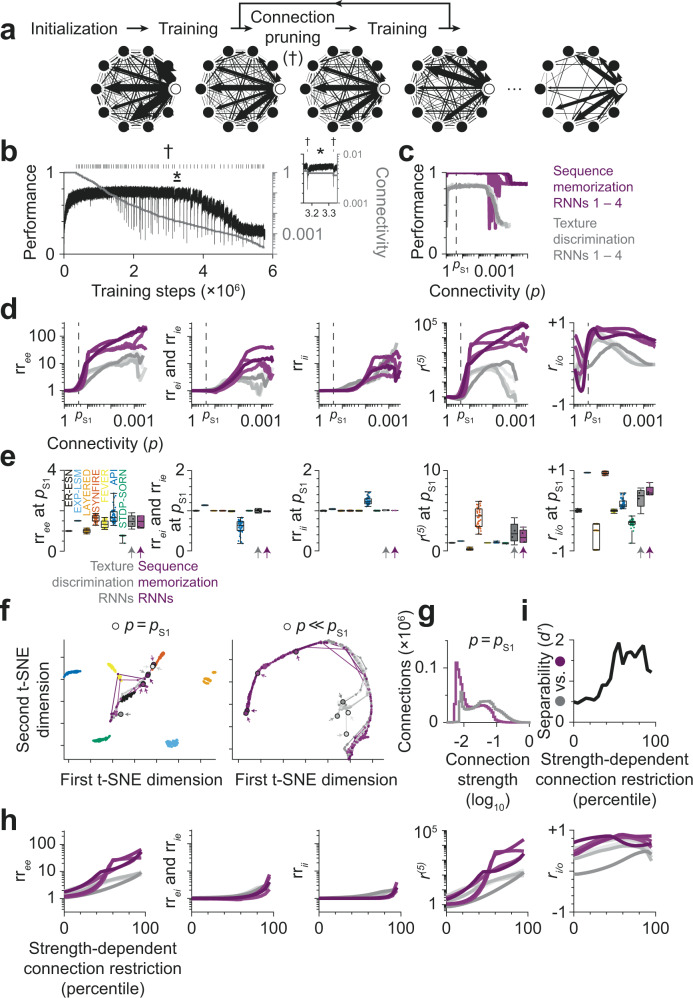
Connectomic separability of recurrent neural network (RNNs) with similar initialization, but trained on different tasks. **a** Overview of training process: RNNs were initially fully connected. Whenever task performance saturated during training, the weakest 10% of connections were pruned (†) to obtain a realistic level of sparsity. **b** Task performance (black) and network connectivity (gray) of a texture discrimination RNN during training. Ticks indicate the pruning of connections. Inset (*): Connection pruning causes a decrease in task performance, which is (partially) compensated by further training of the remaining connections. **c** Task performance as a function of network connectivity (*p*). Performance defined as: Accuracy (Texture discrimination RNNs, gray); 1 – mean squared error (Sequence memorization RNNs, magenta). Note that maximum observed performance was achieved in a wide connectivity regime including connectivity consistent with experimental data (*p*_S1_ = 24%; dashed line). Task performance started to decay after pruning at least 99.6% of connections. **d** Connectome statistics of RNNs over iterative training and pruning of connections (cf. Fig. 3a). **e** Distribution of connectome statistics at *p* = *p*_S1_ for RNNs and structural network models. Note that structural network models and structurally unconstrained RNNs exhibit comparable variance in connectome statistics (rr_*ee*_: 0.088 vs. 0.15 for API; rr_*ei*_ and rr_*ie*_: 0.0019 vs. 0.026 for API; rr_*ii*_: 9.35 × 10^−7^ vs. 8.17 × 10^−3^ for API; *r*^*(5)*^: 1.54 vs. 1.51 for SYNFIRE; r_*i/o*_: 0.057 vs. 0.061 for LAYERED; cf. Fig. 3c). RNNs trained on different tasks did not differ significantly in terms of connectome statistics (rr_*ee*_: 1.48 ± 0.30 vs. 1.46 ± 0.29, *p* = 0.997; rr_*ei*_ and rr_*ie*_: 1.00 ± 0.04 vs. 0.99 ± 0.01, *p* = 0.534; rr_*ii*_: 1.01 ± 0.01 vs. 1.01 ± 0.00, *p* = 0.107; *r*^*(5)*^: 2.28 ± 1.24 vs. 1.84 ± 0.80, *p* = 0.997; *r*_*i/o*_: 0.31 ± 0.24 vs. 0.49 ± 0.12, *p* = 0.534; mean ± std for *n* = 4 texture discrimination vs. sequence memorization RNNs, each; two-sided Kolmogorov-Smirnov test without correction for multiple comparisons). Boxes: center line is median; box limits are quartiles; whiskers are minimum and maximum; all data points shown. **f** Similarity of RNNs based on connectome statistics (lines) as connectivity approaches biologically plausible connectivity p_S1_ (circles and arrows, left) and for connectivity range from 100% to 0.04% (circles and arrows, right). Note that connectome statistics at ≤11% connectivity separate texture discrimination and sequence memorization RNNs into two clusters. **g** Distribution of connection strengths at *p* = *p*_S1_ for two RNNs trained on different tasks. **h** Connectome statistics of RNNs with *p*_S1_ connectivity when ignoring weak connections. **i** Separability of texture discrimination and sequence memorization RNNs with biologically plausible connectivity based on statistics derived from weighted connectome.

Finally, we asked whether recurrent neural networks (RNNs) that were randomly initialized and then trained on different tasks could be distinguished by the proposed model selection procedure based on their connectomes after training. To address this question, we trained RNNs on either a texture discrimination task or a sequence memorization task. Initially, all RNNs were fully connected with random connection strengths (Fig. 7a). During training, connection strengths were modified by error back-propagation to maximize performance on the task. At the same time, we needed to reduce the connectivity *p* of the RNNs to a realistic level of sparsity (*p*_S1_$\in$[0.15…0.25], see Fig. 1d) and used the following strategy: Whenever task performance saturated, we interrupted the training to identify the weakest 10% of connections and permanently pruned them from the RNN (Fig. 7b). This training-pruning cycle then continued on the remaining connections. As a result, connectivity within an RNN was constrained only by the task used for training.

Maximum task performance was reached early in training while connectivity was still high (*p* ≈ 80%) and started to decay only after pruning more than 99.6% of connections (*p* < 0.4%). Within this connectivity range (80% ≥ *p* ≥ 0.4%), task performance substantially exceeded chance level (approx. 82.8–83.8% vs. 14.3% accuracy for *n* = 4 texture discrimination RNNs; 0.000–0.002 vs. 0.125 mean squared error for *n* = 4 sequence memorization RNNs; range of measurements vs. chance level; Fig. 7c). Importantly, task performance was at the highest achieved level also at realistic connectivity of *p*_S1_ = 24%.

We then investigated the connectome statistics applied to the RNNs during training (Fig. 7d). We wanted to address the following two questions: First, how strongly are connectome statistics constrained by the training task? In particular, is the variance of connectome statistics in trained RNNs much larger than in network models that are primarily defined by their structure (e.g., LAYERED or SYNFIRE)? Second, does training of RNNs on different tasks result in different connectomic structures? And if so, are the connectome statistics sensitive enough to distinguish RNNs trained on different tasks based only on their structure?

At 24% connectivity, we found the variance of the connectome statistics to be comparable to the variance in structural network models (Fig. 7e; cf. Figure 3c), but connectome statistics of RNNs trained on different tasks were statistically indistinguishable (Fig. 7e), and RNNs with different tasks were thus only poorly separable (sensitivity index *d’* of 0.495; Fig. 7f). However, we noticed a separation into two clusters when RNNs were trained and further sparsified to a connectivity of *p* << 11% (*d’* = 1.45 ± 0.23, mean ± std; Fig. 7f).

To further study the effect of sparsification of a trained RNN, we investigated whether additional information about the strength of connections (Fig. 7g) could improve the separability of RNNs trained on different tasks. We started with the weighted connectomes of RNNs that were trained and sparsified to 24% connectivity. For the evaluation of connectome statistics, we then restricted the RNNs to strong connections (Fig. 7h). When ignoring the weakest 50% of connections of each RNN, the texture discrimination and sequence memorization RNNs differed significantly in their relative excitatory→excitatory reciprocity (3.60 ± 0.99 vs. 7.83 ± 1.98, *p* = 0.011) and relative prevalence of cycles (22.19 ± 12.69 vs. 118.16 ± 40.86, *p* = 0.011; Fig. 7h). As a result, RNNs trained on different tasks could be separated by the six connectome statistics with 85 ± 3% accuracy (Fig. 7i, separability *d’* = 1.61 ± 0.24, mean ± std). We concluded that RNNs with biologically plausible connectivity that were trained on different tasks could be distinguished based on the proposed statistics derived from weighted connectomes, in which only the strongest connections were used for connectome analysis.

## Discussion

We report a probabilistic method to use a connectome measurement as evidence for the discrimination of local models in the cerebral cortex. We show that the approach is robust to experimental errors, and that a partial reconstruction of the connectome suffices for model distinction. We furthermore demonstrate the applicability to large cortical connectomes consisting of thousands of neurons. Surprisingly, a set of rather simple connectome statistics is sufficient for the discrimination of a large range of models. These results show that and how connectomes can function as arbiters of local cortical models^5^ in the cerebral cortex.

Previous work on the classification of connectomes addressed smaller networks, consisting of up to 100 neurons, in which the identity of each neuron was explicitly defined. For these settings, the graph matching problem was approximately solved^67^. However, such approaches are currently computationally infeasible for larger unlabeled networks^67,68^, which are found in the cerebral cortex.

As an alternative, the occurrence of local circuit motifs has been used for the analysis of local neuronal networks^69–71^. Four of our connectome statistics (Fig. 3a) could be interpreted as such motifs: the relative reciprocity within and across the excitatory and inhibitory neuron populations, whose prevalence we could calculate exactly. The key challenge of these descriptive approaches is the interpretation of the observed motifs. The Bayesian approach as proposed here provides a way to use such data as relative, discriminating evidence for possible underlying circuit models.

One approach for the analysis of neuronal connectivity data is the extraction of descriptive graph properties (for example those termed clustering coefficient^72^, small-worldness^73^, closeness- and betweenness centrality^74^), followed by a functional interpretation of these measures. Such discovery-based approaches have been successfully applied especially for the analysis of macroscopic whole-brain connectivity data^75,76^.

The relationship between (static) network architecture and task performance was previously studied in feed-forward models of primate visual object recognition^77,78^, in which networks with higher object recognition performance were shown to yield better prediction of neuronal responses to visual stimuli. Our study considered recurrent neural networks, accounting for the substantial reciprocity in cortical connectivity, and investigated the structure-function relationship for static recurrent network architectures on a texture classification task (Supplementary Fig. 1), as well as for sparse recurrent neural networks in which both network architecture and task performance were jointly optimized (Fig. 7).

Pre-hoc connectome analyses, in which the circuit models are defined before connectome reconstruction, offer several advantages over exploratory analyses, where the underlying circuit model is constructed after-the-fact: First, the statistical power of a test with pre-hoc defined endpoints is substantially higher^79,80^, rendering pre-hoc endpoint definition a standard for example in the design of clinical studies^79^. Especially since so far, microscopic dense connectomes are mostly obtained and interpreted form a single sample, *n* = 1^23,58,81,82^, this concern is substantial, and a pre-hoc defined analysis relieves some of this statistical burden. Moreover, the pre-hoc analysis allowed us to determine an experimental design for the to-be-measured connectome, defining bounds on reconstruction and synapse errors and the required connectome measurement density (Fig. 5c, d). Especially given the substantial challenge of data analysis in connectomics^61^, this is a relevant practical advantage.

We considered it rather unexpected that a 10% fractional reconstruction, and reconstruction errors up to 25% would be tolerable for the selection of local circuit models. One possible reason for this is the homogeneity of the investigated network models. For each model, the (explicit or implicit) structural connectivity rules are not defined per neuron individually, but apply to a whole sub-population of neurons. For example, the ER-ESN model implies one connectivity rule for all excitatory neurons and a second one for all inhibitory neurons; the layered model defines one connectivity rule for each layer. Hence, the model properties were based on the wiring statistics of larger populations, permitting low fractional reconstruction and substantial wiring errors. If, on the contrary, the network models were to define for each neuron a very specific connectivity structure, a different experimental design would likely be favorable, in which the precise reconstruction of few individual neurons could suffice to refute hypotheses.

How critical were the particular circuit constraints which we considered for initial model validation (Fig. 1d)? What if, for example, pairwise excitatory connectivity was lower than concluded from pairwise recordings in slice (Fig. 1d^28–36^), and instead for example rather 10%, not 15–25% in L4? The results on discriminability of trained RNNs (Fig. 7), which was higher for sparser networks, may indicate that model identification would even improve for lower overall connectivity regimes. Also, such a setting would imply that the model priors would be in a different range (Supplementary Fig. 4; for example the layered network with four layers would imply a pairwise forward connectivity ${p}_{e,f}$ = 27% instead of 53%). Circuit measurements that already clearly refute any of the hypothesized models based on simple pairwise connectivity descriptors would of course reduce the model space a-priori. Once a full connectomic measurement is available, the connectivity constraints (Fig. 1d) can be updated, the model hypothesis space diminished or not, and then our model selection approach can be applied.

The choice of summary statistics in ABC is generally not unique, and poorly chosen statistics may bias model selection^83–85^. Our use of emulated reconstruction experiments with known originating models was therefore required to verify ABC performance (Figs. 4–6). These results also indicate that it was sufficient to use summary statistics that were constrained to operate on unweighted graphs. More detailed summary statistics that also make use of indicators of synaptic weights accessible in 3D EM data (such as size of post-synaptic density, axon-spine interface, or spine head volume^86–88^) may allow further distinction of plasticity models with subtle differences in neuronal activity history^27^. In fact, we found that weighted connectomes were necessary to distinguish between circuit models that were subject to identical structural constraints and that only differed in the tasks that they performed (Fig. 7).

The proposed Bayesian model selection also has a number of drawbacks.

First, likelihood-free model inference using ABC-SMC depends on efficient simulation of the models. Computationally expensive models, such as recurrent neural networks trained by stochastic gradient descent (Fig. 7), are prohibitive for sequential Monte Carlo sampling. However, the proposed connectome statistics and the resulting connectomic distance function provide a quantitative measure of similarity even for individual samples (Fig. 7i). Furthermore, a rough estimate of the posterior distribution over models can be obtained already by a single round of ABC-SMC with a small sample size.

Second, an exhaustive enumeration of all hypotheses is needed for Bayesian model selection. What if none of the investigated models was correct? This problem cannot be escaped in principle, and it has been argued that Bayesian approaches have the advantage of explicitly and transparently accounting for this lack of prior knowledge rather than implicitly ignoring it^89^. Nevertheless, this caveat strongly emphasizes the need for a proper choice of investigated models. Our results (Fig. 6) indicate that models close to but not identical to any of the investigated ones are still captured in the posterior by reporting their relative similarity to the remaining investigated models. We argue that rejection of models without posterior probability mass provides valuable scientific insights, even when the set of tested hypotheses is incomplete.

Third, we assumed a flat prior over the investigated models, considering each model equally likely a-priori. Pre-conceptions about cortical processing could strongly alter this prior model belief. If one assumed a non-homogenous model prior, this different prior can be multiplied to the posterior computed in our approach. Therefore, the computed posterior can in turn be interpreted as a quantification of how much more likely a given model would have to be considered by prior belief in order to become the classification result, enabling a quantitative assessment of a-priori model belief about local cortical models.

Together, we show that connectomic measurement carries substantial distinctive power for the discrimination of models in local circuit modules of the cerebral cortex. The concrete experimental design for the identification of the most likely local model in cortical layer 4, proposed pre-hoc, will make the mapping of this cortical connectome informative and efficient. Our methods are more generally applicable for connectomic comparison of possible models of the nervous system.

## Methods

### Circuit constraints

The following circuit constraints were shared across all cortical network models. A single barrel was assumed to consist of 1800 excitatory and 200 inhibitory neurons^28,29^. The excitatory connectivity ${p}_{e}$, i.e. the probability of an excitatory neuron to project to any other neuron was assumed to be ${p}_{{{{\rm{ee}}}}}={p}_{{{{\rm{ei}}}}}=0.2$^30–33,36^, the excitatory-excitatory reciprocity ${r}_{{ee}}$, i.e., the probability of also observing a bidirectional connection given one connection between two excitatory neurons, was assumed to lie in the range ${r}_{{ee}}\in [0.15,0.35]$^29–31,33,36^. The inhibitory connectivity ${p}_{i}$, i.e., the probability of an inhibitory neuron to project onto any other neuron, was assumed as ${p}_{{ii}}={p}_{{ie}}=0.6$^31,33–35^. Self-connections were not allowed.

### Estimates of reconstruction time and synapse number

Neurite path length density was assumed to be $d=10{{{\rm{km}}}}/{{{{\rm{mm}}}}}^{3}$, barrel volume was assumed to be *V* = (300 µm)^3^, annotation speed was taken as $v=1.5{{{\rm{mm}}}}/{{{\rm{h}}}}$^24^ together yielding the total annotation time $T={Vd}/v$.

The total number of synapses in a barrel was calculated as ${Nf}=3,2299,091$ with $f=3.36$ the average number of synapses per connection^30^ and $N=2000\cdot (1800\cdot 0.2+200\cdot 0.6)$ the total number of synaptically connected pairs of neurons.

### Implementations of cortical network models

Seven cortical models were implemented: the Erdős–Rényi echo state network (ER-ESN^9,16^), the exponentially decaying connectivity - liquid state machine model (EXP-LSM^45,46^), the layered model (LAYERED^8,90^), the synfire chain model (SYNFIRE^10,11,48^), the feature vector recombination model (FEVER^12^), the antiphase inhibition model (API) and the spike timing-dependent plasticity self-organizing recurrent neural network model (STDP-SORN^49,50^).

The Erdős–Rényi echo state network (ER-ESN) model was a directed Erdős–Rényi random graph. Each possible excitatory projection was realized with probability ${p}_{e}=0.2$, each possible inhibitory projection with probability ${p}_{i}=0.6$.

For the exponentially decaying connectivity - liquid state machine model (EXP-LSM), excitatory and inhibitory neurons were assumed to be uniformly and independently distributed in a cubic volume of equal side lengths. The excitatory and inhibitory pairwise connection probabilities ${p}_{e}(d)$ and ${p}_{i}(d)$ were functions of the Euclidean distance $d$ of a neuron pair according to ${p}_{t}(d)={p}_{0}{{{\rm{exp }}}}\left(\frac{-d}{{\lambda }_{t}}\right)$, ${p}_{0}={p}_{t}+\left(1-{p}_{t}\right){d}_{{{{\rm{EXP}}}}}$, ${d}_{{{{\rm{EXP}}}}}=1$, $t\in \left\{{{{\rm{e}}}},{{{\rm{i}}}}\right\}$. The length scale parameters ${\lambda }_{t}$ were adjusted to match an overall connectivity of ${p}_{e}=0.2$ in the excitatory case ($t=e$) and a connectivity of ${p}_{i}=0.6$ in the inhibitory case ($t=i$).

The layered model (LAYERED) consisted of ${n}_{l}$ excitatory layers. Lateral excitatory-excitatory connections were realized within one layer with connection probability ${p}_{e,l}$. Forward connections from one layer to the next layer were realized with probability ${p}_{e,f}$. Inhibitory neurons were not organized in layers but received excitatory projections uniformly and independently from all excitatory neurons with probability ${p}_{e}=0.2$ and projected onto any other neuron uniformly and independently with probability ${p}_{i}=0.6$.

The synfire chain (SYNFIRE) implementation used in this work followed^47^. The inhibitory pool size ${s}_{{{{\rm{pool}}}},i}=\frac{{n}_{i}}{{n}_{e}}{s}_{{{{\rm{pool}}}}}$ was proportional to the excitatory pool size ${s}_{{{{\rm{pool}}}}}$. The network was constructed as follows: (1) An initial excitatory source pool of size ${s}_{{{{\rm{pool}}}}}$ was chosen uniformly from the excitatory population. (2) An excitatory target pool of size ${s}_{{{{\rm{pool}}}}}$ and an inhibitory target pool of size ${s}_{{{{\rm{pool}}}},i}$ were chosen uniformly. The excitatory source and target pools were allowed to share neurons, i.e., neurons were drawn with replacement. (3) The excitatory source pool was connected all-to-all to the excitatory and inhibitory target pools but no self-connections were allowed. (4) The excitatory target pool was chosen to be the excitatory source pool for the next iteration. Steps (2) to (4) were repeated ${{{\rm{round}}}}\left(\frac{{{{\rm{log }}}}\left(1-{p}_{e}\right)}{{{{\rm{log }}}}\left(1-\frac{{s}_{{{{\rm{pool}}}}}^{2}}{{n}_{e}^{2}}\right)}\right)$ times, with ${{{\rm{round}}}}(\bullet )$ denoting the nearest integer. Inhibitory neurons projected uniformly to any other neuron with probability ${p}_{{{{\rm{i}}}}}=0.6$.

The feature vector recombination model (FEVER) network was constructed from an initial ER random graph ${{{{\rm{C}}}}}^{0}$ with initial pairwise connection probabilities ${p}_{t}^{0}={p}_{t}-{f}_{r}{d}_{f}/n$ for $t\in \left\{e,i\right\}$ with ${f}_{r}\in [0,1]$ the feverization, ${d}_{f}{\mathbb{\in }}{\mathbb{N}}$ the feature space dimension and $n$ the number of neurons. The outgoing projections ${{{{\bf{c}}}}}_{k}$ of neuron $k$ were obtained from ${{{{\rm{C}}}}}^{0}$ according to the sparse optimization problem ${{{{\bf{c}}}}}_{k}={{{{\rm{argmin}}}}}_{{{{\bf{c}}}}}\left\{{\sum }_{l\ne k}{\left\Vert {{{{\bf{d}}}}}_{l}-{\sum }_{p\ne k}{{{{\bf{d}}}}}_{p}{{{{\rm{c}}}}}_{p}\right\Vert }_{2}^{2}+{\lambda }_{t(k)}{\left\Vert {{{{\bf{c}}}}}_{k}^{0}-{{{\bf{c}}}}\right\Vert }_{1}\right\}$, $,{c}_{{kk}}=0,$ where the ${{{{\bf{d}}}}}_{i}\in {{\mathbb{R}}}^{{d}_{f}}$ were the feature vectors drawn uniformly and independently from a unit sphere of feature space dimension ${d}_{f}$ and ${{{{\bf{c}}}}}_{k}^{0}{{\mathbb{\in }}{\mathbb{R}}}^{n}$ denoted the initial outgoing projections of neuron $k$ as given by ${{{{\rm{C}}}}}^{0}$ and $t\left(k\right)=e$ if neuron $k$ was excitatory, $t\left(k\right)=i$ otherwise. The sparse optimization was performed with scikit-learn^91^ using the “sklearn.linear_model.Lasso” optimizer with the options “positive = True” and “max_iter = 100000” for the excitatory and the inhibitory population individually. The parameter ${\lambda }_{t}$, $t\in \left\{e,i\right\}$ was fitted to match the excitatory and inhibitory connectivity of ${p}_{e}=0.2$ and ${p}_{i}=0.6$ respectively.

In the antiphase inhibition model (API), a feature vector ${{{{\bf{d}}}}}_{k}$ was associated with each neuron $k$. The feature vectors were drawn uniformly and independently from a unit sphere with feature space dimension ${d}_{f}$. The cosine similarity ${C}_{{ij}}={c}_{{{{\rm{sim}}}}}({{{{\bf{d}}}}}_{i},{{{{\bf{d}}}}}_{j})$ between the feature vectors of neuron $i$ and $j$ were transformed into connection probabilities ${p}_{{ij}}$ between neuron $i$ and $j$ according to ${p}_{{ij}}=1-{\left({1-\left(\frac{{C}_{{ij}}{s}_{j}+1}{2}\right)}^{{n}_{{{{\rm{pow}}}}}}\right)}^{{n}_{{s}_{j}}^{{{{\rm{binomial}}}}}}$, where ${s}_{j}=1$ if neuron $j$ was excitatory and ${s}_{j}=-1$ if neuron $j$ was inhibitory. The coefficients ${n}_{x}^{{{{\rm{binomial}}}}}$ with $x\in \{-{{{\mathrm{1,1}}}}\}$ were fitted to match the excitatory and inhibitory connectivity constraints. The coefficient ${n}_{{{\rm{pow}}}}$ was in the range ${n}_{{{\rm{pow}}}}\in [{{{\mathrm{4,6}}}}]$ (Supplementary Fig. 4f^11^).

The spike timing dependent plasticity self-organizing recurrent neural network model (STDP-SORN) network was constructed as follows: An initial random matrix ${{{{\rm{C}}}}}_{0}\in {\{{0,1},-1\}}^{n \times n}$ with pairwise connection probabilities ${p}_{t}$ for $t\in \left\{e,i\right\}$ was drawn. Let ${s}_{e,k}={\sum }_{{{{\rm{l:}}}}{{{{\rm{C}}}}}_{{kl}} > 0}{{{{\rm{C}}}}}_{{kl}}$ denote the sum of all excitatory incoming weights of neuron $k$ and similarly ${s}_{i,k}=-{\sum }_{{l:}{{{{\rm{C}}}}}_{{kl}} < 0}{{{{\rm{C}}}}}_{{kl}}$ denote the sum of all inhibitory incoming weights of neuron $k$. Each weight ${{{{\rm{C}}}}}_{{kl}}\, > \, 0$ was normalized according to ${{{{\rm{C}}}}}_{{kl}}\leftarrow {{{{\rm{C}}}}}_{{kl}}/{s}_{e,k}$ and each weight ${{{{\rm{C}}}}}_{{kl}} \, < \, 0$ according to ${{{{\rm{C}}}}}_{{kl}}\leftarrow {{{{\rm{C}}}}}_{{kl}}/{s}_{i,k}$ such that for each neuron the sum of all incoming excitatory weights was $1$ and the sum of all incoming inhibitory weights was $-1$. No self-connections were allowed. The so obtained matrix was the initial adjacency matrix ${{{\rm{C}}}}$. The initial vector of firing thresholds ${{{\bf{t}}}}\in {{\mathbb{R}}}^{n}$ was initialized to ${{{\bf{t}}}}={{{\boldsymbol{1}}}}$. The neuron state ${{{\bf{x}}}}\in {\left\{{0,1}\right\}}^{n}$ and the past neuron state ${{{{\bf{x}}}}}_{{{{\rm{old}}}}}\in {\left\{{0,1}\right\}}^{n}$ were initialized as zero vectors.

After initialization, for each of the ${\tau }_{{{{\rm{end}}}}}={{{\mathrm{10,000}}}}$ simulation time points, the following steps were repeated^50^: (1) Propagation, (2) Intrinsic plasticity, (3) Normalization, (4) STDP, (5) Pruning and (6) Structural plasticity as follows:

Propagation. The neuron state ${{{\bf{x}}}}\in {\left\{{0,1}\right\}}^{n}$ was updated ${{{\bf{x}}}}\leftarrow \varTheta ({{{\rm{C}}}}{{{\bf{x}}}}{{{\boldsymbol{+}}}}{{{\boldsymbol{\xi }}}}{{{\boldsymbol{-}}}}{{{\bf{t}}}})$, where ${{{\boldsymbol{\xi }}}}$ was noise with ${{{{\boldsymbol{\xi }}}}}_{k}\sim {{{\rm{N}}}}(0,{\sigma }^{2})$ iid., $\sigma =0.05$ and $$\varTheta \left(x\right)=\left\{\begin{array}{c}1,{x}\ge 0\\ 0, {{{\rm{otherwise}}}}\end{array}\right.$$.

Intrinsic plasticity. The firing thresholds were updated ${{{\bf{t}}}}\leftarrow {{{\bf{t}}}}+{\eta }_{i}({{{\bf{x}}}}-{f}_{0})$ where ${f}_{0}=1/10$ was the target firing rate and ${\eta }_{i}$ the intrinsic plasticity learning rate.

Normalization. The excitatory incoming weights were normalized to $1$: If ${{{{\rm{C}}}}}_{{kl}} \, > \, 0$ then ${{{{\rm{C}}}}}_{{kl}}\leftarrow {{{{\rm{C}}}}}_{{kl}}/{s}_{e,k}.$

STDP (Spike timing dependent plasticity). Weights were updated according to ${{{{\rm{C}}}}}_{{kl}}\leftarrow {{{{\rm{C}}}}}_{{kl}}+{\eta }_{{{{\rm{STDP}}}}}\left({{{{\bf{x}}}}}_{k}{{{{\bf{x}}}}}_{{{{\rm{old}}}},l}+{{{{\bf{x}}}}}_{k}{{{{\bf{x}}}}}_{l}-{{{{\bf{x}}}}}_{{{{\rm{old}}}},k}{{{{\bf{x}}}}}_{l}\right)$ for $k\,\ne\, l$. Finally the past neuron state was also updated ${{{{\bf{x}}}}}_{{{{\rm{old}}}}}\leftarrow {{{\bf{x}}}}.$

Pruning. Weak synapses were removed: If ${0\,\le\, {{{\rm{C}}}}}_{{kl}} \, < \, 1/n$ then ${{{{\rm{C}}}}}_{{kl}}\leftarrow 0$.

Structural plasticity. It was attempted to add ${n}_{{{{\rm{add}}}}}=({n}_{e}^{2}{p}_{e}-{n}_{s})/(1-{p}_{e})$ synapses randomly, with ${n}_{s}={\sum }_{k,{l:}{{{{\rm{C}}}}}_{{kl}} > 0}1$ the number of excitatory synapses currently present in the network. For each of these attempts two integers $k,l\sim {{{\rm{DiscreteUniform}}}}(0,{n}_{e})$ were chosen randomly and independently. If $k\,\ne\, l$ and ${{{{\rm{C}}}}}_{{kl}}=0$ then ${{{{\rm{C}}}}}_{{kl}}\leftarrow 1/n$.

The STDP-SORN model was implemented in Cython and OpenMP.

All code was verified using a set of unit tests with 91% code coverage.

### Reconstruction errors and network subsampling

Reconstruction errors were implemented by randomly rewiring connections: A fraction $\xi$ of the edges of the network was randomly removed, ignoring their signs. The same number of edges was then randomly reinserted and the signs were adjusted to match the sign of the new presynaptic neuron. Partial connectomic reconstruction was implemented by network subsampling: A fraction ${f}_{m}\in [{0,1}]$ of the neurons was uniformly drawn. The subgraph induced by these neurons was preserved, its complement discarded.

### Connectomic cortical network measures

The following measures (Fig. 3a) were computed: (1) relative excitatory-excitatory reciprocity, (2) relative excitatory-inhibitory reciprocity, (3) relative inhibitory-excitatory reciprocity, (4) relative inhibitory-inhibitory reciprocity, (5) relative excitatory recurrency, and (6) excitatory in/out-degree correlation. All measures were calculated on binarized networks as follows:

Reciprocity ${r}_{{xy}}$ with $x,y\in \left\{e,i\right\}$, *e* = excitatory, *i* = inhibitory, was defined as the number of reciprocally connected neuron pairs between neurons of population $x$ and $y$ divided by the total number of directed connections from $x$ to $y$. If the number of connections from $x$ to $y$ was zero then ${r}_{{xy}}$ was set to zero. Hence ${r}_{{xy}}$ was an estimate for the conditional probability of observing the reciprocated edge of a connection from $y$ to $x$, given a connection from $x$ to $y$. The relative excitatory-inhibitory reciprocity was defined as ${{{{\rm{rr}}}}}_{{ei}}={r}_{{ei}}/{p}_{{ie}}$. I.e., relative reciprocities were obtained by dividing the reciprocity of a network by the expected reciprocity of an ER network with the same connectivity.

Relative excitatory recurrency was defined as ${r}^{(n)}={{{\rm{tr}}}}\left({{{{\rm{C}}}}}_{{ee}}^{n}\right)/{\left({n}_{e}{p}_{e}\right)}^{n}$, where ${{{{\rm{C}}}}}_{{ee}}$ was the excitatory submatrix and ${{{\rm{tr}}}}$ denoted the trace of the matrix. The cycle length parameter $n$ was set to $n=5$.

The excitatory in/out-degree correlation ${r}_{i/o}$ was the Pearson correlation coefficient of the in- and out-degrees of neurons of the excitatory subpopulation. Let ${d}_{i,k}$ denote the in-degree of neuron $k$ and ${d}_{o,k}$ the out-degree of neuron $k$. Let $\bar{{d}_{i}}=\frac{1}{{n}_{e}}{\sum }_{k=1}^{{n}_{e}}{d}_{i,k}$ and $\overline{{d}_{o}}=\frac{1}{{n}_{e}}{\sum }_{k=1}^{{n}_{e}}{d}_{o,k}$, with ${n}_{e}$ the total number of excitatory neurons. Then ${r}_{i/o}=\frac{{\sum }_{k=1}^{{n}_{e}}({d}_{i,k}-\overline{{d}_{i}})({d}_{o,k}-\overline{{d}_{o}})}{\sqrt{{\sum }_{k=1}^{{n}_{e}}{\left({d}_{i,k}-\overline{{d}_{i}}\right)}^{2}}\sqrt{{\sum }_{k=1}^{{n}_{e}}{\left({d}_{o,k}-\overline{{d}_{o}}\right)}^{2}}}$.

### Bayesian model selection

Bayesian model selection was performed on networks sampled from the seven models as follows: First, a noise-free network ${{{{\rm{C}}}}}_{0}$ with 2000 neurons was drawn from one of the network models $m\in [1,\ldots ,7]$. Second, this noise-free network was perturbed with noise of strength $\xi$ as described above. Then, a fraction ${f}_{m}$ of the network was subsampled, yielding ${{{{\rm{C}}}}}^{{{{\rm{\#}}}}}$.

The Bayesian posterior $p({{{\boldsymbol{\theta }}}}|{{{{\rm{C}}}}}^{{{{\rm{\#}}}}})$ was then calculated on the noisy subnetwork ${{{{\rm{C}}}}}^{{{{\rm{\#}}}}}$ using an approximate Bayesian-sequential Monte Carlo (ABC-SMC) method. The implemented ABC-SMC algorithm followed the ABC-SMC procedure proposed by^92^ with slight modifications to ensure termination of the algorithm, as described below. The ABC-SMC algorithm was implemented as custom Python library (see Supplementary Code file and https://gitlab.mpcdf.mpg.de/connectomics/discriminatEM).

The network measures ${{{\boldsymbol{\gamma }}}}=({{{{\rm{rr}}}}}_{{ee}},{{{{\rm{rr}}}}}_{{ei}},{{{{\rm{rr}}}}}_{{ie}},{{{{\rm{rr}}}}}_{{ii}},{r}^{\left(5\right)},{r}_{i/o})$ described above were used as summary statistics for the ABC-SMC algorithm. The distance between two networks ${{{{\rm{C}}}}}^{\#}$ and ${{{{\rm{C}}}}}_{i}^{{{{\rm{s}}}}}$ was defined as ${d}_{{{{\boldsymbol{\gamma }}}}}\left({{{{\rm{C}}}}}^{{{{\rm{\#}}}}},{{{{\rm{C}}}}}_{i}^{{{{\rm{s}}}}}\right)=\mathop{\sum }\nolimits_{k=1}^{6}\frac{\left|{{{{\boldsymbol{\gamma }}}}}_{k}\left({{{{\rm{C}}}}}^{{{{\rm{\#}}}}}\right)-{{{{\boldsymbol{\gamma }}}}}_{k}\left({{{{\rm{C}}}}}_{i}^{{{{\rm{s}}}}}\right)\right|}{{{{{\boldsymbol{\gamma }}}}}_{k,80}-{{{{\boldsymbol{\gamma }}}}}_{k,20}}$, where the sum over $k$ was taken over the six network measures. The quantities ${{{{\boldsymbol{\gamma }}}}}_{k,80}$ and ${{{{\boldsymbol{\gamma }}}}}_{k,20}$ were the 80% and 20% percentiles of the measure ${{{{\boldsymbol{\gamma }}}}}_{k}$, evaluated on an initial sample from the prior distribution of size 2000; the particle number, i.e., the number of samples per generation, was set to 2000. If a particle of the initial sample contained an undefined measure (e.g., in-/out-degree correlation), it was discarded. When ${{{{\boldsymbol{\gamma }}}}}_{k,80}$ and ${{{{\boldsymbol{\gamma }}}}}_{k,20}$ were equal, the corresponding normalization constant of the distance function was set to the machine epsilon instead. The initial acceptance distance ${\epsilon }_{{{{\rm{ABC}}}}}$ was the median of the distances ${d}_{{{{\boldsymbol{\gamma }}}}}\left({{{{\rm{C}}}}}^{\#} ,\,{{{{\rm{C}}}}}_{i}^{{{{\rm{s}}}}}\right)$ as obtained from the same initially sampled connectomes ${{{{\rm{C}}}}}_{i}^{{{{\rm{s}}}}}$.

After each generation, ${\epsilon }_{{{{\rm{ABC}}}}}$ for the following generation was set to the median of the error distances ${d}_{{{{\boldsymbol{\gamma }}}}}\left({{{{\rm{C}}}}}^{\#} ,{{{{\rm{C}}}}}_{i}^{{{{\rm{s}}}}}\right)$ of the particles in the current generation. Particles were perturbed hierarchically. First, a model $m$ was drawn from the current approximating posterior model distribution. With probability 0.85 the model $m$ was kept, with probability 0.15 it was redrawn uniformly from all models. Second, given the sampled model, a single particle from the model specific particles was sampled. The sampled particle was perturbed according to a multivariate normal kernel with twice the variance of the variance of the particles in the current population of the given model. The perturbed particle was accepted if the error distance was below ${\epsilon }_{{{{\rm{ABC}}}}}$. To obtain again 2000 particles for the next population, 2000 particle perturbation tasks were run in parallel. However, to ensure termination of the algorithm, each of the 2000 tasks was allowed to terminate without returning a new particle if more than 2000 perturbation attempts within the task were not successful. Model selection was stopped if only one single model was left, the maximum number of 8 generations was reached, the minimum ${\epsilon }_{{{{\rm{ABC}}}}}=0.175$ was reached or less than 1000 accepted particles were obtained for a population. See Supplementary Code for implementation details.

### Functional testing

The ER-ESN, EXP-LSM, and LAYERED models were trained to discriminate natural texture classes, which were represented by one natural image each. Samples of length 500 pixel of these classes were obtained at random locations of these images. These samples were then fed into LAYERED networks via a single input neuron projecting to the first layer of the network. In the ER and EXP case the input neuron projected to all neurons in the network. Within the recurrent network, the dynamical model was given by ${{{\bf{a}}}}\left(t+1\right)=\left(1-\alpha \right){{{\bf{a}}}}(t)+\alpha \,{{{\rm{relu}}}}({{{\rm{C}}}}{{{\bf{a}}}}(t)+{{{\bf{u}}}}(t))$, where ${{{\rm{C}}}}$ was the adjacency matrix, ${{{\bf{u}}}}$ the input, ${{{\bf{a}}}}$ the activation, $\alpha =0.1$ the leak rate and ${{{\rm{relu}}}}\left(\bullet \right)={{{\rm{max }}}}(0,\bullet )$. Readout was a softmax layer with seven neurons ${o}_{1},\ldots ,{o}_{7}$; one neuron for each class. Adam^93^ was used to train all the forward connections with exception of the input connections. The loss $l$ was the categorical cross-entropy accumulated over the last 250 time steps $l=-\mathop{\sum}\limits_{{i,{c}}^{{\prime} }=1,..,7,{t}=250\ldots 500}{\delta }_{{c}^{{\prime} },{{{\rm{c}}}}(i)}{{{\rm{log }}}}({o}_{{c}^{{\prime} }}(t))$, where $i$ denoted the sample and ${{{\rm{c}}}}(i)$ the ground truth class of sample $i$. At prediction time the predicted class ${c}^{\ast }$ was ${c}^{\ast } = {{{\rm{argma}}}} {{{\rm{x}}}}_{{{{\rm{c}}}}\in 1,\ldots ,7}\mathop{\sum}\limits_{t=250}^{500} {o}_{c}(t)$. The model was implemented in Theano (https://deeplearning.net/software/theano) and Keras (https://keras.io) as custom recurrent layer and run on Tesla M2090 GPUs. See Supplementary Code for details of the implementation.

In the SYNFIRE model, a conductance based spiking model was used with membrane potential $\dot{v}=({v}_{{{{\rm{rest}}}}}-v)/{\tau }_{p}$ with ${\tau }_{p}=20{{{\rm{ms}}}}$, inhibitory reversal potential ${v}_{{{{\rm{reversal}}}},i}=-80\, {{{\rm{mV}}}}$, excitatory reversal potential ${v}_{{{{\rm{reversal}}}},e}=0{{{\rm{mV}}}}$, resting potential ${v}_{{{{\rm{rest}}}}}=-70\, {{{\mathrm{mV}}}}$, spiking threshold ${v}_{{{{\rm{threshold}}}}}=-55\, {{{\rm{mV}}}}$, inter pool delay ${d}_{{{{\rm{pool}}}}}\sim {{{\rm{U}}}}({0.5,2})$, excitatory intra pool jitter ${d}_{{{{\rm{jitter}}}},e}\sim {{{\rm{U}}}}({0,0.3})$ inhibitory intra pool jitter ${d}_{{{{\rm{jitter}}}},i}\sim {{{\rm{U}}}}(0.3,0.9)$, excitatory refractory period ${\tau }_{{{{\rm{ref}}}},e}=2{{{\rm{ms}}}}$ and inhibitory refractory period ${\tau }_{{{{\rm{ref}}}},i}=1{{{\rm{ms}}}}$. On spiking of presynaptic neuron $j$ the membrane potential of postsynaptic neuron $i$ was increased by ${g}_{{{{\rm{pre}}}}}({v}_{{{{\rm{reversal}}}},{{{\rm{pre}}}}}-{v}_{{{{\rm{post}}}}})$ where ${g}_{{{{\rm{pre}}}}}$ denoted the presynaptic efficacy, ${v}_{{{{\rm{reversal}}}},{{{\rm{pre}}}}}$ the presynaptic reversal potential and ${v}_{{{{\rm{post}}}}}$ the postsynaptic membrane potential. The excitatory synaptic efficacy ${g}_{e}$ and the inhibitory synaptic efficacy ${g}_{i}$ were functions of the pool size and were obtained by interpolating ${s}_{{{{\rm{pool}}}}}=[80,100,120,150,200,250,300]$, ${{{{\rm{log }}}}}_{10}({g}_{e})=[-2.1,-2.25,-2.28,-2.365,-2.6,-2.625,-2.75]$ and ${{{{\rm{log }}}}}_{10}({g}_{i})=[-0.45,-0.7,-0.763,-0.894,-1.25,-1.25,-1.5]$ linearly.

The fractional chain activation ${f}_{{{{\rm{ca}}}}}$ was calculated as follows: Let ${n}_{i}(t)$ denote the number of active neurons of pool $i$ between time $t$ and $t+\Delta t$, with $\Delta {{{\rm{t}}}}=0.1{{{\rm{ms}}}}$. Let the maximal activation be $\hat{n}(t)=\mathop{{{\max }}}\nolimits_{{{{\rm{j}}}}}{n}_{j}(t)$ and define the pool activity indicator ${\delta }_{i}\left(t\right)=I({n}_{i}\left(t\right) \, > \, \frac{{s}_{{{{\rm{pool}}}}}}{2},\hat{n}\left(t\right)={n}_{i}\left(t\right),\left|\left\{i|\hat{n}\left(t\right)={n}_{i}\left(t\right)\right\}\right|=1)$. Let the cumulative activity be ${c}_{i}\left(t\right)={\sum }_{{t}^{{\prime} }\le t}{n}_{i}\left(t\right){\delta }_{i}(t)$ and ${t}_{{{{\rm{end}}}}}={{\max }}\left\{t|{c}_{i}\left(t\right) \, < \, 1.2{s}_{{{{\rm{pool}}}}}\forall i\right\}$. The number of activated pools was $N=\left|\left\{i|\exists t \, < \, {t}_{{{{\rm{end}}}}}:{\delta }_{i}\left(t\right)=1\right\}\right|$ and the fractional chain activation ${f}_{{{{\rm{ca}}}}}=N/l$ in which $l$ was the chain length. Fractional pool activation ${f}_{{{{\rm{pa}}}}}$ at time $t$ was the fraction of neurons in a pool that exceeded a threshold activity ${v}_{{{{\rm{threshold}}}}}=-55{{{\rm{mV}}}}$ between time $t$ and $t+\Delta t$, with $\Delta t=0.1{ms}$.

Additional model-functional testing was performed. Also, SYNFIRE, FEVER, API, and STDP-SORN networks were trained to discriminate textures, analogous to the ER-ESN and EXP-LSM models. The test previously applied to the SYNFIRE model was not applied to the remaining models because the SYNFIRE model was the only integrate-and-fire model. The recombination memory test, originally proposed as part of the FEVER model, was also applied to the API model and vice versa the antiphase inhibition test, originally proposed as part of the API model was also applied to the FEVER model. These two tests were not applied to the remaining models because these lacked feature vectors. The test for uncorrelated and equally distributed activity, originally proposed as part of the STDP-SORN model, was also not applied to the remaining models because they did not feature binary threshold neurons. If a model was not able to carry out a given task due to inherent properties of that model such as, e.g., absence of feature vectors, the model was considered to fail that task.

### Training, sparsification, and connectomic separability of recurrent neural networks trained on different tasks

#### Architecture and initialization of recurrent neural networks

Recurrent neural networks (RNNs) consisting of 1800 excitatory, 200 inhibitory, and a single input neuron were trained on either a texture discrimination or a sequence memorization task (Fig. 7). Each of the 2000 neurons in the RNN received synaptic inputs from the input neuron and from all other RNN neurons. The total input to neuron *i* at time *t* was given by I_*i,t*_ = W_*i,1*_ × A_*1,t-1*_ + … + W_*i,2000*_ × A_*2000,t-1*_ + **v**_*i*_×*u*_*t*_ + b_*i*_, where W_*i,j*_ is the strength of the connection from neuron *j* to neuron *i*. Connections originating from excitatory neurons were non-negative, while connections from inhibitory neurons were non-positive. Self-innervations was prohibited (W_*i,i*_ = 0 for all *i*). A_*j,t-1*_ = max(0, min(2, I_*i,t-1*_)) is the activation of neuron *j* in at time *t-1*. The input signal *u*_*t*_ was projected to neuron *i* by connection of strength **v**_*i*_. b_*i*_ was a neuron-specific bias.

Prior to training, RNNs were initialized as follows (Fig. 7a): Neuronal activations A_*i,0*_ were set to zero. Internal connection strengths W_*j,i*_ were sampled from a truncated normal distribution (by resampling values with absolute values greater than two). If necessary, the sign of W_*j,i*_ was inverted. Connections from inhibitory neurons were rescaled such that <W_*j*_> *= 0*, where <•> denotes the average. Finally, connection strengths were rescaled to a standard deviation of (2/2001)^1/2^ (^94^). Connections from the input neuron were initialized by the same procedure. Neuronal biases were set to minus <**v**>×<**u**>.

#### Texture discrimination task

RNNs were trained to discriminate between seven different natural textures. The activity of the input neuron, *u*_*t*_, was given by the intensity values of 100 consecutive pixels in a texture image. For each texture, a different excitatory neuron was randomly chosen as output neuron. The RNNs were trained to activate an output neuron if and only if the input signal was sampled from the corresponding natural texture.

The texture images were split into training (top half), validation (third quarter), and test sets (bottom quarter). Input sequences were sampled by random uniform selection of a texture image, of a row therein, and of a pixel offset. The sequences were reversed with 50% probability. The excitatory character of the input neuron was emulated by normalizing the intensity values within each gray-scale image, clamping the values to two standard deviations and adding a bias of two.

The RNNs were trained by minimizing the cross-entropy loss on mini-batches of 128 sequences using Adam^93^ (learning rate: 0.0001, *β*_*1*_: 0.9, and *β*_*2*_: 0.999). The gradient was clipped to a norm of at most 1. Every ten gradient steps, the RNN was evaluated on a mini-batch from the validation set. If the running median of 100 validation losses did not decrease for 20,000 consecutive gradient steps, the connectivity matrix W was saved for offline analysis and then sparsified (Fig. 7a). Following^95^, connections with absolute connection strength below the 10^th^ percentile were pruned (and couldn’t be regained thereafter). The validation loss and gradient step counter were reset before training of the sparsified RNN continued (Fig. 7a).

Four RNNs were trained with different sets of initial parameters and different training sequence orders. Each RNN was trained for around 5 days and 21 h, corresponding to roughly 5.75 million training steps (Python 3.6.8, NumPy 1.16.4, TensorFlow 1.12, CUDA 9.0, CuDNN 7.4, Nvidia Tesla V100 PCIe; Fig. 7b, c).

#### Sequence memorization task

In the sequence memorization task, RNNs were trained to output learned sequences at the command of the input signal. The sequences were 100-samples-long whisker traces from^96^. The input signal determined the onset time and type of sequence to generate. The activity of the input neuron, *u*_*t*_, was initially at zero (*u*_*0*_ = 0) and switched to either +1 or −1 at a random point in time. The RNN was trained to output zero while the input is zero, to start producing sequence one at the positive edge, and to generate sequence two starting at the negatives edge in *u*_*t*_. The whisker traces were drift-corrected, such that they started and ended at zero. The amplitudes were subsequently divided by twice their standard deviation.

Training proceeded as for texture discrimination. The mean squared error was used as loss function. Four RNNs with different random initializations and different training sequence orders were each trained for roughly 15 days and 22 h, corresponding to 18.5 million training steps.

#### Analysis of RNN connectomes

Connectivity matrices were quantitatively analyzed in terms of the relative excitatory-excitatory reciprocity (rr_*ee*_), the relative excitatory-inhibitory reciprocity (rr_*ei*_), the relative inhibitory-excitatory reciprocity (rr_*ie*_), the relative inhibitory-inhibitory reciprocity (rr_*ii*_), the relative prevalence of cycles of length 5 (*r*^*(5)*^), and the in-out degree correlation (*r*_*i/o*_) (Fig. 7d–i). The connectome statistics were then further processed using MATLAB R2017b. Equality of connectome statistics across different tasks was tested using the two-sample Kolmogorov-Smirnov test. To visualize structural similarity of neural networks in two dimensions, t-SNE^97^ was applied to the six connectome statistics. For a quantitative measure of structural separability of RNNs, the connectomic distance *d*_**γ**_(C_*i*_, C_*j*_) (see “Bayesian model selection”) was computed for all pairs of RNNs. *d*_**γ**_(C_*i*_, C_*j*_) < *θ* was used to predict whether RNNs i and j were trained on the same task. The performance of this predictor was evaluated in terms of the area (*A*) under the receiver operating characteristic (ROC) curve, and accuracy. The sensitivity index d’ was computed as 2^1/2^*Z*(*A*), where *Z* is the inverse of the cumulative distribution function of the standard normal distribution.

Whether information about connection strength helps to distinguish texture discrimination and sequence memorization RNNs (Fig. 7g–i) was tested as follows: For each RNN, the configuration with average connectivity closest to 24% was further sparsified by discarding the weakest 5, 10, 15,..., 95% of connections before computing the connectome statistics. Separability of texture discrimination and sequence memorization network based on the connectome statistics was quantified as above.

### Reporting summary

Further information on research design is available in the Nature Research Reporting Summary linked to this article.

## Supplementary information

Supplementary Information

Reporting Summary

## Data Availability

The data that support the findings of this study are available at https://discriminatEM.brain.mpg.de.

## Source data

Source Data

## Code availability

All methods were implemented in Python 3 (compatible with version 3.7), unless noted otherwise. All code is available under the MIT license in the Supplementary Code file and at https://gitlab.mpcdf.mpg.de/connectomics/discriminatEM. To install and run discriminatEM please follow the instruction in the readme.pdf provided within discriminatEM_v2.zip. Detailed API and tutorial style documentation are also provided within discriminatEM_v2.zip in the HTML format (doc/index.html).
