# Supplementary material for: Cellular connectomes as arbiters of local circuit models in the cerebral cortex
Source: Nat Commun. 2021 May 13;12:2785. doi: 10.1038/s41467-021-22856-z (PMC8119988; doi:10.1038/s41467-021-22856-z)
Supplement: Supplementary file 1 — Supplementary Information [file 41467_2021_22856_MOESM1_ESM.pdf]

Supplementary Material

Detailed treatment of models: structural properties

ER-ESN. We first considered a directed Erdős-Rényi (ER) model (Fig. 2b-c) [1]. ER graphs were, for example, proposed as the computational substrate of Echo State Networks (ESNs) [2, 3, 4, 5, 6, 7, 8, 9, 10]. In these studies, single neurons were allowed to make both excitatory and inhibitory synapses. However, to obey the biological constraint of neurons establishing either excitatory or inhibitory synapses - "Dale's law", [11] - we implemented ER-ESNs to comprise separate excitatory and inhibitory neuron populations. Connection probabilities were independent of inter-soma distance (Fig. 2b, cf. the next model). The reciprocity of ER networks equals the excitatory connectivity, $r_{ee} = p_{ee}$, satisfying the experimentally observed constraints (Fig. 2c).

EXP-LSM. We next considered pairwise random but distance-dependent connectivity (Fig. 2b-c). Such architectures have been proposed as models for real-time computations without stable states (Liquid State Machines, (LSMs), [12, 13, 14, 15, 16, 17, 18, 19, 20, 21, 22, 23]. To model neuronal connectivity dependent on the distance between neuronal cell bodies, soma positions were independent and identically uniformly distributed within a L4 model cube of size 300 µm (Fig. 2a). We assumed the pairwise connection probabilities $p_e(d_s) = \exp(-d_s/\lambda_e)$, $p_i(d_s) = \exp(-d_s/\lambda_i)$, to decay exponentially with inter-soma distance d_s (Fig. 2b). The decay lengths λ_e, λ_i were adjusted to satisfy the L4 connectivity constraints (Fig. 1d). The reciprocity constraint was then found to be also satisfied (Fig. 2c). Notably, the distance dependence of the EXP-LSM model implied a reciprocity higher than in ER networks, but was still within the experimentally observed regime (Fig. 2c).

LAYERED. We next investigated the hypothesis of hierarchical, layered processing in local cortical networks (Fig. 2d). Layered models were first proposed as the multi-layer perceptron [24]. Many studies of early sensory processing, particularly visual processing made use of such models [24, 25, 26, 27, 28, 29, 30, 31, 32, 33, 34]. Pure multi-layer perceptrons lack reciprocated connections and thus violate basic circuit properties found in local cortical

networks. When instead considering a stacked recurrent neural network, however, a layered model could be made comply with the observed circuit constraints. For this, excitatory neurons were uniquely grouped into sequentially ordered layers. Connections within a layer were implemented using a pairwise connection probability $p_{e,l} > 0$. However, only unidirectional connections from one layer to the subsequent layer were allowed with pairwise connection probability $p_{e,f}$ (Fig. 2d). Inhibitory neurons were not organized in layers but provided global inhibition (Fig. 2d). Obviously, as the number of layers increases, high average reciprocity and high average pairwise connectivity become mutually exclusive (Fig. 2e), such that the number of layers in a LAYERED model is limited by the connectivity and reciprocity constraints. For $n_l = 2$ to $n_l = 4$ layers, the connectivity constraints (Fig. 2f-g) could be robustly satisfied.

SYNFIRE. We then investigated embedded synfire chains (Fig. 2h). A variety of synfire chain-like models has been proposed [27, 35, 36, 37, 38, 39, 40, 41, 42, 43, 44, 45, 46, 47, 48, 49, 50, 51]. Some of these models were defined as structurally separated neuronal pools with sequential activation chains [27, 35], similar to the LAYERED model introduced above, but for $p_{e,l} = 0$ (Fig. 2d). Most studies however considered embedded synfire chain models, in which synfire pools were allowed to overlap [35, 36, 37, 38, 43, 46, 49, 50]. We considered embedded synfire chains [50]. Excitatory synfire pools were uniformly drawn with replacement. Pools were then sequentially connected all-to-all (Fig. 2h). The neuronal populations belonging to a synfire pool could overlap (Fig. 2h) such that reciprocated connections emerged during this process (Fig. 2h). Connectivity and reciprocity were found to lie within the experimentally observed constraints for pool size s_{pool} between 80 and 300 and the number of pools n_{pool} between 60 and 190 (Fig. 2i,j).

FEVER. Then, the feature-vector recombination (FEVER) model [52, 53] was considered, which was proposed to allow sustained neuronal representations on macroscopic time scales (seconds; “short-term memory”) (Suppl. Fig. 2a). Here, connectivity is based on the neurons’ receptive fields represented by feature vectors: The postsynaptic neurons' feature vectors are additively and sparsely recombined to approximate the presynaptic neuron's own feature vector (Suppl. Fig. 2a). The FEVER model in its original formulation yielded lower pairwise connectivity (range 0-0.1) and higher reciprocity

(range 0.45-0.6) than given by the barrel circuit constraints (Suppl. Fig. 2b). We therefore constructed an adapted FEVER model by initializing the connectivity using an ER model with connectivity $p_{\text{init},c} > 0$, $c \in \{e, i\}$. Subsequently, connections were added according to the FEVER rule, which now had to incorporate the initial random connections, as well. As a consequence, the average FEVER rule error $\|\mathrm{D} - \mathrm{DW}\|$ of a modified FEVER network with $f_d = 200$ and initial excitatory connectivity 0.14 was 10% larger than the average FEVER rule error of an unmodified FEVER network with $f_d = 400$ (see Suppl. Fig. 2c). In fact, a fraction of the resulting modified FEVER model instantiations complied with the barrel circuit constraints (Suppl. Fig. 2b). However, the question whether these modified models still provided the functional properties originally proposed for the FEVER networks had to be investigated (see below).

API. Lastly, we investigated the anti-phase inhibition (API) model (Suppl. Fig. 2d), which was originally proposed to explain contrast invariant stimulus tuning in the primary visual cortex [54, 55, 56, 57, 58], and has been extended to the primary somatosensory cortex [55]. In the original API model, a hidden feature vector is associated with each neuron, and connections between neurons are determined based on a connectivity rule that depended only on the correlation between the feature vectors of the pre- and postsynaptic neurons (Suppl. Fig. 2d). The shape of this connectivity rule determined the pairwise connectivity and reciprocity. For excitatory presynaptic neurons *k* with feature vector $\mathbf{d}_k$, connections were not established with a candidate postsynaptic neuron l if the cosine similarity of their feature vectors was negative ($c_{\text{sim}}(\mathbf{d}_k, \mathbf{d}_l) < 0$), and the connection probability was set to rise steeply for positively correlated feature vectors (Suppl. Fig. 2d). Similarly for inhibitory neurons, connections were not established if the feature vector of the candidate postsynaptic neuron was positively correlated, but connection probability was set to rise steeply for increasingly negative feature vector correlations. The particular shapes of this connectivity rule as proposed in the original model, however, yielded too many reciprocated excitatory connections (Suppl. Fig. 2e-g), reciprocity range 0.8-1) since bidirectional connections between neurons with similar feature vectors were strongly favored. We therefore adapted the shape of the API connectivity rule to reduce this bias (Suppl. Fig. 2d), decreasing the frequency of reciprocal connections and satisfying the local cortical circuit constraints

for excitatory connections (Suppl. Fig. 2f,g). For inhibitory connections, the constraint to reach connectivity beyond 50% (Fig. 1d) was not fulfillable with the original API connectivity rule (Suppl. Fig. 2d,e), which yielded an effective limit at 50% connectivity. We therefore modified the API connectivity rule for inhibitory connections to allow these also between neurons with negative cosine similarity of their feature vectors (Suppl. Fig. 2d-g).

Thus, in summary, 3 of the 7 models (ER-ESN, EXP-LSM, SYNFIRE) satisfied the biological circuit constraints in a barrel module as they had been originally proposed; 4 models (LAYERED, STDP-SORN, FEVER, API) did not satisfy these constraints in their original formulation. These however were made comply with these circuit constraints by minor (LAYERED, API) or more substantial (STDP-SORN, FEVER) alterations. We had to investigate next whether these altered models could still perform the computations they had been proposed for, tested in the context of potentially relevant stimulus transformations in a barrel module.

STDP-SORN. We next considered a model in which local cortical circuity is shaped mainly by generative local learning rules such as spike timing dependent plasticity (STDP) [59, 60]; such rules have been used in networks labeled self-organizing recurrent neural networks (SORN) [51, 61, 62, 63, 64]. Here, cortical circuity is formed by STDP, synaptic normalization, intrinsic plasticity and structural plasticity (Suppl. Fig. 2h-l). The original SORN formulation yielded however substantially lower excitatory reciprocity than found experimentally (range 0-0.0038 compared to 0.15-0.45, (Suppl. Fig. 2m) and we therefore introduced the following modifications to the SORN model: originally, the STDP rule increased the synaptic weight by an amount η_{STDP} whenever the presynaptic neuron fired one time step before the postsynaptic one and decreased it by η_{STDP} if the firing order was reversed (Suppl. Fig. 2i). We modified this rule such that the synaptic weight was also increased by η_{STDP} if both neurons fired at the same time (Suppl. Fig. 2i). Originally, the structural plasticity rule prescribed a fixed number of synapse creation attempts per time step (Suppl. Fig. 2k). We introduced a homeostatic structural plasticity rule which attempted to keep the number of connections constant (Suppl. Fig. 2j,k). Synapses were added with initial weight c_{add}. The synaptic weight developed over

time following the STDP rule (Suppl. Fig. 2l). Together, these alterations of the STDP-SORN model yielded pairwise connectivity properties that complied with the experimentally observed ones in the barrel circuit (Suppl. Fig. 2m).

Detailed functional investigation of models

To investigate the models' functional properties, we considered the sensory input to a barrel module, which is dominated by the response to the aligned principal whisker [65]. An awake behaving mouse is frequently swiping its whiskers across objects with different surface texture [66, 67, 68, 69]. When doing so, neurons in thalamus and cortex have been shown to exhibit specific responses to troughs and elevations in the surfaces the whisker is exposed to ("slip – stick events", [70, 71]). We therefore considered the discrimination of surface textures, represented as height – correlated activity, as a simple realistic model test, and exposed the ER-ESN, EXP-LSM and LAYERED models to such a texture discrimination task. The API model had been already specifically proposed for rodent S1 [55], and we therefore tested the anti-phase stimulus representation property directly. The FEVER model had been proposed to allow extended representation of sensory stimuli on time scales relevant for short-term working memory, so we used the correlation between the representations in the network at the beginning and end (after 20 neuronal time constants) as a test of model functionality. Finally, the STDP-SORN and SYNFIRE models have so far been mainly tested for basic stability properties, namely the lack of correlated firing modes in SORN [62] and the faithful transmission of activity across pools in SYNFIRE [50], which we therefore considered as the required readouts for model functionality for SORN and SYNFIRE.

ER-ESN, EXP-LSM, LAYERED. The ER-ESN, EXP-LSM and LAYERED models were tested using a texture classification task (Suppl. Fig. 1a). We assumed that texture elevation Δz (Suppl. Fig. 1a,b) is converted into a temporal activity when the whisker is sweeping over that surface (Suppl. Fig. 1b, see above). Such elevation profiles were sampled from 7 textures (Suppl. Fig. 1a,c) and presented as network input activity (Suppl. Fig. 1d,e, this would correspond to thalamic input to layer 4). The neurons in the presumed L4 network were then connected to dedicated readout neurons, $o_1, \ldots, o_7$,

whose activity was interpreted as (continuous) texture classification readout (Suppl. Fig. 1e, top). In order to extract a texture class prediction from the activity of the output neurons, their activity was integrated over the second half of the texture presentation, and the highest activity was interpreted as texture choice (Suppl. Fig. 1f). For the ER-ESN and EXP-LSM models, only the readout projections from the L4 pool to $o_1, \ldots, o_7$ were trained. In the LAYERED model, all inter-layer projections $p_{e,f}$ (Fig. 2d) were trained in addition. Training was performed using the "Adam" adaptive moment estimation method [72].

While the performance of the LAYERED model was substantially higher than that of ER-ESN and EXP-LSM models (accuracies 90.8% vs 66.1%, 66.5%, Suppl. Fig. 1g), all models performed substantially above chance level (chance level 14% accuracy, Suppl. Fig. 1g).

SYNFIRE. Synfire chains were proposed as a model to explain synchronous propagation of cortical activity [35]. We therefore tested whether the initial activation of a single neuron pool in our modified SYNFIRE model still yielded a propagation of activity along the embedded SYNFIRE chain in a sequential manner (Suppl. Fig. 1h). For this we first activated all neurons in one neuron pool, and then tracked whether the subsequent pools were also activated, and in the right sequence. We considered a pool to be activated if at least half of its neurons were active (Suppl. Fig. 1i). We found that the SYNFIRE model did indeed support stable propagation within the imposed circuit constraints (Suppl. Fig. 1h,i). The average activated fraction of the chain f_{ca} was substantially above 95% for pool sizes $s_{\mathrm{pool}} \geq 100$ (Suppl. Fig. 1j) and reached about 80% for $s_{\mathrm{pool}} = 80$ (Suppl. Fig. 1j). Since the structural constraints had defined a range of pool sizes 80-300 (Fig. 2h-j), the modified SYNFIRE model was considered to still be sufficiently viable.

FEVER. For the adapted FEVER model, we evaluated whether it could faithfully maintain a given input stimulus representation over time, as proposed in the original work in the context of short-term memory [53]. We compared the imprinted state at the beginning of a trial (imposed as activity in [53] to each neuron) to the represented state at the end of a trial (using the cosine similarity as measure (Suppl. Fig. 3a,b)). In fact, the

representation was robustly stable over time for a range of feverization ratios f_r (range 0.5-1, Suppl. Fig. 3b). Thus, although the FEVER error had increased by 11% compared to the original model (Suppl. Fig. 2c), the modified FEVER network still provided its key proposed functional feature: to keep stimuli represented over macroscopic time scales within a network.

API. The originally proposed API network [54] was intended to convert the purely excitatory thalamocortical input impinging on L4 neurons (Suppl. Fig. 3c) into an intracortical activity that was inhibition-dominated for those neurons whose feature vectors were anticorrelated to that of the thalamic input (Suppl. Fig. 3d). Similarly, the intracortical synaptic input to neurons with feature vectors orthogonal to that of the thalamic input was intended to be close to zero, and the intracortical synaptic input was intended to be excitation-dominated for neurons with feature vector aligned to that of the thalamic input (Suppl. Fig. 3d). We took the inhibition-dominated regime as the key property of this network ("antiphase inhibition") and tested whether for neurons with a feature vector anticorrelated with the thalamic input feature vector in fact increasingly inhibition-dominated input could be found after $t_{\mathrm{end}} = 100$ time constants of the network simulation (Suppl. Fig. 3d). We found that also in the modified API model, neurons with a feature vector tuned antiphasic to the thalamic input were inhibited (Suppl. Fig. 3d). Since furthermore the correlation between cortical feature vectors of antiphasic tuned neurons and thalamic feature vectors decreases with increasing feature space dimension (Suppl. Fig. 3e), the API tuning property vanished for feature space dimension above about 50 (Suppl. Fig. 3e; see inset in Suppl. Fig. 3e for an example of the API tuning property for a higher feature space dimension of 93). Since this was in the range of possible thalamic feature space dimensions (note that only about 250 thalamic neurons project to a L4 barrel), we considered the API tuning property to be sufficiently realized for our adapted API model, as well.

STDP-SORN. The STDP-SORN model was proposed as an example of network self-organization, demanding that neuronal activity in this network would neither overly synchronize nor be limited to a small subset of the neuronal population (Suppl. Fig. 3f). We used a simple measure of median correlation between the activities in all pairs of

neurons in the network to detect overly synchronized network activity. We found that for learning rates η_{STDP} of 0.0006-0.0014 and intrinsic learning rates η_i of 0.05-0.1 (Suppl. Fig. 3f), this network activity correlation remained below 0.0422, indicating that the modified STDP-SORN model fulfilled this basic functional requirement. Similarly, the spike source entropy (SSE) was greater than 0.99, similar to the original work [62], for the modified STDP-SORN model indicating equally distributed activity.

In summary, the process of adapting the previously proposed models to basic experimentally determined circuit constraints in L4 of barrel cortex (Fig. 2, Suppl. Fig. 1, Suppl. Fig. 2) did not compromise the basic functional properties that these models were expected to exhibit in the context of sensory input to cortex (Suppl. Fig. 1, Suppl. Fig. 3).

Supplementary methods: functional properties of STDP-SORN, API, FEVER

The STDP-SORN model was tested for pairwise neuronal correlation. The Pearson product-moment correlation coefficients $\mathrm{C}_{n,n}$ were calculated for the activity traces of each neuron pair. The median $\mathrm{med}(\mathrm{C}_{n,n})$ of these correlations was calculated to represent the whole network. It was also tested for equal activity distribution through the spike source entropy (SSE), defined by $SSE = \frac{\sum_{k=1}^{n_e} \rho_k \log_2 \rho_k}{\log_2 1/n_e}$ in which ρ_k denoted the mean firing probability of neuron k across the complete trial.

The API model was tested for its antiphase inhibition property. The dynamical model was $a(t+1) = (1-\alpha)a(t) + \alpha C\mathrm{relu}(a(t) + u_0)$ with $\alpha = 1/10$. Neuron k received input $u_{0,k}$ given by $u_{0,k} = \mathrm{relu}(c_{\mathrm{sim}}(\mathbf{stim}, \mathbf{d}_k), 0)$. The stimulus was $\mathbf{stim} = \mathbf{d}_{k^*}$ where $k^* \sim \mathrm{DiscreteUniform}(0, n)$ was a randomly chosen neuron. At the end of a trial the feature vector-stimulus cosine similarity $c_{\mathrm{sim}}(\mathbf{d}, \mathbf{stim})$ was calculated. The simulation was implemented in Theano.

The FEVER dynamics were given by $a(t+1) = a(t) + \alpha((\mathrm{C}-1)\, f\big(a(t)\big) + u(t))$ according to [53], with $f(x) = \min(\max(x, 0), 2/10)$, $\alpha = 1/100$, $u(t) = u_0 \mathrm{I}(t < 0.05\, t_{max})$ and $\mathbf{u_0} = f(\mathbf{r})$ where $\mathbf{r}$ was a random vector with components $\mathbf{r}_k = \mathbf{u}_k/\|\mathbf{u}\|$, $\mathbf{u}_k = \mathbf{e}_k \boldsymbol{\delta}_k$

with $\mathbf{e}_k \sim \mathrm{U}(-1,1)$ iid. and $\delta_k \sim \mathrm{Ber}(1/10)$ iid.. At the end t_{end} of the trial the represented state $\mathbf{d}_{\mathrm{end}} = \mathrm{D}a(\mathrm{t}_{\mathrm{end}})$, with D the feature vector matrix, was compared to the target state $\mathbf{d}_0 = \mathrm{D}\mathbf{u}_0$ via the cosine similarity $c_{\mathrm{sim}}(\mathbf{d}_0, \mathbf{d}_{\mathrm{end}})$.

Supplementary methods: extended analysis of incomplete and erroneous circuit measurements

Edge removal only. A fraction of the edges (connections) was uniformly at random removed from the network irrespective of whether their pre- and postsynaptic neurons were excitatory or inhibitory.

Edge addition only. A fraction of the number of edges present in the unperturbed network was added between previously non connected neurons. Neuron pairs were chosen uniformly at random irrespective of whether their pre- and postsynaptic neurons were excitatory or inhibitory. Connections were then added and the sign of the added connection determined according to the type of the presynaptic neuron.

Biased perturbation. Removal and addition of edges was biased towards a subnetwork. A subnetwork consisting of a fraction f_m of the excitatory and of the same fraction f_m of the inhibitory neurons, i.e. $f\,n_e$ excitatory and $f\,n_i$ inhibitory neurons was chosen uniformly at random from all subnetworks with such excitatory and inhibitory neuron numbers. A total number of $\xi(n_e + n_i)$ connections were then first removed from the subnetwork and reinserted again.

Locally dense reconstruction of a barrel subvolume. Neurons and connections were assumed to be uniformly and independently distributed in the barrel cube of side length d_b (Fig. 2a). The locally dense reconstruction of a barrel subcube of side length d_{EM} was simulated as follows: First, the connectome was restricted to neurons located within the subcube. Each neuron had a probability $p = (d_{\mathrm{EM}}/d_b)^3$ of being located within the subcube. Of the remaining connections, the ones not located within the subcube were discarded. Each connection had a probability p of being located within the subcube. Note that the minimum acceptance distance ϵ_{ABC} was set to zero for the simulation of locally dense reconstructions.

Supplementary Figures

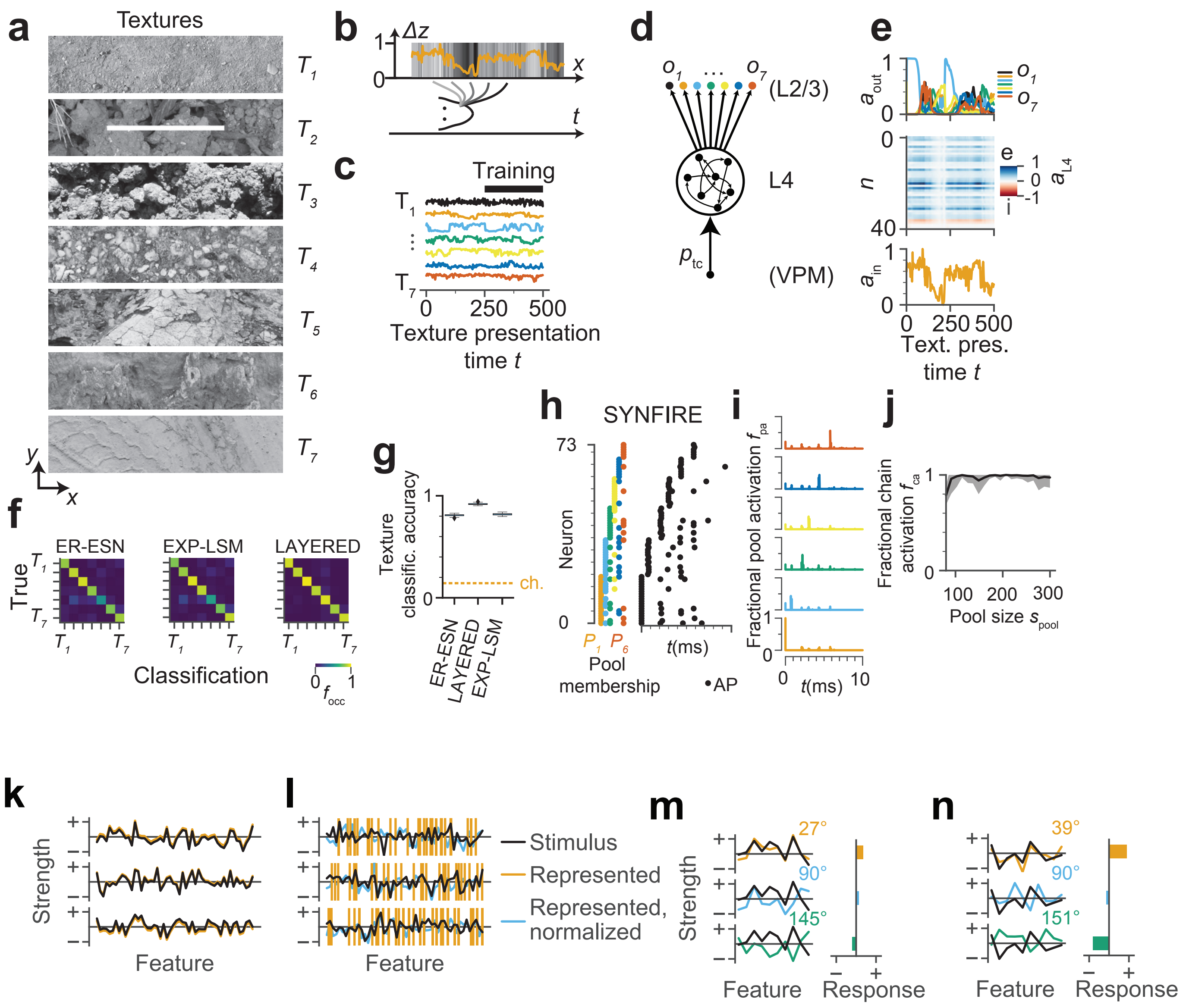

Figure S1 - Klinger et al.

Figure S1 Functional performance of candidate models in the local circuit context of a barrel in primary somatosensory cortex. **(a)** Example input to the whisker-pathway: surface texture profiles $T_1 \dots T_7$ (sampled from two dimensional (x and y) images, white line). **(b)** Sketch of transformation of the texture elevation Δz into a temporal activity trace through whisker movement. **(c)** Example texture traces from textures $T_1, \dots, T_7$ that were fed into the modeled L4 network for the ER-ESN, EXP-LSM and LAYERED models. Note that the networks were trained during the second half of the texture presentation (black line). **(d)** Discrimination of thalamocortical input by a L4 network with output to L2/3. The thalamic input was modeled as the texture traces (a-c). Models were trained to classify by output to seven classification units (for example in L2/3). **(e)** Example activities in the network: thalamocortical input signal a_{in} (bottom), intra-L4 activity a_{L4} of 40 example neurons n (excitatory neurons (*e*), blue; inhibitory neurons (*i*), red, middle), activity a_{out} of readout neurons $o_1, \dots, o_7$ potentially located in L2/3 (top). Average readout activity in the second half of presentation time was used for determining the classification result. **(f)** Classification results for the ER-ESN, EXP-LSM and LAYERED models determined from 4497 randomly selected test-set texture exposures. **(g)** Classification accuracy for the results in (f) (box plot of bootstrapped texture classification tests, *n*=100 bootstrap samples; center line, median; box limits, upper and lower quartiles; whiskers, 1.5x interquartile range; points, outliers). Note that all models provide high classification accuracy (above 80%) in this texture discrimination task, well above chance level (dashed line). **(h-j)** Functional properties of the SYNFIRE model as implemented for the model discrimination in Fig. 4-6. **(h)** Example activity (right) in an embedded SYNFIRE model with 6 pools of size s_{pool}=290 neurons each (i.e. r_{ee} of 0.15, r_{ee} of 0.33, see Fig. 2h-j). Random subset of 74 neurons (of the 1780 neurons in the circuit) and their pool membership (left) shown. Note that in the embedded SYNFIRE model, neurons belong to multiple pools. **(i)** Fractional pool activation (fraction of neurons per pool active over time) for the example in h. **(j)** Fractional chain activation over pool size. Each pool size was evaluated 200 times on networks with 2000 neurons (center line: mean; shading: s.d.). **(k)** Short-term memory in the FEVER model; three examples shown. The stimulus to be represented (black) and the state actually represented by the FEVER network (orange) are closely aligned. **(l)** Short-term memory in the API model; three examples shown. The state actually represented by the API model is dissimilar to the stimulus. Also the normalized represented state (blue) is not aligned with the stimulus. **(m)** Stimulus tuning in the FEVER model; three examples shown. All responses are weak. **(n)** Stimulus tuning in the API model; three examples shown. The aligned neuron (39° relative tuning) responds strongly positive, the anti-aligned neuron (151° relative tuning) responds strongly negative.

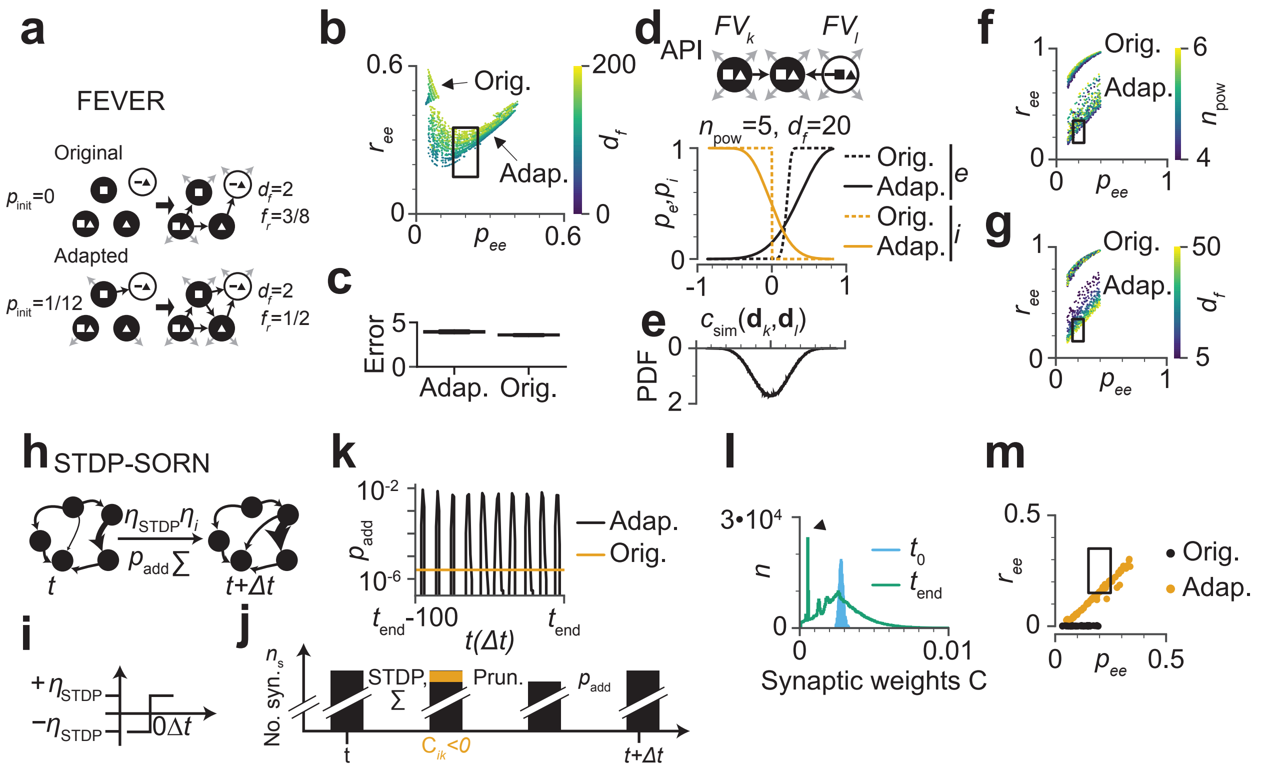

Figure S2- Klinger et al.

Figure S2 Compliance of candidate models with the so-far experimentally determined pairwise circuit constraints in L4 (see Fig. 1d): FEVER, API and STDP-SORN. **(a)** Adaptation of the feature vector recombination model (FEVER). The adapted model starts with a randomly initialized network, whereas the original model starts with an empty network. **(b)** Excitatory reciprocity r_{ee} over excitatory pairwise connectivity p_{ee} for the original and adapted FEVER model. The feature space dimension d_f is color coded. **(c)** Average FEVER error for the original and the adapted FEVER model. Errors bars indicate 95% confidence intervals from 1000 bootstrapped iterations. **(d)** Adaptation of the antiphase inhibition model (API). Excitatory and inhibitory connection probability p_e and p_i over cosine similarity of the feature vectors $c_{\mathrm{sim}}(\mathbf{d}_k, \mathbf{d}_l)$ of feature vector $\mathbf{d}_k$ and feature vector $\mathbf{d}_l$. **(e)** Distribution of cosine similarities in an API network for $n_{\mathrm{pow}} = 5$ and $d_f = 20$. **(f)** Excitatory reciprocity r_{ee} over excitatory pairwise connectivity p_{ee} for the original and adapted API model. The conversion factor n_{pow} is color coded. **(g)** Excitatory reciprocity r_{ee} over excitatory pairwise connectivity p_{ee} for the original and adapted API model. The feature space dimension d_f is color coded. **(h)** Spike timing dependent plasticity / self-organizing recurrent neural network (STDP-SORN) model. Transition from time t to time $t + \Delta t$. The network is modified through spike timing dependent plasticity with learning rate η_{STDP}, intrinsic plasticity with learning rate η_i, random synapse addition with probability p_{add}, and synaptic normalization Σ. **(i)** A synaptic weight is increased by η_{STDP} if a postsynaptic spike is emitted after an interval Δt after the presynaptic spike has occurred or if the postsynaptic spike is emitted at the same time. A synaptic weight is decreased by η_{STDP} if a presynaptic spike is emitted after an interval Δt after the postsynaptic spike. **(j)** Evolution of the number of synapses N_s from time t through time $\mathrm{t} + \Delta t$. The number of synapses n_s is not modified by the spike timing dependent plasticity STDP or the synaptic normalization Σ. The negative synapses $\mathrm{C}_{ik} < 0$ are removed in a subsequent pruning step. New synapses are then added with probability p_{add}. **(k)** Probability of adding a synapse p_{add} over time t is adjusted such that pruned synapses are replaced. **(l)** Histogram of excitatory synaptic weights C at the start t_0 and the end t_{end} of a SORN simulation. Randomly added synapses are initialized with weight c_{add} (black triangle). **(m)** Excitatory reciprocity r_{ee} over excitatory pairwise connectivity p_{ee}. Orange dots represent the adapted model, black dots the original model (black box: known barrel circuit constraints, see Fig. 1d).

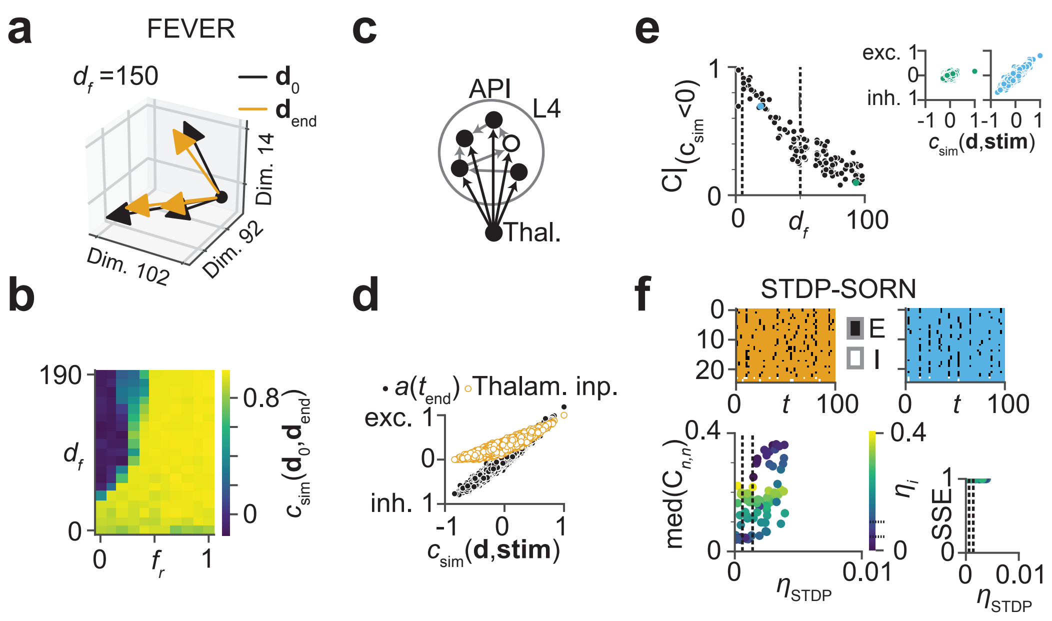

Figure S3 - Klinger et al.

Figure S3 Functional performance of candidate models in the local circuit context of a barrel in primary somatosensory cortex: FEVER, API, SDTP-SORN models. **(a)** FEVER model. Dimensions 14, 92, and 102 of the imprinted state $\mathbf{d}_0$ and the represented state at the end of the trial $\mathbf{d}_{\mathrm{end}}$ for a feature space with dimension $d_f = 150$. **(b)** Cosine similarity $c_{\mathrm{sim}}(\mathbf{d}_0, \mathbf{d}_{\mathrm{end}})$ of $\mathbf{d}_0$ and $\mathbf{d}_{\mathrm{end}}$ over feature space dimension d_f and feverization ratio f_r. **(c)** API model. **(d)** Activity at the end of the trial $a(t_{\mathrm{end}})$ and thalamic input for the API model. Excitation (exc.) and inhibition (inh.) are plotted on the ordinate, the cosine similarity between feature vector and stimulus $c_{\mathrm{sim}}(\mathbf{d}, \mathbf{stim})$ on the abscissa. **(e)** Correlation of $a(t_{\mathrm{end}})$ with $c_{\mathrm{sim}}(\mathbf{d}, \mathbf{stim})$ for those neurons with $c_{\mathrm{sim}}(\mathbf{d}, \mathbf{stim}) < 0$ as function of the feature space dimension d_f. The full data corresponding to the blue and green dots is shown in the insets **(f)** Example STDP (SORN) activity for excitatory neurons (E) and inhibitory neurons (I). Top left: Example with low median activity correlation $\mathrm{med}(\mathrm{C}_{n,n})$. Top right: Example with high median activity correlation $\mathrm{med}(\mathrm{C}_{n,n})$. Bottom left: Median activity correlation $\mathrm{med}(\mathrm{C}_{n,n})$ over STDP learning rate η_{STDP} and intrinsic learning rate η_i. The dashed bars in the scatter plot and the black lines at the color bar indicate the support of the prior distribution. Bottom right: spike source entropy.

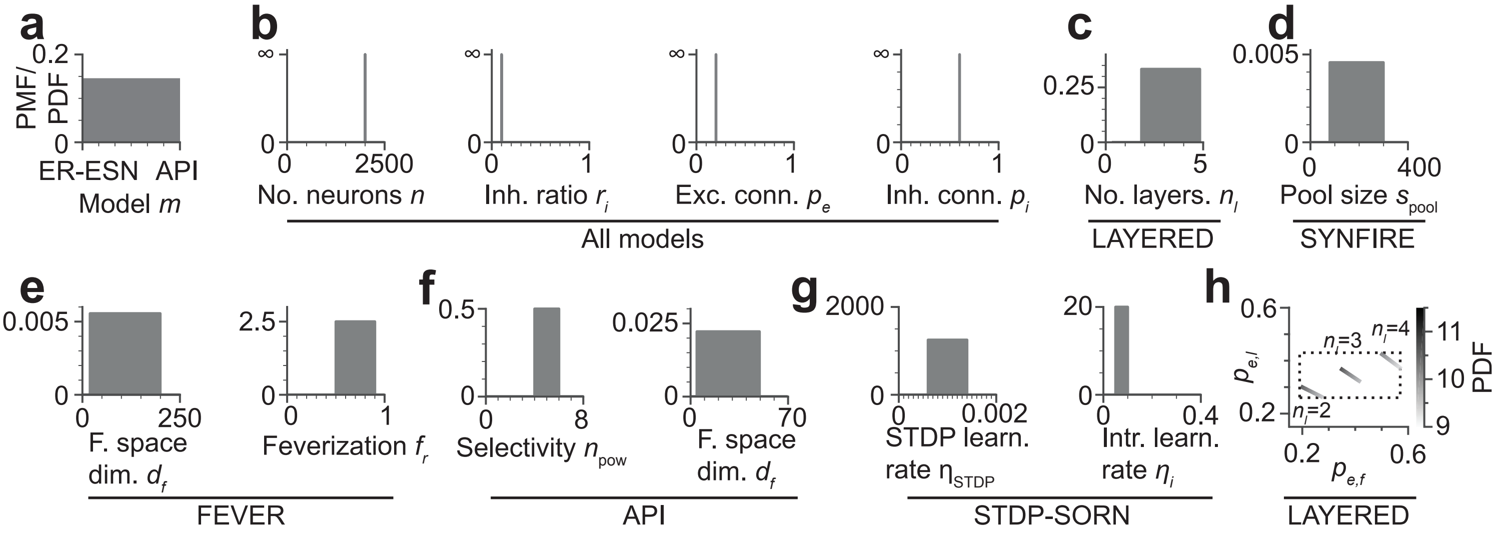

Figure S4 - Klinger et al.

Figure S4 Probability mass or density functions of the priors used for Bayesian model selection (Fig. 3). **(a)** Uniform prior over the model classes m. **(b)** Parameters shared across models: number of neurons n, inhibitory ratio r_i, excitatory connectivity p_e, and inhibitory connectivity p_i. **(c)** Number of layers n_l for the LAYERED model. **(d)** Pool size s_{pool} for the SYNFIRE model. **(e)** Feature space dimension d_f and feverization f_r for the FEVER model. **(f)** Selectivity n_{pow} and feature space dimension d_f for the API model. **(g)** STDP learning rate η_{STDP} and intrinsic learning rate η_i for the STDP-SORN model. **(h)** Excitatory forward connectivity $p_{e,f}$ and excitatory lateral connectivity $p_{e,l}$ for the LAYERED model (dotted: $p_{e,f} \times p_{e,l} \in [0.19, 0.57] \times [0.26, 0.43]$).

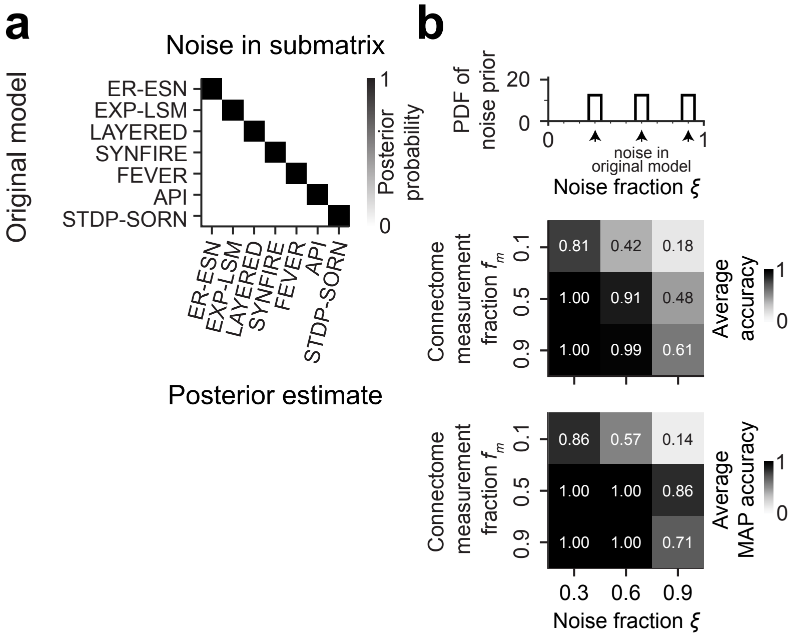

Figure S5 - Klinger et al.

Figure S5 Extended analysis of noisy and incomplete circuit measurements. Noise prior during ABC-SMC inference was of the same type as the perturbations to the measured connectome. **(a)** Biased perturbation: 15% of the connections of the unperturbed network were randomly removed from a randomly chosen sub-network of size $f_m = 30\%$ of the original network. The same amount of connections was then randomly reinserted in the same sub-network before ABC-SMC inference (*n*=1 repetition; noise prior of Beta(2,10)). **(b)** Effect of highly informative noise prior on accuracy of model selection from noisy and incomplete circuit measurements. Top: Uniform distributions centered on true noise levels with scales of 8 percentage points were used as noise priors. Middle and bottom: model selection accuracy reported as average posterior probability and maximum-a-posteriori accuracy (*n*=1 repetition per entry), respectively. Note that highly informative noise priors result in more accurate model selection compared to Beta(2, 10) noise priors (Fig. 5c,d).

Supplementary References

1. Erdős P, Rényi A. On random graphs. *Publicationes Mathematicae Debrecen* **6**, 290-297 (1959).

2. Jaeger H, Haas H. Harnessing Nonlinearity: Predicting Chaotic Systems and Saving Energy in Wireless Communication. *Science* **304**, 78-80 (2004).

3. Buehner M, Young P. A tighter bound for the echo state property. *IEEE Transactions on Neural Networks* **17**, 820–824 (2006).

4. Xue Y, Yang L, Haykin S. Decoupled echo state networks with lateral inhibition. *Neural Networks* **20**, 365-376 (2007).

5. Lukoševičius M, Jaeger H. Reservoir computing approaches to recurrent neural network training. *Computer Science Review* **3**, 127–149 (2009).

6. Jaeger H. The “echo state” approach to analysing and training recurrent neural networks-with an erratum note. *Bonn, Germany: German National Research Center for Information Technology GMD Technical Report* **148**, 34 (2010).

7. Pascanu R, Jaeger H. A neurodynamical model for working memory. *Neural Networks* **24**, 199-207 (2011).

8. Lukoševičius M, Jaeger H, Schrauwen B. Reservoir Computing Trends. *KI - Künstliche Intelligenz* **26**, 365-371 (2012).

9. Yildiz IB, Jaeger H, Kiebel SJ. Re-visiting the echo state property. *Neural Networks* **35**, 1-9 (2012).

10. Manjunath G, Jaeger H. Echo state property linked to an input: Exploring a fundamental characteristic of recurrent neural networks. *Neural computation* **25**, 671–696 (2013).

11. Dale H. Pharmacology and Nerve-endings (Walter Ernest Dixon Memorial Lecture): (Section of Therapeutics and Pharmacology). *Proceedings of the Royal Society of Medicine* **28**, 319-332 (1935).

12. Maass W, Natschläger T, Markram H. Real-Time Computing Without Stable States: A New Framework for Neural Computation Based on Perturbations. *Neural Computation* **14**, 2531-2560 (2002).

13. Maass W, Markram H. On the computational power of circuits of spiking neurons. *Journal of Computer and System Sciences* **69**, 593-616 (2004).

14. Maass W, Natschläger T, Markram H. Computational models for generic cortical microcircuits. *Computational neuroscience: A comprehensive approach* **18**, 575 (2004).

15. Maass W, Natschläger T, Markram H. Fading memory and kernel properties of generic cortical microcircuit models. *Journal of Physiology-Paris* **98**, 315-330 (2004).

16. Maass W, Joshi P, Sontag ED. Principles of real-time computing with feedback applied to cortical microcircuit models. *Advances in neural information processing systems* **18**, 835 (2006).

17. Auer P, Burgsteiner H, Maass W. A learning rule for very simple universal approximators consisting of a single layer of perceptrons. *Neural Networks* **21**, 786-795 (2008).

18. Buonomano DV, Maass W. State-dependent computations: spatiotemporal processing in cortical networks. *Nature Reviews Neuroscience* **10**, 113-125 (2009).

19. Sussillo D, Abbott LF. Generating Coherent Patterns of Activity from Chaotic Neural Networks. *Neuron* **63**, 544-557 (2009).

20. Manevitz L, Hazan H. Stability and Topology in Reservoir Computing. In: *Advances in Soft Computing* (ed^(eds Sidorov G, Aguirre AH, García CAR). Springer (2010).

21. Hazan H, Manevitz LM. Topological constraints and robustness in liquid state machines. *Expert Systems with Applications* **39**, 1597-1606 (2012).

22. Probst D, Maass W, Markram H, Gewaltig M-O. Liquid Computing in a Simplified Model of Cortical Layer IV: Learning to Balance a Ball. In: *Artificial Neural Networks and Machine Learning – ICANN 2012* (ed^(eds Villa AEP, Duch W, Érdi P, Masulli F, Palm G). Springer (2012).

23. Sussillo D, Barak O. Opening the Black Box: Low-Dimensional Dynamics in High-Dimensional Recurrent Neural Networks. *Neural Computation* **25**, 626-649 (2012).

24. Rosenblatt F. *Principles of neurodynamics; perceptrons and the theory of brain mechanisms.* Spartan Books (1962).

25. Fukushima K. Neural Network Model for a Mechanism of Pattern Recognition Unaffected by Shift in Position- Neocognitron. *ELECTRON & COMMUN JAPAN* **62**, 11–18 (1979).

26. Fukushima K. Neocognitron: A self-organizing neural network model for a mechanism of pattern recognition unaffected by shift in position. *Biological Cybernetics* **36**, 193-202 (1980).

27. Griffith JS. On the Stability of Brain-Like Structures. *Biophysical Journal* **3**, 299-308 (1963).

28. Hubel DH, Wiesel TN. Receptive fields, binocular interaction and functional architecture in the cat's visual cortex. *The Journal of Physiology* **160**, 106-154 (1962).

29. Ivakhnenko AG. Polynomial Theory of Complex Systems. *IEEE Transactions on Systems, Man and Cybernetics* **SMC-1**, 364-378 (1971).

30. Ivakhnenko AG, Lapa VG. *Cybernetic predicting devices.* Joint Publications Research Service [available from the Clearinghouse for Federal Scientific and Technical Information] (1965).

31. LeCun Y*, et al.* Backpropagation Applied to Handwritten Zip Code Recognition. *Neural Computation* **1**, 541-551 (1989).

32. Lecun Y, Bottou L, Bengio Y, Haffner P. Gradient-based learning applied to document recognition. *Proceedings of the IEEE* **86**, 2278-2324 (1998).

33. Malsburg CVD. Frank Rosenblatt: Principles of Neurodynamics: Perceptrons and the Theory of Brain Mechanisms. In: *Brain Theory* (ed^(eds Palm DG, Aertsen DA). Springer (1986).

34. Schmidhuber J. Deep Learning in Neural Networks: An Overview. *Neural Networks* **61**, 85-117 (2015).

35. Abeles M. *Local Cortical Circuits*. Springer (1982).

36. Abeles M. *Corticonics: Neural Circuits of the Cerebral Cortex*. Cambridge University Press (1991).

37. Bienenstock E. A model of neocortex. *Network: Computation in Neural Systems* **6**, 179-224 (1995).

38. Herrmann M, Hertz JA, Prügel-Bennett A. Analysis of synfire chains. *Network: Computation in Neural Systems* **6**, 403-414 (1995).

39. Hertz J, Prügel-Bennett A. Learning synfire chains: turning noise into signal. *International Journal of Neural Systems* **7**, 445-450 (1996).

40. Diesmann M, Gewaltig M-O, Aertsen A. Stable propagation of synchronous spiking in cortical neural networks. *Nature* **402**, 529-533 (1999).

41. Gewaltig M-O, Diesmann M, Aertsen A. Propagation of cortical synfire activity: survival probability in single trials and stability in the mean. *Neural Networks* **14**, 657-673 (2001).

42. Levy N, Horn D, Meilijson I, Ruppin E. Distributed synchrony in a cell assembly of spiking neurons. *Neural Networks* **14**, 815-824 (2001).

43. Aviel Y, Mehring C, Abeles M, Horn D. On Embedding Synfire Chains in a Balanced Network. *Neural Computation* **15**, 1321-1340 (2003).

44. Mehring C, Hehl U, Kubo M, Diesmann M, Aertsen A. Activity dynamics and propagation of synchronous spiking in locally connected random networks. *Biological Cybernetics* **88**, 395-408 (2003).

45. Abeles M, Hayon G, Lehmann D. Modeling Compositionality by Dynamic Binding of Synfire Chains. *Journal of Computational Neuroscience* **17**, 179-201 (2004).

46. Aviel Y, Horn D, Abeles M. Memory Capacity of Balanced Networks. *Neural Computation* **17**, 691-713 (2005).

47. Goedeke S, Diesmann M. The mechanism of synchronization in feed-forward neuronal networks. *New Journal of Physics* **10**, 015007 (2008).

48. Kumar A, Rotter S, Aertsen A. Conditions for propagating synchronous spiking and asynchronous firing rates in a cortical network model. *J Neurosci* **28**, 5268-5280 (2008).

49. Schrader S, Grün S, Diesmann M, Gerstein GL. Detecting Synfire Chain Activity Using Massively Parallel Spike Train Recording. *Journal of Neurophysiology* **100**, 2165-2176 (2008).

50. Trengove C, Leeuwen Cv, Diesmann M. High-capacity embedding of synfire chains in a cortical network model. *Journal of Computational Neuroscience* **34**, 185-209 (2012).

51. Zheng P, Triesch J. Robust development of synfire chains from multiple plasticity mechanisms. *Frontiers in Computational Neuroscience* **8**, 66 (2014).

52. Druckmann S, Chklovskii DB. Over-complete representations on recurrent neural networks can support persistent percepts. *Advances in neural information processing systems* **23**, 541–549 (2010).

53. Druckmann S, Chklovskii Dmitri B. Neuronal Circuits Underlying Persistent Representations Despite Time Varying Activity. *Current Biology* **22**, 2095-2103 (2012).

54. Troyer TW, Krukowski AE, Priebe NJ, Miller KD. Contrast-invariant orientation tuning in cat visual cortex: thalamocortical input tuning and correlation-based intracortical connectivity. *J Neurosci* **18**, 5908-5927 (1998).

55. Miller KD, Pinto DJ, Simons DJ. Processing in layer 4 of the neocortical circuit: new insights from visual and somatosensory cortex. *Current Opinion in Neurobiology* **11**, 488-497 (2001).

56. Hansel D, van Vreeswijk C. How noise contributes to contrast invariance of orientation tuning in cat visual cortex. *Journal of Neuroscience* **22**, 5118-5128 (2002).

57. Kayser AS, Miller KD. Opponent Inhibition: A Developmental Model of Layer 4 of the Neocortical Circuit. *Neuron* **33**, 131-142 (2002).

58. Hansen T, Neumann H. A simple cell model with dominating opponent inhibition for robust image processing. *Neural Networks* **17**, 647-662 (2004).

59. Gerstner W, Kempter R, van Hemmen JL, Wagner H. A neuronal learning rule for sub-millisecond temporal coding. *Nature* **383**, 76-78 (1996).

60. Markram H, Lübke J, Frotscher M, Sakmann B. Regulation of Synaptic Efficacy by Coincidence of Postsynaptic APs and EPSPs. *Science* **275**, 213-215 (1997).

61. Lazar A, Pipa G, Triesch J. Fading memory and time series prediction in recurrent networks with different forms of plasticity. *Neural Networks* **20**, 312-322 (2007).

62. Lazar A, Pipa G, Triesch J. SORN: a self-organizing recurrent neural network. *Frontiers in Computational Neuroscience* **3**, 23 (2009).

63. Zheng P, Dimitrakakis C, Triesch J. Network self-organization explains the statistics and dynamics of synaptic connection strengths in cortex. *PLoS Comput Biol* **9**, e1002848 (2013).

64. Aswolinskiy W, Pipa G. RM-SORN: a reward-modulated self-organizing recurrent neural network. *Frontiers in Computational Neuroscience* **9**, 36 (2015).

65. Brecht M, Sakmann B. Dynamic representation of whisker deflection by synaptic potentials in spiny stellate and pyramidal cells in the barrels and septa of layer 4 rat somatosensory cortex. *J Physiol* **543**, 49-70 (2002).

66. von Heimendahl M, Itskov PM, Arabzadeh E, Diamond ME. Neuronal activity in rat barrel cortex underlying texture discrimination. *PLoS Biol* **5**, e305 (2007).

67. Prigg T, Goldreich D, Carvell GE, Simons DJ. Texture discrimination and unit recordings in the rat whisker/barrel system. *Physiology & behavior* **77**, 671–675 (2002).

68. Carvell GE, Simons DJ. Biometric analyses of vibrissal tactile discrimination in the rat. *J Neurosci* **10**, 2638-2648 (1990).

69. Guić-Robles E, Valdivieso C, Guajardo G. Rats can learn a roughness discrimination using only their vibrissal system. *Behavioural Brain Research* **31**, 285-289 (1989).

70. Jadhav SP, Wolfe J, Feldman DE. Sparse temporal coding of elementary tactile features during active whisker sensation. *Nature Neuroscience* **12**, 792-800 (2009).

71. Wolfe J, Hill DN, Pahlavan S, Drew PJ, Kleinfeld D, Feldman DE. Texture coding in the rat whisker system: slip-stick versus differential resonance. *PLoS biology* **6**, e215 (2008).

72. Kingma D, Ba J. Adam: A Method for Stochastic Optimization. *arXiv:14126980*, (2014).
