# Supplementary material for: Cellular connectomes as arbiters of local circuit models in the cerebral cortex
Source: Nat Commun. 2021 May 13;12:2785. doi: 10.1038/s41467-021-22856-z (PMC8119988; doi:10.1038/s41467-021-22856-z)
Supplement: Supplementary file 3 — Source Data [file 41467_2021_22856_MOESM3_ESM.zip › doc/genindex.html]

Index — discriminatEM documentation

# Index

**\_**
| **A**
| **B**
| **C**
| **D**
| **E**
| **F**
| **G**
| **H**
| **I**
| **K**
| **L**
| **M**
| **N**
| **O**
| **P**
| **R**
| **S**
| **T**
| **U**
| **W**
| **Z**

## \_

|  |  |
| --- | --- |
| - \_\_call\_\_() (abcsmc.ConstantEpsilon method)   - (abcsmc.DistanceFunction method)   - (abcsmc.Epsilon method)   - (abcsmc.ListEpsilon method)   - (abcsmc.MedianEpsilon method)   - (abcsmc.PCADistanceFunction method)   - (abcsmc.RangeEstimatorDistanceFunction method)   - (abcsmc.ZScoreDistanceFunction method)   - (connectome.function.modeltest.Criterion method)   - (connectome.function.modeltest.Task method) | - \_shuffle\_subpopulation() (connectome.noise.edgeshufflingnoise.EdgeShufflingNoise method) |

## A

|  |  |
| --- | --- |
| - ABCLoader (class in abcsmc) - abcsmc   - module - ABCSMC (class in abcsmc) - add() (connectome.function.modeltest.TestSuiteResult method) - add\_criterion() (connectome.function.modeltest.TestSuite method) | - add\_random\_variables() (abcsmc.Kernel method) - adjacency\_matrix() (connectome.model.network.Network property) - API (class in connectome.model.api) - append() (connectome.function.modeltest.TestSuiteResultList method) - append\_population() (abcsmc.History method) - average\_mass\_at\_tround\_truth() (abcsmc.ABCLoader method) |

## B

|  |  |
| --- | --- |
| - BarrelCutNoise (class in connectome.noise.barrelcutnoise) | - binarize() (connectome.model.network.Network method) - BlockStructure (class in connectome.model.block) |

## C

|  |  |
| --- | --- |
| - cdf() (abcsmc.LowerBoundDecorator method)   - (abcsmc.RV method)   - (abcsmc.RVBase method)   - (abcsmc.RVDecorator method) - component (abcsmc.RVDecorator attribute) - confusion\_matrices\_table() (abcsmc.ABCLoader property) - confusion\_matrix\_dict() (abcsmc.ABCLoader method) - ConnectivityEstimator (class in connectome.analysis.connectivity) - connectome.function   - module - connectome.function.criterion   - module | - connectome.noise   - module - ConstantEpsilon (class in abcsmc) - construct\_graph() (connectome.noise.combined\_noise.RemoveAddNoiseAndSubsample method) - copy() (abcsmc.Distribution method)   - (abcsmc.LowerBoundDecorator method)   - (abcsmc.Parameter method)   - (abcsmc.RV method)   - (abcsmc.RVBase method)   - (abcsmc.RVDecorator method)   - (connectome.model.network.Network method) - Criterion (class in connectome.function.modeltest) |

## D

|  |  |
| --- | --- |
| - decorator\_repr() (abcsmc.LowerBoundDecorator method)   - (abcsmc.RVDecorator method) - DefaultContext (class in parallel) - DistanceFunction (class in abcsmc) - DistanceFunctionWithMeasureList (class in abcsmc) | - distribution (abcsmc.RV attribute) - Distribution (class in abcsmc) - do\_not\_stop\_when\_only\_single\_model\_alive() (abcsmc.ABCSMC method) - done() (abcsmc.History method)   - (connectome.function.modeltest.TestSuiteResultList method) |

## E

|  |  |
| --- | --- |
| - edge\_removal\_probability() (connectome.noise.barrelcutnoise.BarrelCutNoise method) - EdgeShufflingNoise (class in connectome.noise.edgeshufflingnoise) - EmptyMultivariateMultiTypeNormalDistribution (class in abcsmc) - enqueue\_model() (connectome.function.modeltest.TestSuite method) | - Epsilon (class in abcsmc) - ER (class in connectome.model.er) - ERFEVER (class in connectome.model.fever) - execute\_enqueued\_models() (connectome.function.modeltest.TestSuite method) - EXP (class in connectome.model.exp) |

## F

|  |  |
| --- | --- |
| - FEVERInitialConnectivity (connectome.model.fever.ERFEVER attribute) | - from\_dictionary() (abcsmc.RV static method) - from\_dictionary\_of\_dictionaries() (abcsmc.Distribution static method) |

## G

|  |  |
| --- | --- |
| - get\_block() (connectome.model.network.Network method) - get\_complete\_population\_median() (abcsmc.History method) - get\_config() (abcsmc.ConstantEpsilon method)   - (abcsmc.DistanceFunction method)   - (abcsmc.DistanceFunctionWithMeasureList method)   - (abcsmc.Epsilon method)   - (abcsmc.ListEpsilon method)   - (abcsmc.MedianEpsilon method)   - (abcsmc.PercentileDistanceFunction method)   - (abcsmc.RangeEstimatorDistanceFunction method) | - get\_cov() (abcsmc.History static method) - get\_data() (connectome.function.task.texture.TextureTask method) - get\_distribution() (abcsmc.History method) - get\_model\_probabilities() (abcsmc.History method) - get\_parameter\_names() (abcsmc.Distribution method) - get\_parameter\_std() (abcsmc.History method) - get\_results() (abcsmc.History method) - get\_results\_distribution() (abcsmc.History method) - get\_statistics() (abcsmc.History method) - GreaterThan (class in connectome.function.criterion) - group\_parameters() (abcsmc.ABCLoader property) |

## H

|  |
| --- |
| - History (class in abcsmc) |

## I

|  |  |
| --- | --- |
| - InAndOutDegreePreservingNoise (class in connectome.noise.inandoutdegreepreservingnoise) - initialize() (abcsmc.DistanceFunction method)   - (abcsmc.DistanceFunctionWithMeasureList method)   - (abcsmc.Epsilon method)   - (abcsmc.MedianEpsilon method)   - (abcsmc.PCADistanceFunction method)   - (abcsmc.RangeEstimatorDistanceFunction method) | - InOutDegreeCorrelation (class in connectome.analysis.inoutdegreecorrelation) |

## K

|  |
| --- |
| - Kernel (class in abcsmc) |

## L

|  |  |
| --- | --- |
| - LessThan (class in connectome.function.criterion) - ListEpsilon (class in abcsmc) - LL (class in connectome.model.ll) | - lower() (abcsmc.MinMaxDistanceFunction static method)   - (abcsmc.PercentileDistanceFunction static method)   - (abcsmc.RangeEstimatorDistanceFunction static method) - LowerBoundDecorator (class in abcsmc) |

## M

|  |  |
| --- | --- |
| - map() (parallel.SGE method)   - (parallel.Slurm method) - mapper (connectome.function.modeltest.TestSuite attribute) - max\_nr\_populations() (abcsmc.ABCLoader property) - maximum\_a\_posteriori() (abcsmc.ABCLoader method) - maxs() (abcsmc.ABCLoader property) - means() (abcsmc.ABCLoader method) - measures\_to\_use (abcsmc.DistanceFunctionWithMeasureList attribute) - MedianEpsilon (class in abcsmc) - MemoryTask (class in connectome.function.task.memory) | - MinMaxDistanceFunction (class in abcsmc) - model\_names() (abcsmc.ABCLoader property) - ModelPerturbationKernel (class in abcsmc) - module   - abcsmc   - connectome.function   - connectome.function.criterion   - connectome.noise   - parallel - MultivariateMultiTypeNormalDistribution() (in module abcsmc) |

## N

|  |  |
| --- | --- |
| - Network (class in connectome.model.network) - NetworkModelMixin (class in connectome.model.bases) - NonEmptyMultivariateMultiTypeNormalDistribution (class in abcsmc) - normalize() (connectome.model.network.Network method) - nr\_exc() (connectome.model.bases.NetworkModelMixin property)   - (connectome.model.block.BlockStructure property)   - (connectome.model.network.Network property) | - nr\_inh() (connectome.model.bases.NetworkModelMixin property)   - (connectome.model.block.BlockStructure property)   - (connectome.model.network.Network property) - nr\_neurons() (connectome.model.network.Network property) - nr\_of\_models\_alive() (abcsmc.History method) - nr\_simulations (abcsmc.History attribute) |

## O

|  |
| --- |
| - OutDegreePreservingNoise (class in connectome.noise.outdegreepreservingnoise) |

## P

|  |  |
| --- | --- |
| - p\_0() (connectome.model.exp.EXP static method) - parallel   - module - Parameter (class in abcsmc) - particles\_of\_population() (abcsmc.ABCLoader method) - passed() (connectome.function.modeltest.TestSuiteResult property)   - (connectome.function.modeltest.TestSuiteResultList property) - PCADistanceFunction (class in abcsmc) - pdf() (abcsmc.Distribution method)   - (abcsmc.EmptyMultivariateMultiTypeNormalDistribution method)   - (abcsmc.Kernel method)   - (abcsmc.LowerBoundDecorator method)   - (abcsmc.NonEmptyMultivariateMultiTypeNormalDistribution method)   - (abcsmc.RV method)   - (abcsmc.RVBase method)   - (abcsmc.RVDecorator method) | - PERCENTILE (abcsmc.PercentileDistanceFunction attribute) - PercentileDistanceFunction (class in abcsmc) - pes() (in module connectome.pes.strong\_path\_enumeration\_sampling) - pmf() (abcsmc.LowerBoundDecorator method)   - (abcsmc.ModelPerturbationKernel method)   - (abcsmc.RV method)   - (abcsmc.RVBase method)   - (abcsmc.RVDecorator method) - ProfilingContext (class in parallel) - PropagationTask (class in connectome.function.task.propagation.task) |

## R

|  |  |
| --- | --- |
| - random\_relabel\_neurons() (connectome.model.network.Network method) - Range (class in connectome.function.criterion) - RangeEstimatorDistanceFunction (class in abcsmc) - ReciprocityEstimator (class in connectome.analysis.reciprocity) - RelativeCycleAnalysis (class in connectome.analysis.relativecycleanalysis) - RelativeReciprocityEstimator (class in connectome.analysis.relativereciprocity) - remove\_neurons() (connectome.model.network.Network method) - RemoveAddNoiseAndSubsample (class in connectome.noise.combined\_noise) - RemoveAndAddEdgesNoise (class in connectome.noise.removeandaddedgesnoise) - results() (abcsmc.ABCLoader method) - results\_list() (connectome.function.modeltest.TestSuiteResultList property) - run() (abcsmc.ABCSMC method) | - RV (class in abcsmc) - RVBase (class in abcsmc) - RVDecorator (class in abcsmc) - rvs() (abcsmc.Distribution method)   - (abcsmc.EmptyMultivariateMultiTypeNormalDistribution method)   - (abcsmc.Kernel method)   - (abcsmc.LowerBoundDecorator method)   - (abcsmc.ModelPerturbationKernel method)   - (abcsmc.NonEmptyMultivariateMultiTypeNormalDistribution method)   - (abcsmc.RV method)   - (abcsmc.RVBase method)   - (abcsmc.RVDecorator method) |

## S

|  |  |
| --- | --- |
| - sample\_from\_models() (abcsmc.History method) - sample\_from\_population() (abcsmc.History method) - sample\_from\_prior() (abcsmc.ABCSMC method) - sanitize\_sample\_from\_prior() (abcsmc.DistanceFunctionWithMeasureList method) - set\_data() (abcsmc.ABCSMC method) - SGE (class in parallel) - sge\_available() (in module parallel) - shuffle\_graph() (in module connectome.shuffling.uniform\_degree\_preserving\_shuffling) | - signed\_to\_positive\_correlations() (connectome.model.api.API method) - Slurm (class in parallel) - slurm\_available() (in module parallel) - SORN (class in connectome.model.sorn) - SQLDataStore (class in abcsmc) - start\_end() (connectome.model.block.BlockStructure method) - store\_initial\_data() (abcsmc.History method) - Subsampling (class in connectome.noise.subsampling) - SYN (class in connectome.model.syn) |

## T

|  |  |
| --- | --- |
| - t() (abcsmc.History property) - Task (class in connectome.function.modeltest) - terminated\_abc\_smc\_ids() (abcsmc.ABCLoader method) - test\_model() (connectome.function.modeltest.TestSuite method) - TestResult (class in connectome.function.modeltest) - TestSuite (class in connectome.function.modeltest) - TestSuiteResult (class in connectome.function.modeltest) | - TestSuiteResultList (class in connectome.function.modeltest) - TextureTask (class in connectome.function.task.texture) - to\_json() (abcsmc.DistanceFunction method)   - (abcsmc.Epsilon method) - total\_nr\_simulations() (abcsmc.History property) - TrueCriterion (class in connectome.function.criterion) - TuningTask (class in connectome.function.task.tuning) |

## U

|  |  |
| --- | --- |
| - UnsynchronizedActivityTask (class in connectome.function.task.unsynchronized) - update\_random\_variables() (abcsmc.Distribution method) | - upper() (abcsmc.MinMaxDistanceFunction static method)   - (abcsmc.PercentileDistanceFunction static method)   - (abcsmc.RangeEstimatorDistanceFunction static method) |

## W

|  |  |
| --- | --- |
| - wait() (connectome.function.modeltest.TestSuiteResultList method) | - weak\_edge\_pes() (in module connectome.pes.weak\_path\_enumeration\_sampling) |

## Z

|  |
| --- |
| - ZScoreDistanceFunction (class in abcsmc) |

# discriminatEM

### Navigation

- Installation
- Model selection from the command line with discriminatEM
- Quickstart
- The connectome package
- License

- Connectome models
- Connectome analysis
- Connectome noise
- Network shuffling
- Path enumeration sampling
- Connectome builder
- Connectome function
- Connectome ABC Tasks
- ABC-SMC
- Parallel job execution
- RNN

### Related Topics

- Documentation overview

### Quick search

©2017, Emmanuel Klinger, Carsten Marr, Fabian J. Theis, Moritz Helmstaedter.
|
Powered by Sphinx 3.5.4
& Alabaster 0.7.12
