# Supplementary material for: Cellular connectomes as arbiters of local circuit models in the cerebral cortex
Source: Nat Commun. 2021 May 13;12:2785. doi: 10.1038/s41467-021-22856-z (PMC8119988; doi:10.1038/s41467-021-22856-z)
Supplement: Supplementary file 3 — Source Data [file 41467_2021_22856_MOESM3_ESM.zip › doc/connectome_model.html]

Connectome models — discriminatEM documentation

# Connectome models¶

This package provides connectome models.

*class* `connectome.model.bases.``NetworkModelMixin`¶
:   Mixin class for network models.

    Every network models derives from this class.

    Parameters
    :   - **p\_inh** – float in [0, 1]
          The inhibitory connectivity.
        - **p\_exc** – float in [0, 1]
          The excitatory connectivity.
        - **nr\_neurons** – integer >= 1
          The total number of neurons.
        - **inh\_ratio** – float im [0, 1]
          The fraction of inhibitory neurons.

    The output of this object is a `Network`

    *property* `nr_exc`¶
    :   Number of excitatory neurons.

    *property* `nr_inh`¶
    :   Number of inhibitory neurons.

*class* `connectome.model.network.``Network`(*adjacency\_matrix=None*, *nr\_exc=None*, *nr\_inh=None*, *blocks=None*)¶
:   A thin wrapper around a numpy.ndarray.

    Supports excitatory and inhibitory neurons and performs additional sanity checks.

    Note: Diagonal entries are **allowed**.

    Network objects support views of the form network[start, end] where start, end are “E” or “I”.

    If called with nr\_exc and nr\_inh a zeros filled adjacency matrix is created.

    Parameters
    :   - **adjacency\_matrix** (*array*) – The adjacency matrix.
        - **nr\_exc** (*optional int*) – Number excitatory neurons.
        - **nr\_inh** (*optional int*) – Number excitatory neurons.
        - **blocks** (optional `BlockStructure`) – The block structure of the network.

    *property* `adjacency_matrix`¶
    :   A view on the underlying adjacency matrix.

    `binarize`()¶
    :   Set any nonzero excitatory weight to 1 and any nonzero inhibitory weight to -1.

    `copy`()¶
    :   Returns
        :   A copy of the network.

        Return type
        :   Network

    `get_block`(*\**, *target\_ix*, *source\_ix*)¶
    :   Return a view on a block.

        Parameters
        :   - **target\_ix** (*int*) – Target block number.
            - **source\_ix** (*int*) – Source block number.

        Returns
        :   **view\_on\_subarray** – The view on the subarray.

        Return type
        :   ndarray

    `normalize`(*in\_out='in'*, *exc=1*, *inh=1*)¶
    :   Normalize incoming or outgoing weights.

        Parameters
        :   - **n\_out** (*str**,* *"in"* *or* *"out"*) – Whether to normalize the incoming or outgoing weights.
            - **exc** (*float*) – The weight to which to normalize excitatory neurons.
            - **inh** (*float*) – The weight to which to normalize inhibitory neurons.

    *property* `nr_exc`¶
    :   Number of excitatory neurons.

    *property* `nr_inh`¶
    :   Number of inhibitory neurons.

    *property* `nr_neurons`¶
    :   Total number of neurons in the network.

    `random_relabel_neurons`()¶
    :   Randomly relabel all neurons.

    `remove_neurons`(*neuron\_numbers*)¶
    :   Remove neurons from the network.

        Parameters
        :   **neuron\_numbers** (*list*) – A list of the neuron indices which to remove.

*class* `connectome.model.block.``BlockStructure`(*initlist=None*)¶
:   *property* `nr_exc`¶
    :   Number of excitatory blocks.

    *property* `nr_inh`¶
    :   Number of inhibitory blocks.

    `start_end`(*index*)¶
    :   Parameters
        :   **index** (*int*) – The number of the block.

        Returns
        :   **start, end** – Start and end neuron index of the block.

        Return type
        :   int, int

*class* `connectome.model.er.``ER`(*\*\*kwargs*)¶
:   **Inputs:** p\_inh, p\_exc, nr\_neurons, inh\_ratio

    Directed Erdős–Rényi network with excitatory and inhibitory subpopulations.

*class* `connectome.model.exp.``EXP`(*\*\*kwargs*)¶
:   **Inputs:** decay, p\_inh, p\_exc, nr\_neurons, inh\_ratio

    Network with exponentially decaying connectivity (liquid state machine).

    Parameters
    :   **decay** – Float in [0, 1]. If set to 1, the decay is strongest. If set to 0 there is no decay at all.

    *static* `p_0`(*decay*, *target\_connectivity*)¶
    :   Connectivty \(p\_0\) for zero distance as function
        of the decay strengh and the target connectivity.
        The connectivity interpolates \(p\_0\) linearly between
        the target connectivity and 1, according to `decay_strength`.

        Parameters
        :   - **decay** (*float in* *[**0**,**1**]*) – How fast the connection probability should decay with distance.
              A value of 1 indicates fastest decay, 0 indicates no decay et all.
            - **target\_connectivity** (*float in* *[**0**,* *1**]*) – Desired overall target connectivity.

        Returns
        :   **p\_0** – Connectivity for zero distance.

        Return type
        :   float in [0, 1]

*class* `connectome.model.ll.``LL`(*\*\*kwargs*)¶
:   **Inputs:** nr\_exc\_subpopulations, reciprocity\_exc, p\_inh, p\_exc, nr\_neurons, inh\_ratio

    Layered recurrent network.

    It has the additional parameters

    Parameters
    :   - **nr\_exc\_subpopulations** – int
          Number of layers. Only the excitatory neurons are layered.
        - **reciprocity\_exc** – float in [0, 1]
          Excitatory reciprocity of the network.
          Note that the connectivity and the number of layers constrain the reciprocity.

*class* `connectome.model.syn.``SYN`(*\*\*kwargs*)¶
:   **Inputs:** pool\_size, p\_inh, p\_exc, nr\_neurons, inh\_ratio

    Recurrently embedded synfire chain.

    It has the additional parameters

    Parameters
    :   **pool\_size** – int
        Size of the excitatory pools.

*class* `connectome.model.api.``API`(*\*\*kwargs*)¶
:   **Inputs:** n\_pow, feature\_space\_dimension, p\_inh, p\_exc, nr\_neurons, inh\_ratio

    The antiphase inhibition network.

    It has the additional parameters

    Parameters
    :   - **n\_pow** – float
          Determines how correlations are converted to connection probabilities.
        - **feature\_space\_dimension** – int
          Dimension of the underlying feature space.

    `signed_to_positive_correlations`(*signed\_correlations*)¶
    :   Modify according to
        (correlation + 1) / 2.

*class* `connectome.model.sorn.``SORN`(*\*\*kwargs*)¶
:   **Inputs:** eta\_stdp, eta\_intrinsic, nr\_patterns, p\_inh, p\_exc, nr\_neurons, inh\_ratio

    Self-organizing recurrent neural network.

    It has the additional parameters

    Parameters
    :   - **eta\_stdp** – float
          Learning rate for STDP.
        - **eta\_intrinsic** – float
          Learning rate for intrinsic plasticity.
        - **nr\_patterns** – int
          For nr\_patterns == -1, the number of patterns is infinite.

    Notes

    This implementation is efficiently realized in Cython and parallelized with OpenMP.

*class* `connectome.model.fever.``ERFEVER`(*\*\*kwargs*)¶
:   **Inputs:** feverization\_ratio, feature\_space\_dimension, p\_inh, p\_exc, nr\_neurons, inh\_ratio

    FEVER network.

    Apart of the standard parameters of each `network model`
    it has the following additional parameters:

    Parameters
    :   - **feature\_space\_dimension** – int
          Dimension of the underlying feature space.
        - **feverization\_ratio** – float in the inverval [0,1]
          Determines to which degree the network interpolates
          between ER and FEVER. A value of feverization\_ratio=0
          produces an ER graph. A value of 1, however, does *not*
          produce the original FEVER model. Instead, it produces
          a maximally feverized network, as determined by the
          connectivity and feature space dimension.

    `FEVERInitialConnectivity`¶
    :   alias of `connectome.model.fever.ERFEVERInitialConnectivity`

# discriminatEM

### Navigation

- Installation
- Model selection from the command line with discriminatEM
- Quickstart
- The connectome package
- License

- Connectome models
- Connectome analysis
- Connectome noise
- Network shuffling
- Path enumeration sampling
- Connectome builder
- Connectome function
- Connectome ABC Tasks
- ABC-SMC
- Parallel job execution
- RNN

### Related Topics

- Documentation overview
  - Previous: License
  - Next: Connectome analysis

### Quick search

©2017, Emmanuel Klinger, Carsten Marr, Fabian J. Theis, Moritz Helmstaedter.
|
Powered by Sphinx 3.5.4
& Alabaster 0.7.12
