# Supplementary material for: Cellular connectomes as arbiters of local circuit models in the cerebral cortex
Source: Nat Commun. 2021 May 13;12:2785. doi: 10.1038/s41467-021-22856-z (PMC8119988; doi:10.1038/s41467-021-22856-z)
Supplement: Supplementary file 3 — Source Data [file 41467_2021_22856_MOESM3_ESM.zip › doc/connectome_pes.html]

Path enumeration sampling — discriminatEM documentation

# Path enumeration sampling¶

`connectome.pes.strong_path_enumeration_sampling.``pes`(*arr*, *int max\_length*, *probabilities=None*, *repeat=1*, *ratio\_paths\_to\_nodes=1*)¶
:   Sample strong edge paths uniformly using the path enumeration sampling algorithm 1.

    Parameters
    :   - **arr** (*2d array*) – Directed adjacency matrix.
        - **max\_length** (*int*) – Sample paths up to length max\_length.
        - **probabilities** (*1d array of length max\_length*) – Probabilities[n] is the probability of
          exploring a path of length n further.
        - **repeat** (*int*) – Repeat the builder procedure `repeat` times and sum up the so
          obtained paths/cycles.
        - **ratio\_paths\_to\_nodes** (*float*) – Try to sample ratio\_paths\_to\_nodes \* nr\_nodes paths of length max\_length.

    Returns
    :   - **paths** (*list*) –

          List of nr of paths of given length:

          > - paths[0] = Nr of nodes
          > - paths[1] = Nr of paths with 1 edge
          > - paths[2] = Nr of paths consisting of 2 edges
          > - etc. ….
        - **cycles** (*list*) – List of nr of cycles of given length.

    Note

    The algorithm is rather unstable for paths of length larger than about 50;
    in these cases the number of returned paths can both exponentially increase or decay
    (i.e. no path is sampled).
    For shorter paths (length shorter than about 20), this algorithm is stable.
    Importantly, in this algorithm the paths are sampled uniformly, unlike in
    random-walk based sampling algorithms which do not yield uniform samples.

    1
    :   Wernicke, Sebastian. “Efficient Detection of Network Motifs.”
        IEEE/ACM Trans. Comput. Biol. Bioinformatics 3, no. 4 (October 2006):
        347–359. doi:10.1109/TCBB.2006.51.

`connectome.pes.weak_path_enumeration_sampling.``weak_edge_pes`(*arr*, *max\_length\_py=None*, *probabilities\_py=None*, *repeat=1*, *ratio\_paths\_to\_nodes=1*)¶
:   Sample weak edge paths uniformly.

    Parameters
    :   - **arr** (*2d array*) – Directed adjacency matrix.
        - **max\_length** (*int*) – Sample paths up to length `max_length`.
        - **probabilities\_py** (*1d array*) – Probabilities[k] is the probability with which to extend a path
          of length k further.
        - **repeat** (*int*) – Number of times to repeat the building process.

    Returns
    :   **result\_list** – List of counts of how many paths, balanced/unbalanced cycles
        of each length were found,

        > - result\_list[k,0] : no cycles
        > - result\_list[k,1] : balanced cycles
        > - result\_list[k,2] : unbalanced cycles

        Here, k is the number of edges in the path.
        Note that for k=0 all entries are always 0.

    Return type
    :   array

# discriminatEM

### Navigation

- Installation
- Model selection from the command line with discriminatEM
- Quickstart
- The connectome package
- License

- Connectome models
- Connectome analysis
- Connectome noise
- Network shuffling
- Path enumeration sampling
- Connectome builder
- Connectome function
- Connectome ABC Tasks
- ABC-SMC
- Parallel job execution
- RNN

### Related Topics

- Documentation overview
  - Previous: Network shuffling
  - Next: Connectome builder

### Quick search

©2017, Emmanuel Klinger, Carsten Marr, Fabian J. Theis, Moritz Helmstaedter.
|
Powered by Sphinx 3.5.4
& Alabaster 0.7.12
