# Supplementary material for: Cellular connectomes as arbiters of local circuit models in the cerebral cortex
Source: Nat Commun. 2021 May 13;12:2785. doi: 10.1038/s41467-021-22856-z (PMC8119988; doi:10.1038/s41467-021-22856-z)
Supplement: Supplementary file 3 — Source Data [file 41467_2021_22856_MOESM3_ESM.zip › doc/py-modindex.html]

Python Module Index — discriminatEM documentation

# Python Module Index

**a** |
**c** |
**p**

|  |  |  |
| --- | --- | --- |
|  |  |  |
|  | **a** |  |
|  | `abcsmc` |  |
|  |  |  |
|  | **c** |  |
|  | `connectome` |  |
|  | `connectome.function` |  |
|  | `connectome.function.criterion` |  |
|  | `connectome.noise` |  |
|  |  |  |
|  | **p** |  |
|  | `parallel` |  |

# discriminatEM

### Navigation

- Installation
- Model selection from the command line with discriminatEM
- Quickstart
- The connectome package
- License

- Connectome models
- Connectome analysis
- Connectome noise
- Network shuffling
- Path enumeration sampling
- Connectome builder
- Connectome function
- Connectome ABC Tasks
- ABC-SMC
- Parallel job execution
- RNN

### Related Topics

- Documentation overview

### Quick search

©2017, Emmanuel Klinger, Carsten Marr, Fabian J. Theis, Moritz Helmstaedter.
|
Powered by Sphinx 3.5.4
& Alabaster 0.7.12
