# Supplementary material for: Cellular connectomes as arbiters of local circuit models in the cerebral cortex
Source: Nat Commun. 2021 May 13;12:2785. doi: 10.1038/s41467-021-22856-z (PMC8119988; doi:10.1038/s41467-021-22856-z)
Supplement: Supplementary file 3 — Source Data [file 41467_2021_22856_MOESM3_ESM.zip › doc/connectome_function_example.html]

<no title> — discriminatEM documentation

For example, to examine how well activity is propagated by a certain SYNIFRE
configuration import:

```
from connectome.function import TestSuite, GreaterThan, PropagationTask
from connectome.model import SYN
```

Tests are defined as follows:

```
syn_test = TestSuite(PropagationTask())
syn_test.add_criterion("fraction_pools_activated", GreaterThan(.5))
res = syn_test.test_model(SYN(nr_neurons=2000, inh_ratio=.1, p_exc=.15, p_inh=.5, pool_size=100))
```

In detail:
A test suite for the propagation task is created:

```
syn_test = TestSuite(PropagationTask())
syn_test
```

```
<TestSuite task=PropagationTask(spike_detection_pool_fraction=0.5, run_time=100. * msecond), criteria={}>
```

The “GreaterThan” criterion is used to verify that a certain fraction of pools is activated:

```
syn_test.add_criterion("fraction_pools_activated", GreaterThan(.5))
syn_test
```

```
<TestSuite task=PropagationTask(spike_detection_pool_fraction=0.5, run_time=100. * msecond), criteria={'fraction_pools_activated': GreaterThan(0.5)}>
```

A model is instantiated:

```
syn = SYN(nr_neurons=2000, inh_ratio=.1, p_exc=.15, p_inh=.5, pool_size=100)
syn
```

```
<SYN nr_neurons=2000, p_exc=0.15, inh_ratio=0.1, pool_size=100, p_inh=0.5>
```

and then tested

```
res = syn_test.test_model(syn)
res["fraction_pools_activated"]
```

```
TestResult(criterion=GreaterThan(0.5), score=1.0, passed=True)
```

The “score” output indicates the fraction of the activated pools and it is
displayed whether the test is passed or not.
In this example, all pools were activated.

# discriminatEM

### Navigation

- Installation
- Model selection from the command line with discriminatEM
- Quickstart
- The connectome package
- License

- Connectome models
- Connectome analysis
- Connectome noise
- Network shuffling
- Path enumeration sampling
- Connectome builder
- Connectome function
- Connectome ABC Tasks
- ABC-SMC
- Parallel job execution
- RNN

### Related Topics

- Documentation overview

### Quick search

©2017, Emmanuel Klinger, Carsten Marr, Fabian J. Theis, Moritz Helmstaedter.
|
Powered by Sphinx 3.5.4
& Alabaster 0.7.12
