# Supplementary material for: Cellular connectomes as arbiters of local circuit models in the cerebral cortex
Source: Nat Commun. 2021 May 13;12:2785. doi: 10.1038/s41467-021-22856-z (PMC8119988; doi:10.1038/s41467-021-22856-z)
Supplement: Supplementary file 3 — Source Data [file 41467_2021_22856_MOESM3_ESM.zip › doc/connectome_function.html]

Connectome function — discriminatEM documentation

# Connectome function¶

Functional model testing.

## Tasks¶

*class* `connectome.function.modeltest.``Task`¶
:   Abstract base class for tasks.

    All tasks should derive from this class.

    *abstract* `__call__`(*model*) → dict¶
    :   Parameters
        :   **model** (*Node*) –

            A model instance of ER, EXP, SORN, LL, API, ERFEVER or SYN.
            Please note the following correspondences between the class names used in this
            code package and the model acronyms used in the manuscript (class name: acronym):

            - ER: ER-ESN,
            - EXP: EXP-LSM,
            - LL: LAYERED,
            - SYN: SYNFIRE,
            - ERFEVER: FEVER,
            - API: API,
            - SORN: STDP-SORN.

        Returns
        :   **task\_result** – Dictionary of task results.

        Return type
        :   dict

*class* `connectome.function.task.memory.``MemoryTask`(*simulation\_time=20*)¶
:   Short term memory task.
    Test the short term memory property of a network.

    Accepts network models with feature vectors.
    E.g., API and FEVER are accepted.

    The task representation is obtained as the sum over the neurons’ feature
    vectors weighted by their activity.

    Parameters
    :   **simulation\_time** (*float*) – Simulation time relative to the neuronal time constant,

*class* `connectome.function.task.texture.``TextureTask`(*nb\_epoch=6000*, *nr\_samples=10000*, *nr\_classes=7*, *length=500*, *verbose=0*, *learning\_rate=<function default\_learning\_rate>*, *slice\_last=250*)¶
:   Texture discrimination task.

    Can be run with any network model.

    The reported accuracy is the accuracy of each output time step considered as individual test point.

    Parameters
    :   - **nb\_epoch** (*int*) – Number epochs for training.
        - **nr\_samples** (*int*) – Total number of samples for the task.
        - **nr\_classes** (*int*) – Number of texture classes sampled from natural images.
        - **length** (*int*) – Length of a texture sequence.
        - **verbose** (*int*) – How much information to print during training.
        - **learning\_rate** (*callable*) – Learning\_rate(nr\_exc\_layers) should return the learning rate
          for a model with nr\_exc\_layers excitatory layers.

    `get_data`()¶
    :   Returns
        :   **X, y** – Data, one-hot-encoded target.

        Return type
        :   tuple of arrays

*class* `connectome.function.task.tuning.``TuningTask`(*return\_arrays=False*)¶
:   Stimulus tuning task.

    Checks if the cortical tuning is sharper than the thalamic input.

    Accepts network models with feature vectors.
    E.g. FEVER and API are accepted.

*class* `connectome.function.task.unsynchronized.``UnsynchronizedActivityTask`¶
:   Accepts the SORN model.

    Checks if the activity of the network is synchronized or not.

*class* `connectome.function.task.propagation.task.``PropagationTask`(*run\_time=100.0 \* msecond*, *spike\_detection\_pool\_fraction: float = 0.5*, *\*\*dyn\_syn\_pars*)¶
:   Checks the propagation of activity along a synfire chain.

    Accepts the SYN.

    Parameters
    :   - **run\_time** (*brian2.Quantity*) – Simulation time.
        - **spike\_detection\_pool\_fraction** (*float in* *[**0**,* *1**]*) – Fraction of neurons in a pool which have to spike
          to detect event as pool activation.

## Criteria¶

*class* `connectome.function.modeltest.``Criterion`¶
:   Abstract Criterion base class.
    All criteria should implement the interface defined in this ABC.

    *abstract* `__call__`(*value: Union[float, bool]*) → bool¶
    :   Parameters
        :   **value** (*float* *or* *bool*) – The value to be evaluated.

        Returns
        :   **passed** – Returns true if the criterion was satisfied.

        Return type
        :   bool

*class* `connectome.function.criterion.``GreaterThan`(*lower*)¶
:   Greater than criterion.

    True if the test quantity is greater than lower.

    Parameters
    :   **lower** (*float*) – Quantity has to be strictly greater than lower to yield “true”.

*class* `connectome.function.criterion.``LessThan`(*upper*)¶
:   Greater than criterion.

    True if the test quantity is less then upper.

    Parameters
    :   **upper** (*float*) – Quantity has to be strictly less then upper to yield true.

*class* `connectome.function.criterion.``Range`(*lower*, *upper*)¶
:   Range criterion.

    True if the test quantity is within the range [lower, upper].

    Parameters
    :   - **lower** (*float*) – Lower boundary of range.
        - **upper** (*float*) – Upper boundary of range.

*class* `connectome.function.criterion.``TrueCriterion`¶
:   Return True if the test quantity itself is True.

## Test runners¶

*class* `connectome.function.modeltest.``TestSuite`(*task: connectome.function.modeltest.Task*)¶
:   Test suite for functional checking.

    Parameters
    :   **task** (*Task*) – The task for this test suite.

    `add_criterion`(*criterion\_name: str*, *criterion: connectome.function.modeltest.Criterion*)¶
    :   Add a test criterion.

        Parameters
        :   - **criterion\_name** (*str*) – Name of the quantity to be tested.
              This has to correspond to one of the output keys of the task.
            - **criterion** (*Criterion*) – The test criteria.

    `enqueue_model`(*model*)¶
    :   Enqueue a model to be tested, but do not execute the tests.

        Parameters
        :   **model** (*Model*) – Can be run by the model\_runner.
            Usually an instance of one of the network models.

    `execute_enqueued_models`(*mapper=None*) → connectome.function.modeltest.TestSuiteResultList¶
    :   Execute the test task on all enqueued models.

        Parameters
        :   **mapper** (*map-like object*) – Can be distributed. Has to work like a map.

        Returns
        :   **test\_suite\_result\_list** – List of results of the test.

        Return type
        :   TestSuiteResultList

    `mapper`¶
    :   alias of `map`

    `test_model`(*model*) → connectome.function.modeltest.TestSuiteResult¶
    :   Test a single model.

        Parameters
        :   **model** (*Model*) – Can be run by the model\_runner.
            Usually an instance of one of the network models.

## Test results¶

*class* `connectome.function.modeltest.``TestSuiteResultList`(*results: List[connectome.function.modeltest.TestSuiteResult]*)¶
:   Represents a list of test suite results.

    Parameters
    :   **results** (*List**[**TestSuiteResult**]*) – A list of `test suite results`.

    `append`(*item: connectome.function.modeltest.TestSuiteResult*)¶
    :   Parameters
        :   **item** (*TestSuiteResult*) – Append item to the list.

    `done`()¶
    :   Check if the tests have executed already.
        This is meant to work with the Future protocol as in the concurrent.futures module.

        Returns
        :   **done** – True if done, false otherwise.

        Return type
        :   bool

    *property* `passed`¶
    :   True if all tests were passed.

    *property* `results_list`¶
    :   Transforms the possibly passed iterator into a list on first call and returns it.

    `wait`()¶
    :   Wait for all tasks to be executed and return then.

*class* `connectome.function.modeltest.``TestSuiteResult`(*model: str*)¶
:   Result of a single test.

    Parameters
    :   **model** (*Node*) – The evaluated model.

    `add`(*evaluation: connectome.function.modeltest.TestResult*)¶
    :   Add a single test result of the evaluation of a single criterion to the test suite result.

        Parameters
        :   **evaluation** (*TestResult*) – Result of a single test.

    *property* `passed`¶
    :   True if all criteria were passed.

*class* `connectome.function.modeltest.``TestResult`(*criterion\_name: str*, *criterion: Optional[connectome.function.modeltest.Criterion]*, *score: Union[float, KeyError]*, *passed: bool*)¶
:   Result of the evaluation of a single `criterion`.

    Parameters
    :   - **criterion\_name** (*str*) – Name of the checked criterion.
        - **criterion** (*Union**[**Criterion**,* *None**]*) – The checked criterion.
        - **score** (*float*) – The score of the criterion.

# discriminatEM

### Navigation

- Installation
- Model selection from the command line with discriminatEM
- Quickstart
- The connectome package
- License

- Connectome models
- Connectome analysis
- Connectome noise
- Network shuffling
- Path enumeration sampling
- Connectome builder
- Connectome function
  - Tasks
  - Criteria
  - Test runners
  - Test results
- Connectome ABC Tasks
- ABC-SMC
- Parallel job execution
- RNN

### Related Topics

- Documentation overview
  - Previous: Connectome builder
  - Next: Connectome ABC Tasks

### Quick search

©2017, Emmanuel Klinger, Carsten Marr, Fabian J. Theis, Moritz Helmstaedter.
|
Powered by Sphinx 3.5.4
& Alabaster 0.7.12
