# Supplementary material for: Cellular connectomes as arbiters of local circuit models in the cerebral cortex
Source: Nat Commun. 2021 May 13;12:2785. doi: 10.1038/s41467-021-22856-z (PMC8119988; doi:10.1038/s41467-021-22856-z)
Supplement: Supplementary file 3 — Source Data [file 41467_2021_22856_MOESM3_ESM.zip › doc/connectome.html]

The connectome package — discriminatEM documentation

# The connectome package¶

In the following the different parts of software package are described in more
depth than in the quick start tutorial.
These parts include network generation, network analysis, noise models,
model composition and pipeline generation, functional model checking and
ABC inference.

## Creating networks - Sampling from stochastic network models¶

Network models are defined within the connectome.model package.
For example, the FEVER model is simulated with:

```
from connectome.model import FEVER
fever = FEVER(nr_neurons=2000, inh_ratio=.2, feverization_ratio=.5,
              feature_space_dimension=100)
network = fever(p_exc=.2, p_inh=.5)
```

or alternatively:

```
fever = FEVER(nr_neurons=2000, inh_tatio=.2, p_exc=.2, p_inh=.5,
              feverization_ratio=.5, feature_space_dimension=100)
network = fever()
```

Any argument can be either passed at object initialization and is then stored or can be passed when the object is called.
Any argument provided once is stored and can be omitted on subsequent calls if no change is required.

The provided models are

- `Erdős–Rényi - echo state network (ER-ESN)`
- `Exponentially decaying connectivity - liquid state machine (EXP-LSM)`
- `Layered network (LAYERED)`
- `Synfire chain (SYNFIRE)`
- `Feature vector recombination network (FEVER)`
- `Antiphase inhibition (API)`
- `Spike timing dependent plasticity - self-organizing recurrent neural network (STDP-SORN)`

Please note the following correspondences between the class names used in this
code package and the model acronyms used in the manuscript (class name ➜ acronym):

- ER ➜ ER-ESN,
- EXP ➜ EXP-LSM,
- LL ➜ LAYERED,
- SYN ➜ SYNFIRE,
- ERFEVER ➜ FEVER,
- API ➜ API,
- SORN ➜ STDP-SORN.

## Networks¶

A network sample is represented by an instance
of the `Network` class.
This class represents a biological neural network and is a thin wrapper around
a Numpy 2D array with additional methods and properties relevant
for biological neural networks. These properties include the number
of excitatory neurons Network.nr\_exc and the number of inhibitory neurons Network.nr\_inh.
It also supports indexing in terms of cell type. For example:

```
network["E", "I"]
```

returns a view on the block of the matrix projecting from the excitatory population to the inhibitory population.
In addition, this class provides methods for relabeling and removing neurons.
Details are found in the API documentation: `Network`.

## Network analysis¶

The connectome.analysis module implements functionality for the analysis of connectomes.
All the analysis classes accept a single input called network and return a dictionary.
For example, to calculate the excitatory connectivity, execute:

```
from connectome.model import ER
from connectome.analysis import ConnectivityEstimator

er = ER(nr_exc=2000, inh_ratio=.1, p_exc=.2, p_inh=.5)
network = er()
connectivity_estimator = ConnectivityEstimator()
analysis_result = connectivity_estimator(network=network)
excitatory_connectivity = analysis_result["p_ee"]
```

## Noise in networks¶

Noise models are defined in the connectome.noise package. Noise objects modify networks. For example:

```
from connectome.model import LL
from connectome.noise import Subsampling

network = LL(nr_neurons=2000, inh_ratio=.1, p_exc=.2, p_inh=.5, reciprocity_exc=.2)()
noisy_network = Subsmapling(network=network, subsampling_fraction=.7)()
```

Produces a network noisy\_network in which 30% of the original neurons are randomly removed.
This network can be analyzed or further noise can be applied to it.

## Monte Carlo sampling based analysis¶

The connectome.pes package provides efficient Cython implementations of path sampling algorithms. Weak path enumeration sampling,
provided by `weak_edge_pes` does not take into account
the direction of connections. Strong path enumeration sampling,
provided by `pes`, takes the direction of edges into account.

Path sampling provides an alternative way to quantify the number of cycles in a network. The cycles analysis implemented in
`RelativeCycleAnalysis` is a measure of dynamic cycles:
an edge can occur in a dynamic cycle multiple times. The path sampling algorithms measure static cycles in which each
edge occurs at most once.

## Building composite models¶

A composite model can be build as pipeline of different processing steps:

1. network sampling,
2. noise application,
3. network analysis.

Functionality for the assembly of such pipelines is implemented in the
connectome.builder module.

### Definition of models pipelines with priors, noise and analysis¶

The necessary parts to define a pipeline can be stored in a JSON file.
The first level is structured into three parts: “models”, “noise” and “analysis”:

```
{
   "models": ...
   "noise": ...
   "analysis": ...
}
```

The “models” section is further subdivided into “joint\_parameters” and “model\_specific\_parameters”.
The “joint\_parameters” are the ones shared by all models, the “model\_specific\_parameters” are the ones
which apply only to a specific model. The “model\_specific\_parameters” contain one subsection for each model –
if that model has additional parameters or not.
In these subsections, the prior distributions can be defined (if applicable). There
are two ways to define a prior:

1. a fixed delta point prior
2. a distribution

A delta point prior is defined by providing the point as number:

```
{
  "pool_size": 80
 }
```

The above defines the parameter “pool\_size” to be fixed at 80.
A distribution is defined by indicating the distribution name and its shape parameters:

```
{
 "pool_size": {
   "type": "randint",
   "args": [
     80,
     300
   ]
 }
```

The above defines “pool\_size” to be uniformly distributed on the set \(\{80, 81, 82, \ldots, 298, 299\}\) 1.
Distributions from the scipy.stats package are supported. The “type” determines the distribution, “args” its arguments.
Additionally, a “kwargs” key might also be present. The content is then passed as keyword argument to the scipy.stats
distribution. The definition of noise is analogous to the definition of models.

A number of analysis classes can be defined to produce the output of the pipeline. Every class which occurs
in the analysis section produces its output. These are then combined. A full example of a model definitions JSON
file could look like the one below:

```
{
  "models": {
    "joint_parameters": {
      "nr_neurons": 2000,
      "inh_ratio": 0.1,
      "p_exc": 0.2,
      "p_inh": 0.6
    },
    "model_specific_parameters": {
      "ER": {
      },
      "LL": {
        "nr_exc_subpopulations": {
          "type": "randint",
          "args": [
            2,
            5
          ]
        },
        "reciprocity_exc": {
          "type": "uniform",
          "args": [
            0.17,
            0.06
          ]
        }
      },
      "SYN": {
        "pool_size": {
          "type": "randint",
          "args": [
            80,
            300
          ]
        }
      },
      "API": {
        "n_pow": {
          "type": "uniform",
          "args": [
            4,
            2
          ]
        },
        "feature_space_dimension": {
          "type": "randint",
          "args": [
            5,
            50
          ]
        }
      },
      "EXP": {
        "decay": 1
      },
      "ERFEVER": {
        "feature_space_dimension": {
          "type": "randint",
          "args": [
            20,
            200
          ]
        },
        "feverization_ratio": {
          "type": "uniform",
          "args": [
            0.5,
            0.4
          ]
        }
      },
      "SORN":{
        "nr_patterns": -1,
        "eta_stdp": {
          "type": "uniform",
          "args": [
            0.0006,
            0.0008
          ]
        },
        "eta_intrinsic": {
          "type": "uniform",
          "args": [
            0.05,
            0.05
          ]
        }
      }
    }
  },
  "noise": {
    "type": "RemoveAddNoiseAndSubsample",
    "parameters": {
      "fraction_remove_and_add": {
          "type": "beta",
          "args": [
            2,
            10
          ]
        },
      "subsampling_fraction": 1
    }
  },
  "analysis": {
    "NrNeurons": {
    },
    "ConnectivityEstimator": {
    },
    "RelativeCycleAnalysis": {
      "length": 5
    },
    "ReciprocityEstimator":{
    },
    "RelativeReciprocityEstimator": {
    },
    "InOutDegreeCorrelation":{
    }
  }
}
```

### Building models and sampling using model definitions¶

Having defined the priors, the noise and the analysis tasks the connectome.builder package can be used
to sample and analyse models (see Connectome builder.).
An example script is provided below.

```
import argparse
import json

from connectome.builder import Sampler, DistributedFileStorer, PipelineBuilder, TimedModelWithPriorSample
from parallel import SGE, sge_available

parser = argparse.ArgumentParser(description='Sample networks.')
parser.add_argument('model_definitions', type=str, help='Model definition json')
parser.add_argument('nr_samples_per_model', type=int, help='Nr samples per model')
parser.add_argument('destination', type=str, help='Destination db')
parser.add_argument("--num_threads", type=int, default=1, help="Number of threads")
parser.add_argument("--priority", type=int, default=0, help="Number of threads")
args = parser.parse_args()


with open(args.model_definitions) as f:
    pars = json.load(f)
    modelbuilder = PipelineBuilder(pars)


def make_sample_job(name):
    model = modelbuilder.build_pipeline(name)
    prior = modelbuilder.build_prior(name)
    model_with_prior = TimedModelWithPriorSample(prior, model, name=name)
    return model_with_prior


map_engine = SGE(num_threads=args.num_threads, priority=args.priority, name="sample").map if sge_available() else map
sampler = Sampler(map_engine, DistributedFileStorer(args.destination, timeout=120))
for name in modelbuilder.defined_models():
    sampler.add_model(make_sample_job(name))
sampler.sample(args.nr_samples_per_model)
```

## Functional model testing¶

Classes for functional model testing are implemented in the `connectome.function` package (see Connectome function).

For example, to examine how well activity is propagated by a certain SYNIFRE
configuration import:

```
from connectome.function import TestSuite, GreaterThan, PropagationTask
from connectome.model import SYN
```

Tests are defined as follows:

```
syn_test = TestSuite(PropagationTask())
syn_test.add_criterion("fraction_pools_activated", GreaterThan(.5))
res = syn_test.test_model(SYN(nr_neurons=2000, inh_ratio=.1, p_exc=.15, p_inh=.5, pool_size=100))
```

In detail:
A test suite for the propagation task is created:

```
syn_test = TestSuite(PropagationTask())
syn_test
```

```
<TestSuite task=PropagationTask(spike_detection_pool_fraction=0.5, run_time=100. * msecond), criteria={}>
```

The “GreaterThan” criterion is used to verify that a certain fraction of pools is activated:

```
syn_test.add_criterion("fraction_pools_activated", GreaterThan(.5))
syn_test
```

```
<TestSuite task=PropagationTask(spike_detection_pool_fraction=0.5, run_time=100. * msecond), criteria={'fraction_pools_activated': GreaterThan(0.5)}>
```

A model is instantiated:

```
syn = SYN(nr_neurons=2000, inh_ratio=.1, p_exc=.15, p_inh=.5, pool_size=100)
syn
```

```
<SYN nr_neurons=2000, p_exc=0.15, inh_ratio=0.1, pool_size=100, p_inh=0.5>
```

and then tested

```
res = syn_test.test_model(syn)
res["fraction_pools_activated"]
```

```
TestResult(criterion=GreaterThan(0.5), score=1.0, passed=True)
```

The “score” output indicates the fraction of the activated pools and it is
displayed whether the test is passed or not.
In this example, all pools were activated.

It is also possible to test many models simultaneously, possibly on a distributed execution engine.
This can be achieved using the `enqueue_model` and `execute_enqueued_models` methods of the
`TestSuite`.
Additionally, the `TestSuite.mapper` class attribute can be set for distributed execution or a mapper can be passed to
`execute_enqueued_models`.

1
:   The last number is not included.

## ABC with network models¶

For ABC tasks with networks, the
Facade class `ABCConnectomeTask` is provided and recommended.
This class encapsulates inference tasks.
A list of such tasks can be executed with the `execute_task_list`
function.

# discriminatEM

### Navigation

- Installation
- Model selection from the command line with discriminatEM
- Quickstart
- The connectome package
  - Creating networks - Sampling from stochastic network models
  - Networks
  - Network analysis
  - Noise in networks
  - Monte Carlo sampling based analysis
  - Building composite models
  - Functional model testing
  - ABC with network models
- License

- Connectome models
- Connectome analysis
- Connectome noise
- Network shuffling
- Path enumeration sampling
- Connectome builder
- Connectome function
- Connectome ABC Tasks
- ABC-SMC
- Parallel job execution
- RNN

### Related Topics

- Documentation overview
  - Previous: Quickstart
  - Next: License

### Quick search

©2017, Emmanuel Klinger, Carsten Marr, Fabian J. Theis, Moritz Helmstaedter.
|
Powered by Sphinx 3.5.4
& Alabaster 0.7.12
