# Supplementary material for: Cellular connectomes as arbiters of local circuit models in the cerebral cortex
Source: Nat Commun. 2021 May 13;12:2785. doi: 10.1038/s41467-021-22856-z (PMC8119988; doi:10.1038/s41467-021-22856-z)
Supplement: Supplementary file 3 — Source Data [file 41467_2021_22856_MOESM3_ESM.zip › doc/connectome_analysis.html]

Connectome analysis — discriminatEM documentation

# Connectome analysis¶

This package provides connectome analysis routines.

*class* `connectome.analysis.connectivity.``ConnectivityEstimator`(*\*\*kwargs*)¶
:   **Inputs:** network, measures

    Estimate the connectivity of a network.

    Output Keys: p\_ee, p\_ii, p\_ie, p\_ei

*class* `connectome.analysis.inoutdegreecorrelation.``InOutDegreeCorrelation`(*\*\*kwargs*)¶
:   **Inputs:** network, measures

    Estimates the correlation of in and out degrees of the excitatory subpopulation.

    Output keys: in\_out\_degree\_correlation\_exc

*class* `connectome.analysis.relativecycleanalysis.``RelativeCycleAnalysis`(*\*\*kwargs*)¶
:   **Inputs:** length, network, measures

    Compares relative to ER the expected number of cycles which is approximated here
    by \((p\*n)^{length}\), where `p` is the connectivity, `n` the number of excitatory nodes
    and `length` the cycle length.

    Note that this approximation ignores the non-independent nature of the Bernoulli
    product on the diagonal of the matrix power. This approximation fails for very small
    and very sparse networks but is precise for large and dense networks.

    Configuration parameter: length, integer

    Output keys: relative\_cycles\_<length>

*class* `connectome.analysis.reciprocity.``ReciprocityEstimator`(*\*\*kwargs*)¶
:   **Inputs:** network, measures

    Estimate the network’s reciprocities; here “reciprocity\_ei” means:
    :   Given a connection from E -> I, what is the probability
        of the reciprocated connection to also exist?

    Output keys: reciprocity\_ee, reciprocity\_ii, reciprocity\_ei, reciprocity\_ie

*class* `connectome.analysis.relativereciprocity.``RelativeReciprocityEstimator`(*\*\*kwargs*)¶
:   **Inputs:** network, measures

    Estimate the reciprocity relative to an ER network of the same connectivity.
    See also `ReciprocityEstimator`.

    Output keys: relative\_reciprocity\_ee, relative\_reciprocity\_ei, relative\_reciprocity\_ie, relative\_reciprocity\_ii

# discriminatEM

### Navigation

- Installation
- Model selection from the command line with discriminatEM
- Quickstart
- The connectome package
- License

- Connectome models
- Connectome analysis
- Connectome noise
- Network shuffling
- Path enumeration sampling
- Connectome builder
- Connectome function
- Connectome ABC Tasks
- ABC-SMC
- Parallel job execution
- RNN

### Related Topics

- Documentation overview
  - Previous: Connectome models
  - Next: Connectome noise

### Quick search

©2017, Emmanuel Klinger, Carsten Marr, Fabian J. Theis, Moritz Helmstaedter.
|
Powered by Sphinx 3.5.4
& Alabaster 0.7.12
