# Supplementary material for: Cellular connectomes as arbiters of local circuit models in the cerebral cortex
Source: Nat Commun. 2021 May 13;12:2785. doi: 10.1038/s41467-021-22856-z (PMC8119988; doi:10.1038/s41467-021-22856-z)
Supplement: Supplementary file 3 — Source Data [file 41467_2021_22856_MOESM3_ESM.zip › doc/connectome_noise.html]

Connectome noise — discriminatEM documentation

# Connectome noise¶

This package implements connectome noise models.

*class* `connectome.noise.combined_noise.``RemoveAddNoiseAndSubsample`(*\*\*kwargs*)¶
:   **Inputs:** network, fraction\_remove\_and\_add, subsampling\_fraction

    Subsample a fraction of the network and shuffle edges in the remaining portion.

    Parameters
    :   - **network** (`Network`) – The network.
        - **fraction\_remove\_and\_add** (*float in* *[**0**,* *1**]*) – Fraction of edges to be removed and randomly reinserted again.
          The sign of an edge is automatically converted to match the type
          of the new presynaptic neuron.
        - **subsampling\_fraction** (*float in* *[**0**,* *1**]*) – Fraction of the network to keep.
          subsampling\_fraction=1 implies the full network.

    `construct_graph`()¶
    :   Construct the computational graph here.

*class* `connectome.noise.barrelcutnoise.``BarrelCutNoise`(*\*\*kwargs*)¶
:   **Inputs:** f, p, network

    Simulate to cut a barrel into two pieces.
    Also simulate network noise.
    For spatially embedded networks, the embedding is taken into account.

    Parameters
    :   - **f** – float
          Fraction of the network to be removed.
        - **p** – float
          Noise applied to the network after cutting.
          The noise type is `RemoveAndAddEdgesNoise`.
          The parameter p is its parameter fraction\_remove\_and\_add.

    `edge_removal_probability`()¶
    :   Uses the empirical distribution \(emp\) from 1:

        | Number synapses per connection | Probability |
        | --- | --- |
        | 1 | 0 |
        | 2 | 2/11 |
        | 3 | 5 /11 |
        | 4 | 2/11 |
        | 5 | 2/11 |

        The expected value is 3.36.

        Assumes that synapses are uniformly distributed throughout
        the barrel. Therefore, probability of having a synpase on
        the “same” side is \((1-f)\).

        The fraction of edges additionally lost due to the cut is

        \[p\_{cut}(f) = \sum\_{n=0}^5 Binom(0 |n, f)emp(n).\]

        Parameters
        :   **f** (*float*) – Fraction of the barrel which is still left.

        1
        :   Feldmeyer, Dirk, Veronica Egger, Joachim Lübke, and Bert Sakmann.
            “Reliable Synaptic Connections between Pairs of Excitatory Layer
            4 Neurones within a Single ‘barrel’ of Developing Rat Somatosensory Cortex.”
            The Journal of Physiology 521, no. 1 (1999):
            169–90. doi:10.1111/j.1469-7793.1999.00169.x.

*class* `connectome.noise.edgeshufflingnoise.``EdgeShufflingNoise`(*\*\*kwargs*)¶
:   **Inputs:** network, fraction\_draws\_over\_nr\_synapses

    Shuffle edges randomly.

    This is an abstract class implementing a template method pattern.

    Parameters
    :   - **network** (`Network`) – The network to be shuffled.
        - **fraction\_draws\_over\_nr\_synapses** (*int*) – The parameter fraction\_draws\_over\_nr\_synapses multiplied with the number of synapses (edges)
          in the network is the to be rounded number of reshuffling attempts.

    *abstract* `_shuffle_subpopulation`(*submatrix*, *diagonal*)¶
    :   Template method for subclasses.

        Parameters
        :   - **submatrix** (*view on array*) – The part to be reshuffled.
            - **diagonal** (*bool*) – True if it contains the diagonal part of the complete array.

*class* `connectome.noise.inandoutdegreepreservingnoise.``InAndOutDegreePreservingNoise`(*\*\*kwargs*)¶
:   **Inputs:** network, fraction\_draws\_over\_nr\_synapses

    Shuffle edges in a way such that in and out degrees are preserved.

    This is a subclass of `EdgeShufflingNoise`.

    The shuffling is achieved through a Markov-Chain-Monte-Carlo method.
    An ergodic Markov chain on the space of graphs is constructed.
    It is then sampled from this Markov chain.
    The sample is thus an unbiased sample from all graphs with the given in
    and out degree distribution.

*class* `connectome.noise.outdegreepreservingnoise.``OutDegreePreservingNoise`(*\*\*kwargs*)¶
:   **Inputs:** network, fraction\_draws\_over\_nr\_synapses

    Shuffle a network such that all out degrees are preserved.

    This is a subclass of `EdgeShufflingNoise`.

*class* `connectome.noise.removeandaddedgesnoise.``RemoveAndAddEdgesNoise`(*\*\*kwargs*)¶
:   **Inputs:** network, fraction\_remove\_and\_add

    Remove and add edges uniformly.

    Parameters
    :   - **network** (`Network`) – The network to which the noise is applied.
        - **fraction\_remove\_and\_add** (*float in* *[**0**,* *1**]*) – The fraction of edges to be removed and randomly reinserted.

*class* `connectome.noise.subsampling.``Subsampling`(*\*\*kwargs*)¶
:   **Inputs:** subsampling\_fraction, network

    Parameters
    :   - **network** (`Network`) – The network to which the noise is applied.
        - **subsampling\_fraction** (*float in* *[**0**,* *1**]*) – Fraction of neurons to keep.

# discriminatEM

### Navigation

- Installation
- Model selection from the command line with discriminatEM
- Quickstart
- The connectome package
- License

- Connectome models
- Connectome analysis
- Connectome noise
- Network shuffling
- Path enumeration sampling
- Connectome builder
- Connectome function
- Connectome ABC Tasks
- ABC-SMC
- Parallel job execution
- RNN

### Related Topics

- Documentation overview
  - Previous: Connectome analysis
  - Next: Network shuffling

### Quick search

©2017, Emmanuel Klinger, Carsten Marr, Fabian J. Theis, Moritz Helmstaedter.
|
Powered by Sphinx 3.5.4
& Alabaster 0.7.12
