# Supplementary material for: Cellular connectomes as arbiters of local circuit models in the cerebral cortex
Source: Nat Commun. 2021 May 13;12:2785. doi: 10.1038/s41467-021-22856-z (PMC8119988; doi:10.1038/s41467-021-22856-z)
Supplement: Supplementary file 3 — Source Data [file 41467_2021_22856_MOESM3_ESM.zip › doc/license.html]

License — discriminatEM documentation

# License¶

This package is licensed under a MIT license. See the License text below.

## Author¶

This package is written by Emmanuel Klinger (emmanuel.klinger@brain.mpg.de).

## License text¶

```
Copyright (c) 2017 Emmanuel Klinger, Carsten Marr, Fabian J. Theis, Moritz Helmstaedter

This package is part of the manuscript: "Cellular connectomes as arbiters of
local computational models in the cerebral cortex".

Permission is hereby granted, free of charge, to any person obtaining a copy
of this software and associated documentation files (the "Software"), to deal
in the Software without restriction, including without limitation the rights
to use, copy, modify, merge, publish, distribute, sublicense, and/or sell
copies of the Software, and to permit persons to whom the Software is
furnished to do so, subject to the following conditions:

The above copyright notice and this permission notice shall be included in all
copies or substantial portions of the Software.

THE SOFTWARE IS PROVIDED "AS IS", WITHOUT WARRANTY OF ANY KIND, EXPRESS OR
IMPLIED, INCLUDING BUT NOT LIMITED TO THE WARRANTIES OF MERCHANTABILITY,
FITNESS FOR A PARTICULAR PURPOSE AND NONINFRINGEMENT. IN NO EVENT SHALL THE
AUTHORS OR COPYRIGHT HOLDERS BE LIABLE FOR ANY CLAIM, DAMAGES OR OTHER
LIABILITY, WHETHER IN AN ACTION OF CONTRACT, TORT OR OTHERWISE, ARISING FROM,
OUT OF OR IN CONNECTION WITH THE SOFTWARE OR THE USE OR OTHER DEALINGS IN THE
SOFTWARE.
```

# discriminatEM

### Navigation

- Installation
- Model selection from the command line with discriminatEM
- Quickstart
- The connectome package
- License
  - Author
  - License text

- Connectome models
- Connectome analysis
- Connectome noise
- Network shuffling
- Path enumeration sampling
- Connectome builder
- Connectome function
- Connectome ABC Tasks
- ABC-SMC
- Parallel job execution
- RNN

### Related Topics

- Documentation overview
  - Previous: The connectome package
  - Next: Connectome models

### Quick search

©2017, Emmanuel Klinger, Carsten Marr, Fabian J. Theis, Moritz Helmstaedter.
|
Powered by Sphinx 3.5.4
& Alabaster 0.7.12
