# Supplementary material for: Cellular connectomes as arbiters of local circuit models in the cerebral cortex
Source: Nat Commun. 2021 May 13;12:2785. doi: 10.1038/s41467-021-22856-z (PMC8119988; doi:10.1038/s41467-021-22856-z)
Supplement: Supplementary file 3 — Source Data [file 41467_2021_22856_MOESM3_ESM.zip › doc/connectome_shuffling.html]

Network shuffling — discriminatEM documentation

# Network shuffling¶

`connectome.shuffling.uniform_degree_preserving_shuffling.``shuffle_graph`(*adjacency\_matrix*, *bool target\_population\_equals\_source\_population*, *unsigned long long nr\_draws=0*, *bool triangle\_moves=True*, *bool count=False*)¶
:   Shuffles a graph, **not** modifying the original graph but returning a
    new shuffled graph.

    Parameters
    :   - **adjacency\_matrix** (*ndarray*) – Adjacency matrix. Link from i to j is A[i,j].
        - **target\_population\_equals\_source\_population** (*bool*) –

          Whether the target population equals the source population.

          - For connections from exc. to exc. and from inh. to inh. this should be true.
          - For connections from exc. to inh. and from inh. to exc. this should be false.
          - If this argument is set to `True` no entries on the diagonal of
            the adjacency matrix (self-loops) are allowed.
          - If this argument is set to `False` entries on the diagonal of the
            adjacency matrix are allowed.
        - **nr\_draws** (*unsigned long long*) – Number of draws of canonical moves.
        - **triangle\_moves** (*bool*) –
          - True: include triangle swaps.
          - False: only square swaps.
        - **count** (*bool*) –
          - False: do not count occurrences of subgraphs.
          - True: count individual occurrences of subgraphs via hashes
            :   and store in Python dictionary.

    Warning

    Setting count=True is very slow.

    Returns
    :   **(A, nr\_square\_moves, nr\_triangle\_moves)** – Contains the reshuffled matrix, nr square moves, nr triangle moves.

    Return type
    :   tuple

    Note

    This graph shuffling draws uniformly (unbiased) from all graphs with the given degree distribution.

    Let \(p(k|l)\) be the transition matrix of the Markov chain
    with transitions defined by canonical
    moves 1 on the neuronal graph.
    For simplicity focus first on the case of an excitatory population only.
    On the associated Markov graph states are represented by
    nodes and edges exist between states with strictly positive transition probability.
    Let \(n(k)\) denote the number of neighbors of state \(k\).
    By finiteness of the graph there exists \(\rho > 0\) with \(\max\_k \rho n(k) < 1\).
    The transition matrix can be chosen such that the
    transition from a given state to each neighboring state
    occurs with constant probability \(\rho\) for all neighboring states.
    The probability of staying in a state is
    therefore \(1 - \rho n(k)\) and the transition matrix

    \[\begin{split}p(l|k) =
    \begin{cases}
    \rho & k \sim l \\
    0 & \neg k \sim l \\
    1 - \rho n(k) & l = k
    \end{cases}.\end{split}\]

    The so defined Markov chain is ergodic.
    Aperiodicity holds as long as the Markov graph has
    at least one self-loop which is given in all practical cases.
    Irreducibility holds by connectedness of the Markov graph.
    The Markov graph is connected since the canonical moves
    allow to transition from any graph with a given degree distribution
    to any other graph with the same degree distribution.
    A stationary state exists by symmetry of the transition
    matrix \(p(k|l) = p(l|k)\).
    Thus, the uniform distribution \(\pi\_k = c\) for \(c = \frac{1}{ \sum^{}\_{k} 1}\)
    is a stationary distribution.
    By aperiodicity and irreducibility it is also the unique stationary distribution.

    The same argument holds within the inhibitory
    population only and can be similarly applied to the excitatory
    to inhibitory and the inhibitory to excitatory submatrix.

    1
    :   Roberts, E. S., and A. C. C. Coolen.
        “Unbiased Degree-Preserving Randomization of Directed Binary Networks.”
        Physical Review E 85, no. 4 (April 5, 2012): 46103. doi:10.1103/PhysRevE.85.046103.

# discriminatEM

### Navigation

- Installation
- Model selection from the command line with discriminatEM
- Quickstart
- The connectome package
- License

- Connectome models
- Connectome analysis
- Connectome noise
- Network shuffling
- Path enumeration sampling
- Connectome builder
- Connectome function
- Connectome ABC Tasks
- ABC-SMC
- Parallel job execution
- RNN

### Related Topics

- Documentation overview
  - Previous: Connectome noise
  - Next: Path enumeration sampling

### Quick search

©2017, Emmanuel Klinger, Carsten Marr, Fabian J. Theis, Moritz Helmstaedter.
|
Powered by Sphinx 3.5.4
& Alabaster 0.7.12
