# Supplementary material for: Cellular connectomes as arbiters of local circuit models in the cerebral cortex
Source: Nat Commun. 2021 May 13;12:2785. doi: 10.1038/s41467-021-22856-z (PMC8119988; doi:10.1038/s41467-021-22856-z)
Supplement: Supplementary file 3 — Source Data [file 41467_2021_22856_MOESM3_ESM.zip › doc/abc_api.html]

ABC-SMC — discriminatEM documentation

# ABC-SMC¶

ABC-SMC algorithms for Bayesian model selection.

*class* `abcsmc.``ABCLoader`(*data\_store: abcsmc.loader.SQLDataStore*)¶
:   Bases: `object`

    Load ABC results from database and analyse.

    Parameters
    :   **data\_store** (*DataStore*) – The datastore provides the database’s tables as pandas dataframes.
        Can be a SQLDataStore or pandas.HDFStore.

    `average_mass_at_tround_truth`()¶
    :   Averaged posterior probabilities, grouped by `group_parameters`.

    *property* `confusion_matrices_table`¶
    :   Confusion matrices.

    `confusion_matrix_dict`()¶
    :   Confusion matrices as dict, with keys indicating the sweep parameters.

    *property* `group_parameters`¶
    :   Paramters for grouping ABC sweeps.

    *property* `max_nr_populations`¶
    :   Maximum number of populations.

    `maximum_a_posteriori`()¶
    :   MAP estimates, grouped by `group_parameters`.

    *property* `maxs`¶
    :   Maxima of the results, grouped by the `group_parameters`.

    `means`()¶
    :   Means of the results, grouped by the `group_parameters`.

    *property* `model_names`¶
    :   Unique names of the models found in the database.

    `particles_of_population`(*abc\_smc\_id: int*, *model\_name: str*, *t: int*)¶
    :   Return the particles of a given population.
        Useful if the posterior parameters are of interest.

        Parameters
        :   - **abc\_smc\_id** (*int*) – ID of the ABCSMC run.
            - **model\_name** (*str*) – Name of the model.
            - **t** (*int*) – Population number.

        Returns
        :   **particles** – The particles of the chosen population.

        Return type
        :   DataFrame

    `results`()¶
    :   Final results of the ABC runs.

    `terminated_abc_smc_ids`()¶
    :   IDs of already terminated ABCSMC runs.

*class* `abcsmc.``ABCSMC`(*models: List[Callable[[util.parameters.Parameter], dict]], model\_prior\_distribution: util.random\_variables.RV, model\_perturbation\_kernel: util.random\_variables.ModelPerturbationKernel, parameter\_given\_model\_prior\_distribution: List[util.random\_variables.Distribution], adaptive\_parameter\_perturbation\_kernels: List[Callable[[int, dict], util.random\_variables.Kernel]], distance\_function: abcsmc.distance\_functions.DistanceFunction, eps: abcsmc.epsilon.Epsilon, nr\_particles: int, mapper=<class 'map'>, debug: bool = False, max\_nr\_allowed\_sample\_attempts\_per\_particle: int = 500, min\_nr\_particles\_per\_population: int = 1*)¶
:   Bases: `object`

    Approximate Bayesian Computation - Sequential Monte Carlo (ABCSMC).

    This is an implementation of an ABCSMC algorithm similar to 1

    Parameters
    :   - **models** (*List**[**Callable**[**[**Parameter**]**,* *dict**]**]*) –

          Calling `models[m](par)` returns the calculated summary statistics
          of model `m` with the corresponding parameters `par`.

          Each callable represents thus one single model.
        - **model\_prior\_distribution** (*RV*) – A random variable giving the prior weights of the model classes.
          If the prior is uniform over the model classes
          this is something like `RV("randint", 0, len(models))`.
        - **model\_perturbation\_kernel** (*ModelPerturbationKernel*) – Kernel which governs with which probability to switch the model
          for a given sample.
        - **parameter\_given\_model\_prior\_distribution** (*List**[**Distribution**]*) – A list of prior distributions for the models’ parameters.
          Each list entry is the prior distribution for the corresponding model.
        - **adaptive\_parameter\_perturbation\_kernels** (*List**[**Callable**[**[**int**,* *dict**]**,* *Kernel**]**]*) –

          A list of functions mapping `(t, stat) -> Kernel`, where

          > - `t` is the population nr
          > - `stat` a dictionary of summary statistics.
          >   :   E.g. `stat['std']['parameter_1']` is the standard deviation of `parameter_1`.
          >
          >       > Warning
          >       >
          >       > If a model has only one particle left the standard deviation is zero.

          This callable is called at the beginning of a new population with the statistics dictionary
          from the last population to determine the new parameter perturbation kernel for the next population.
        - **distance\_function** (*DistanceFunction*) – Measures the distance of the tentatively sampled particle to the measured data.
        - **eps** (*Epsilon*) – Returns the current acceptance epsilon.
          This epsilon changes from population to population.
          The eps instance provides the strategy according to which to change it.
        - **mapper** (*map like*) – A callable which behaves like the built-in map function.
          I.e. mapper(f, args) takes a callable `f` and applies it to the arguments in the list `args`.
          This mapper is used for particle sampling.
          It can be a distributed mapper such as the `parallel.sge.SGE` class.
        - **debug** (*bool*) – Whether to output additional debug information.
        - **max\_nr\_allowed\_sample\_attempts\_per\_particle** (*int*) – The maximum number of sample attempts allowed for each particle.
          If this number is reached, the sampling for a particle is stopped.
          Hence, a population may return with less particles than started.
          This is an approximation to the ABCSMC algorithm which ensures, that
          the algorithm terminates.
        - **min\_nr\_particles\_per\_population** (*int*) – Minimum number of samples which have to be accepted for a population.
          If this number is not reached, the algorithm stops.
          This option, together with the `max_nr_allowed_sample_attempts_per_particle`
          ensures that the algorithm terminates.
          This parameter determines to which extent the
          ABCSMC algorithm is approximated.

    1
    :   Toni, Tina, and Michael P. H. Stumpf.
        “Simulation-Based Model Selection for Dynamical
        Systems in Systems and Population Biology.”
        Bioinformatics 26, no. 1 (2010):
        104–10. doi:10.1093/bioinformatics/btp619.

    `do_not_stop_when_only_single_model_alive`()¶
    :   Calling this method causes the ABCSMC to still continue if only
        a single model is still alive. This is useful if the interest lies in
        estimating the model parameter as compared to performing model selection.

        The default behavior is to stop when only a single model is alive.

    `run`(*nr\_samples\_per\_particle: List[int]*, *minimum\_epsilon: float*) → abcsmc.storage.History¶
    :   Run the ABCSMC model selection. This method can be called many times. It makes another
        step continuing where it has stopped before.

        It is stopped when the maximum number of populations is reached
        or the `minimum_epsilon` value is reached.

        Parameters
        :   - **nr\_samples\_per\_particle** (*List**[**int**]*) –

              The length of the list determines the maximal number of populations.

              The entries of the list the number of iterated simulations
              in the notation from 2 these are the \(B\_t\).
              Usually, the entries are all ones:
              `nr_samples_per_particle = [1] * nr_populations`.
            - **minimum\_epsilon** (*float*) – Stop if epsilon is smaller than minimum epsilon specified here.

        2
        :   Toni, Tina, David Welch, Natalja Strelkowa, Andreas Ipsen, and Michael P. H. Stumpf. “Approximate Bayesian Computation Scheme for Parameter Inference and Model Selection in Dynamical Systems.” Journal of The Royal Society Interface 6, no. 31 (2009): 187–202. doi:10.1098/rsif.2008.0172.

    `sample_from_prior`() → List[dict]¶
    :   Only sample from prior and return results without changing
        the history. This can be used to get initial samples
        for the distance function or the epsilon to calibrate them.

        Warning

        The sample is cached.

    `set_data`(*observed\_summary\_statistics: dict*, *ground\_truth\_model\_nr\_or\_name: Union[int, str]*, *ground\_truth\_parameter: dict*, *abc\_options: dict*, *model\_names: Iterable[str]*)¶
    :   Set the data to be fitted.

        Parameters
        :   - **observed\_summary\_statistics** (*dict*) –

              **This is the really important parameter here**. It is of the form
              `{'statistic_1' : val_1, 'statistic_2': val_2, ... }`.

              The dictionary provided here represents the measured data.
              Particles during ABCSMC sampling are compared with the summary statistics
              provided here.
            - **ground\_truth\_model\_nr\_or\_name** (*Union**[**int**,* *str**]*) – This is only meta data stored to the database, but not actually used for the ABCSMC algorithm.
              To evaluate the ABCSMC procedure against synthetic samples, this parameter can be used to indicate the
              ground truth model number or name. This helps with further analysis. If actually measured data is used,
              it is recommended to set this parameter to `-1`.
            - **ground\_truth\_parameter** (*dict*) – Similar to `ground_truth_model_nr_or_name`, this is only for recording purposes, but not used in the
              ABCSMC algorithm. This stores the parameters of the ground truth model if it was synthetically
              obtained.
            - **abc\_options** (*dict*) – Has to contain the key “db\_path” which has to be a valid SQLAlchemy database identifier.
              Can contain an arbitrary number of additional keys, only for recording purposes.
              Store arbitrary meta information in this dictionary.
            - **model\_names** (*List**[**str**]*) – Only for recording purposes. Record names of the models.

*class* `abcsmc.``ConstantEpsilon`(*constant\_epsilon\_value: float*)¶
:   Bases: `abcsmc.epsilon.Epsilon`

    Keep epsilon constant over all populations.

    Parameters
    :   **constant\_epsilon\_value** (*float*) – The epsilon value for all populations.

    `__call__`(*t*, *history*)¶
    :   Parameters
        :   - **t** (*int*) – The population number.
            - **history** (*History*) – ABC history object. Can be used to query summary statistics to set the epsilon.

        Returns
        :   **eps** – The new epsilon for population `t`.

        Return type
        :   float

    `get_config`()¶
    :   Return configuration of the distance function.

        Returns
        :   **config** – Dictionary describing the distance function.

        Return type
        :   dict

*class* `abcsmc.``DistanceFunction`¶
:   Bases: `abc.ABC`

    Abstract case class for distance functions.

    Any other distance function should inherit from this class.

    *abstract* `__call__`(*x: dict*, *x\_0: dict*) → float¶
    :   Abstract method. This method has to be overwritten by all concrete implementations.

        Evaluate the distance of the tentatively sampled particles relative to the measured data.

        Parameters
        :   - **x** (*dict*) – Summary statistics of the tentatively sampled parameter.
            - **x\_0** (*dict*) – Summary statistics of the measured data.

        Returns
        :   **distance** – Distance of the tentatively sampled particles to the measured data.

        Return type
        :   float

    `get_config`() → dict¶
    :   Return configuration of the distance function.

        Returns
        :   **config** – Dictionary describing the distance function.

        Return type
        :   dict

    `initialize`(*sample\_from\_prior: List[dict]*)¶
    :   This method is called by the ABCSMC framework before the first usage of the distance function
        and can be used to calibrate it to the statistics of the samples.

        Per default, no calibration is made.

        Parameters
        :   **sample\_from\_prior** (*List**[**dict**]*) – List of dictionaries containig the summary statistics.

    `to_json`() → str¶
    :   Return JSON encoded configuration of the distance function.

        Returns
        :   **json\_str** – JSON encoded string describing the distance function.
            The default implementation is to try to convert the dictionary
            returned by `get_config`.

        Return type
        :   str

*class* `abcsmc.``DistanceFunctionWithMeasureList`(*measures\_to\_use='all'*)¶
:   Bases: `abcsmc.distance_functions.DistanceFunction`

    Base class for distance functions with measure list.

    Parameters
    :   **measures\_to\_use** (*Union**[**str**,* *List**[**str**]**]*) –

        - If set to “all”, all measures are used. This is the default.
        - If a list is provided, the measures in the list are used.

    `get_config`()¶
    :   Return configuration of the distance function.

        Returns
        :   **config** – Dictionary describing the distance function.

        Return type
        :   dict

    `initialize`(*sample\_from\_prior*)¶
    :   This method is called by the ABCSMC framework before the first usage of the distance function
        and can be used to calibrate it to the statistics of the samples.

        Per default, no calibration is made.

        Parameters
        :   **sample\_from\_prior** (*List**[**dict**]*) – List of dictionaries containig the summary statistics.

    `measures_to_use`¶
    :   The measures (summary statistics) to use for distance calculation.

    `sanitize_sample_from_prior`(*sample*)¶
    :   Remove samples in which any of the measures is NaN.
        Added by Alessandro Motta <alessandro.motta@brain.mpg.de>

*class* `abcsmc.``Distribution`(*\*args*, *\*\*kwargs*)¶
:   Bases: `util.parameters.ParameterStructure`

    Distribution of parameters for a model.

    A distribution is a collection of RVs and/or distributions. It is a dictionary-like object
    of random variables or distributions.

    This should be used as prior and also as Kernel density.

    `copy`() → util.random\_variables.Distribution¶
    :   Copy the distribution.

        Returns
        :   **copied\_distribution** – A copy of the distribution.

        Return type
        :   Distribution

    *static* `from_dictionary_of_dictionaries`(*dict\_of\_dicts: dict*) → util.random\_variables.Distribution¶
    :   Create distribution from dictionary of dictionaries.

        Parameters
        :   **dict\_of\_dicts** (*dict*) – The keys of the dict indicate the parameters’ names.
            The values are itself dictionaries representing scipy.stats
            distributions. I.e. they have the key “name” and at least one of the keys
            “args” or “kwargs”.

        Returns
        :   **distribution** – Created distribution.

        Return type
        :   Distribution

    `get_parameter_names`() → list¶
    :   Sorted list of parameter names.

        Returns
        :   **sorted\_names** – Sorted list of parameter names.

        Return type
        :   list

    `pdf`(*x: Union[util.parameters.Parameter, dict]*)¶
    :   Get combination of probability density function (for continuous variables) and
        probability mass function (for discrete variables) at point x.

        Parameters
        :   **x** (*Union**[**Parameter**,* *dict**]*) – Evaluate at the given Parameter `x`.

    `rvs`() → util.parameters.Parameter¶
    :   Sample from joint distribution.

        Returns
        :   **parameter** – A parameter which was sampled.

        Return type
        :   Parameter

    `update_random_variables`(*\*\*random\_variables*)¶
    :   Update random variables within the distribution.

        Parameters
        :   **\*\*random\_variables** – keywords are the parameters’ names, the values are random variables.

*class* `abcsmc.``EmptyMultivariateMultiTypeNormalDistribution`¶
:   Bases: `object`

    Empty multivariate distribution.

    Returns always empty parameters upon sampling.

    `pdf`(*x*)¶
    :   Return always 1.

    `rvs`()¶
    :   Return empty Parameter.

*class* `abcsmc.``Epsilon`¶
:   Bases: `abc.ABC`

    Abstract epsilon base class.

    This class encapsulates a strategy for setting a new epsilon for each new population.

    *abstract* `__call__`(*t: int*, *history: abcsmc.storage.History*)¶
    :   Parameters
        :   - **t** (*int*) – The population number.
            - **history** (*History*) – ABC history object. Can be used to query summary statistics to set the epsilon.

        Returns
        :   **eps** – The new epsilon for population `t`.

        Return type
        :   float

    `get_config`()¶
    :   Return configuration of the distance function.

        Returns
        :   **config** – Dictionary describing the distance function.

        Return type
        :   dict

    `initialize`(*sample\_from\_prior: List[dict]*, *distance\_to\_ground\_truth\_function: Callable[[dict], float]*)¶
    :   This method is called by the ABCSMC framework before the first usage of the epsilon
        and can be used to calibrate it to the statistics of the samples.

        Per default, no calibration is made.

        Parameters
        :   - **sample\_from\_prior** (*List**[**dict**]*) – List of dictionaries containing the summary statistics.
            - **distance\_to\_ground\_truth\_function** (*Callable**[**[**dict**]**,* *float**]*) – One of the distance functions pre-evaluated at its second argument
              (the one representing the measured data).
              E.g. similar to `lambda x: distance_function(x, x_measured)`.

    `to_json`()¶
    :   Return JSON encoded configuration of the distance function.

        Returns
        :   **json\_str** – JSON encoded string describing the distance function.
            The default implementation is to try to convert the dictionary
            returned by `get_config`.

        Return type
        :   str

*class* `abcsmc.``History`(*db\_path: str*, *nr\_models: int*, *model\_names: List[str]*, *min\_nr\_particles\_per\_population: int*, *debug=False*)¶
:   Bases: `object`

    History for ABCSMC.

    This class records the evolution of the populations and stores the ABCSMC results.

    Parameters
    :   - **db\_path** (*str*) – SQLAlchemy database identifier.
        - **nr\_models** (*int*) – Number of models.
        - **model\_names** (*List**[**str**]*) – List of model names.
        - **min\_nr\_particles\_per\_population** (*int*) – Minimum number of particles per population.
        - **debug** (*bool*) – Whether to print additional debug output.

    Warning

    Most likely this class is never manually instantiated.
    An instance of this class is returned by the `ABCSMC.run` method.
    It can then be used for querying. However, most likely even that won’t be
    used since querying is usually done on the stored database using the abc\_loader.

    `append_population`(*t: int*, *current\_epsilon: float*, *particle\_population: list*)¶
    :   Append population to database.

        Parameters
        :   - **t** (*int*) – Population number.
            - **current\_epsilon** (*float*) – Current epsilon value.
            - **particle\_population** (*list*) – List of sampled particles.

        Returns
        :   **enough\_particles** – Whether enough particles were found in the population.

        Return type
        :   bool

    `done`()¶
    :   Close database sessions and store end time of population.

    `get_complete_population_median`(*t: int*) → float¶
    :   Median of a population’s distances to the measured sample.

        Parameters
        :   **t** (*int*) – Population number.

        Returns
        :   **median** – The median of the distances.

        Return type
        :   float

    *static* `get_cov`(*particles: list*) → Union[util.random\_variables.NonEmptyMultivariateMultiTypeNormalDistribution, util.random\_variables.EmptyMultivariateMultiTypeNormalDistribution]¶
    :   Covariance from particles.

        Parameters
        :   **particles** (*list*) – List of particles.

        Returns
        :   **cov** – The covariance representing distribution.

        Return type
        :   Union[NonEmptyMultivariateMultiTypeNormalDistribution, EmptyMultivariateMultiTypeNormalDistribution]

    `get_distribution`(*t: int*, *m: int*, *parameter: str*) → Tuple[numpy.ndarray]¶
    :   Returns parameter values and weights.

        Parameters
        :   - **t** (*int*) – Population number.
            - **m** (*int*) – Model number.
            - **parameter** (*str*) –

        Returns
        :   **(points, weights)** – The points and their weights.

        Return type
        :   Tuple[np.ndarray]

    `get_model_probabilities`(*t=- 1*) → numpy.ndarray¶
    :   Model probabilities.

        Parameters
        :   **t** (*int*) – Population. Defaults to -1, i.e. the last population.

        Returns
        :   **probabilities** – Model probabilities.

        Return type
        :   np.ndarray

    `get_parameter_std`(*t: int*, *m: int*) → dict¶
    :   Standard deviation of the parameters in a given population.

        Parameters
        :   - **t** (*int*) – Population number.
            - **m** (*int*) – Model number.

        Returns
        :   **std** – Dictionary with keys the parameter names and values their standard deviations.

        Return type
        :   dict

    `get_results`()¶
    :   G the full last record.

    `get_results_distribution`(*m: int*, *parameter: str*) → Tuple[numpy.ndarray]¶
    :   Returns parameter values and weights of the last population.

        Parameters
        :   - **m** (*int*) – Model number.
            - **parameter** (*str*) – Parameter name.

        Returns
        :   **results** – results = (points, weights) with the points and the weights of the last population.

        Return type
        :   Tuple[np.ndarray]

    `get_statistics`(*t: int*) → dict¶
    :   Statistics from particle populations.

        Parameters
        :   **t** (*int*) – Population number.

        Returns
        :   **stat** – List of population statistics at the time t.
            Each list entry corresponds to a model.
            `[{"std": ..., "nr_particles": ..., "cov": ...}, {"std": ..., "nr_particles": ..., "cov": ...}, ... ]`.

        Return type
        :   list

    `nr_of_models_alive`(*t=- 1*) → int¶
    :   Number of models still alive.

        Parameters
        :   **t** (*int*) – Population number.

        Returns
        :   **nr\_alive** – Number of models still alive.

        Return type
        :   int

    `nr_simulations`¶
    :   Only counts the simulations which appear in particles. If a simulation terminated prematurely it is not counted.

    `sample_from_models`(*t: int*) → int¶
    :   Sample from the distribution over models.

        Parameters
        :   **t** (*int*) – Population number.

        Returns
        :   **model\_choice** – This is m\*in the notation from 3 .

        Return type
        :   int

        3
        :   Toni, Tina, and Michael P. H. Stumpf.
            “Simulation-Based Model Selection for Dynamical
            Systems in Systems and Population Biology.”
            Bioinformatics 26, no. 1 (2010):
            104–10. doi:10.1093/bioinformatics/btp619.

    `sample_from_population`(*t: int*, *m: int*) → Optional[abcsmc.storage.Parameter]¶
    :   Sample from population.

        Parameters
        :   - **t** (*int*) – Population number.
            - **m** (*int*) – Model number.

        Returns
        :   **sample** – Returns None if population t,m is empty, otherwise a sample parameter from it.

        Return type
        :   Union[Parameter, None]

    `store_initial_data`(*ground\_truth\_model\_nr\_or\_name: Union[int, str]*, *options*, *observed\_summary\_statistics: dict*, *ground\_truth\_parameter: dict*, *distance\_function\_json\_str: str*, *eps\_function\_json\_str: str*)¶
    :   Store the initial configuration data.

        Parameters
        :   - **ground\_truth\_model\_nr\_or\_name** (*Union**[**int**,* *str**]*) – number or name of the ground truth model.
            - **observed\_summary\_statistics** (*dict*) – the measured summary statistics.
            - **ground\_truth\_parameter** (*dict*) – the ground truth parameters.
            - **distance\_function\_json\_str** (*str*) – the distance function represented as json string.
            - **eps\_function\_json\_str** (*str*) – the epsilon represented as json string.

    *property* `t`¶
    :   Current population.

    *property* `total_nr_simulations`¶
    :   Total number of simulations/samples.

*class* `abcsmc.``Kernel`(*\*distribution*, *\*\*random\_variables*)¶
:   Bases: `object`

    A Kernel of the form K(x,y) = K(x-y).

    Can be initialized from a distribution or using individual variables.
    E.g. do `Kernel(distribution)` or `Kernel(par_name_1=rv1, par_name2=rv2)`.

    If X is a given RV with pdf f, then K(x,y) = f(x-y).

    `add_random_variables`(*\*\*random\_variables*)¶
    :   Add random variables to kernel.

        Parameters
        :   **random\_variables** (*keyword arguments*) – Keys are the names, values the random variables.

    `pdf`(*x*, *y*)¶
    :   Return density \(K(x,y)\),
        i.e., the probability of transitioning from y to x.

    `rvs`(*theta*)¶
    :   Return sample from \(K( \cdot, theta)\).

*class* `abcsmc.``ListEpsilon`(*values: List[float]*)¶
:   Bases: `abcsmc.epsilon.Epsilon`

    Return epsilon values from a predefined list.

    Parameters
    :   **values** (*List**[**float**]*) – List of epsilon values.
        `values[k]` is the value for population k.

    `__call__`(*t*, *history*)¶
    :   Parameters
        :   - **t** (*int*) – The population number.
            - **history** (*History*) – ABC history object. Can be used to query summary statistics to set the epsilon.

        Returns
        :   **eps** – The new epsilon for population `t`.

        Return type
        :   float

    `get_config`()¶
    :   Return configuration of the distance function.

        Returns
        :   **config** – Dictionary describing the distance function.

        Return type
        :   dict

*class* `abcsmc.``LowerBoundDecorator`(*component: util.random\_variables.RV*, *lower\_bound: float*)¶
:   Bases: `util.random_variables.RVDecorator`

    Impose a strict lower bound on a random variable.
    Condition RV X to “X > lower bound”.
    In particular P(X = lower\_bound) = 0.

    Note

    Sampling is done via rejection. Up to 10000 samples are taken from the decorated RV.
    The first sample within the permitted range is then taken. Otherwise None is returned.

    Parameters
    :   - **component** (*RV*) – The decorated random variable.
        - **lower\_bound** (*float*) – The lower bound.

    `cdf`(*x*)¶
    :   Cumulative distribution function.

        Parameters
        :   **x** (*float*) – Cumulative distribution function at x.

        Returns
        :   **density** – Cumulative distribution function at x.

        Return type
        :   float

    `copy`()¶
    :   Copy the random variable.

        Returns
        :   **copied\_rv** – A copy of the random variable.

        Return type
        :   RVBase

    `decorator_repr`()¶
    :   Represent the decorator itself.

        Template method.

        The `__repr__` method used `decorator_repr` and the `__repr__` of the
        decorated RV to build a combined representation.

        Returns
        :   **decorator\_repr** – A string representing the decorator only.

        Return type
        :   str

    `pdf`(*x*)¶
    :   Probability density function.

        Parameters
        :   **x** (*float*) – Probability density at x.

        Returns
        :   **density** – Probability density at x.

        Return type
        :   float

    `pmf`(*x*)¶
    :   Probability mass function.

        Parameters
        :   **x** (*int*) – Probability mass at `x`.

        Returns
        :   **mass** – The mass at `x`.

        Return type
        :   float

    `rvs`()¶
    :   Sample from the RV.

        Returns
        :   **sample** – A sample from the random variable.

        Return type
        :   float

*class* `abcsmc.``MedianEpsilon`(*initial\_epsilon: Union[str, int] = 'from\_sample'*, *median\_multiplier: float = 1*)¶
:   Bases: `abcsmc.epsilon.Epsilon`

    Calculate epsilon as median from the last population.

    Parameters
    :   - **initial\_epsilon** (*Union**[**str**,* *int**]*) –
          - If ‘from\_sample’, then the initial median is calculated from samples as its median.
          - If a number is given, this number is used.
        - **median\_multiplier** (*float*) – Multiplies the median by that number. Also applies it
          to the initial median if it is calculated from samples.
          However, it does **not** apply to the initial median if
          it is given as a number.

    `__call__`(*t*, *history*)¶
    :   Parameters
        :   - **t** (*int*) – The population number.
            - **history** (*History*) – ABC history object. Can be used to query summary statistics to set the epsilon.

        Returns
        :   **eps** – The new epsilon for population `t`.

        Return type
        :   float

    `get_config`()¶
    :   Return configuration of the distance function.

        Returns
        :   **config** – Dictionary describing the distance function.

        Return type
        :   dict

    `initialize`(*sample\_from\_prior*, *distance\_to\_ground\_truth\_function*)¶
    :   This method is called by the ABCSMC framework before the first usage of the epsilon
        and can be used to calibrate it to the statistics of the samples.

        Per default, no calibration is made.

        Parameters
        :   - **sample\_from\_prior** (*List**[**dict**]*) – List of dictionaries containing the summary statistics.
            - **distance\_to\_ground\_truth\_function** (*Callable**[**[**dict**]**,* *float**]*) – One of the distance functions pre-evaluated at its second argument
              (the one representing the measured data).
              E.g. similar to `lambda x: distance_function(x, x_measured)`.

*class* `abcsmc.``MinMaxDistanceFunction`(*measures\_to\_use='all'*)¶
:   Bases: `abcsmc.distance_functions.RangeEstimatorDistanceFunction`

    Calculate upper and lower margins as max and min of the parameters.

    *static* `lower`(*parameter\_list*)¶
    :   Calculate the lower margin form a list of parameter values.

        Parameters
        :   **parameter\_list** (*List**[**float**]*) – List of values of a parameter.

        Returns
        :   **lower\_margin** – The lower margin of the range calculated from these parameters.

        Return type
        :   float

    *static* `upper`(*parameter\_list*)¶
    :   Calculate the upper margin form a list of parameter values.

        Parameters
        :   **parameter\_list** (*List**[**float**]*) – List of values of a parameter.

        Returns
        :   **upper\_margin** – The upper margin of the range calculated from these parameters.

        Return type
        :   float

*class* `abcsmc.``ModelPerturbationKernel`(*nr\_of\_models: int*, *probability\_to\_stay: Optional[float] = None*)¶
:   Bases: `object`

    Model perturbation kernel.

    Parameters
    :   - **nr\_of\_models** (*int*) – Number of models.
        - **probability\_to\_stay** (*Union**[**float**,* *None**]*) – If `None`, probability to stay is set to 1/nr\_of\_models.
          Otherwise, the supplied value is used.

    `pmf`(*n: int*, *m: int*) → float¶
    :   Parameters
        :   - **n** (*int*) – Model target number.
            - **m** (*int*) – Model source number.

        Returns
        :   **probability** – Probability with which to jump from `m` to `n`.

        Return type
        :   float

    `rvs`(*m: int*) → int¶
    :   Sample a Kernel jump from model `m` to another model.

        Parameters
        :   **m** (*int*) – Model source number.

        Returns
        :   **target** – Target model number.

        Return type
        :   int

`abcsmc.``MultivariateMultiTypeNormalDistribution`(*covariance\_matrix*, *parameter\_names*, *parameter\_types*, *zero\_covariance\_substitutes=0.0001*) → Union[util.random\_variables.NonEmptyMultivariateMultiTypeNormalDistribution, util.random\_variables.EmptyMultivariateMultiTypeNormalDistribution]¶
:   Factory function for multivariate and multitype normal distribution.

    This distribution is essentially a multivariate normal, but takes into account
    if a type is an integer and returns it always as rounded integer.
    This is useful if some of the model parameters are discrete.

    Parameters
    :   - **covariance\_matrix** (*np.ndarray*) – 2D array. The covariance matrix.
        - **parameter\_names** (*List**[**str**]*) – List of parameter names.
        - **parameter\_types** (*list*) – A list containing `int` and/or `float` to indicate whether a parameter
          is of type `float` or `int`.
        - **zero\_covariance\_substitutes** (*float*) – Substitutes zero variance entries on the diagonal of the diagonal representation
          of the covariance matrix.

    Returns
    :   **multivariate\_distribution** – Returns NonEmptyMultivariateMultiTypeNormalDistribution of len(parameter\_names) > 0 otherwise
        a EmptyMultivariateMultiTypeNormalDistribution is returned.

    Return type
    :   Union[NonEmptyMultivariateMultiTypeNormalDistribution, EmptyMultivariateMultiTypeNormalDistribution]

*class* `abcsmc.``NonEmptyMultivariateMultiTypeNormalDistribution`(*covariance\_matrix: numpy.ndarray*, *parameter\_names: List[str]*, *parameter\_types: list*, *zero\_covariance\_substitutes=0.0001*)¶
:   Bases: `object`

    Multivariate and multitype normal distribution.

    This distribution is essentially a multivariate normal, but takes into account
    if a type is an integer and returns it always as rounded integer.
    This is useful if some of the model parameters are discrete.

    Parameters
    :   - **covariance\_matrix** (*np.ndarray*) – 2D array. The covariance matrix.
        - **parameter\_names** (*List**[**str**]*) – List of parameter names.
        - **parameter\_types** (*list*) – A list containing `int` and/or `float` to indicate whether a parameter
          is of type `float` or `int`.
        - **zero\_covariance\_substitutes** (*float*) – Substitutes zero variance entries on the diagonal of the covariance matrix.

    `pdf`(*x: dict*) → float¶
    :   Probability density function at x.

        Parameters
        :   **x** (*dict*) – Where to predict.

        Returns
        :   **density** – The probability density.

        Return type
        :   float

    `rvs`() → util.parameters.Parameter¶
    :   Sample from distribution.

*class* `abcsmc.``PCADistanceFunction`(*measures\_to\_use='all'*)¶
:   Bases: `abcsmc.distance_functions.DistanceFunctionWithMeasureList`

    Calculate distance in whitened coordinates.

    A whitening transformation \(W\) is calculated from an initial sample.
    The distance is measured as Euclidean distance in the transformed space. I.e

    \[d(x,y) = \| Wx - Wy \|.\]

    `__call__`(*x*, *y*)¶
    :   Abstract method. This method has to be overwritten by all concrete implementations.

        Evaluate the distance of the tentatively sampled particles relative to the measured data.

        Parameters
        :   - **x** (*dict*) – Summary statistics of the tentatively sampled parameter.
            - **x\_0** (*dict*) – Summary statistics of the measured data.

        Returns
        :   **distance** – Distance of the tentatively sampled particles to the measured data.

        Return type
        :   float

    `initialize`(*sample\_from\_prior*)¶
    :   This method is called by the ABCSMC framework before the first usage of the distance function
        and can be used to calibrate it to the statistics of the samples.

        Per default, no calibration is made.

        Parameters
        :   **sample\_from\_prior** (*List**[**dict**]*) – List of dictionaries containig the summary statistics.

*class* `abcsmc.``Parameter`(*\*args*, *\*\*kwargs*)¶
:   Bases: `util.parameters.ParameterStructure`

    A single model parameter.

    Parameters are a dictionary with the additional functionality
    to add and subtract parameters.

    I.e. `par_1 + par_2` adds key wise.

    `copy`() → util.parameters.Parameter¶
    :   Copy the parameter.

*class* `abcsmc.``PercentileDistanceFunction`(*measures\_to\_use='all'*)¶
:   Bases: `abcsmc.distance_functions.RangeEstimatorDistanceFunction`

    Calculate normalization 20% and 80% from percentiles as lower and upper margins.

    `PERCENTILE` *= 20*¶
    :   The percentiles

    `get_config`()¶
    :   Return configuration of the distance function.

        Returns
        :   **config** – Dictionary describing the distance function.

        Return type
        :   dict

    *static* `lower`(*measures*)¶
    :   Calculate the lower margin form a list of parameter values.

        Parameters
        :   **parameter\_list** (*List**[**float**]*) – List of values of a parameter.

        Returns
        :   **lower\_margin** – The lower margin of the range calculated from these parameters.

        Return type
        :   float

    *static* `upper`(*measures*)¶
    :   Calculate the upper margin form a list of parameter values.

        Parameters
        :   **parameter\_list** (*List**[**float**]*) – List of values of a parameter.

        Returns
        :   **upper\_margin** – The upper margin of the range calculated from these parameters.

        Return type
        :   float

*class* `abcsmc.``RV`(*name: str*, *\*args*, *\*\*kwargs*)¶
:   Bases: `util.random_variables.RVBase`

    Concrete random variable.

    Parameters
    :   - **name** (*str*) – Name of the distribution as in `scipy.stats`.
        - **args** – Arguments as in `scipy.stats` matching the distribution with name “name”.

    kwargs:
    :   Keyword arguments as in `scipy.stats` matching the distribution with name “name”.

    `cdf`(*x*)¶
    :   Cumulative distribution function.

        Parameters
        :   **x** (*float*) – Cumulative distribution function at x.

        Returns
        :   **density** – Cumulative distribution function at x.

        Return type
        :   float

    `copy`()¶
    :   Copy the random variable.

        Returns
        :   **copied\_rv** – A copy of the random variable.

        Return type
        :   RVBase

    `distribution`¶
    :   the scipy.stats. … distribution object

    *static* `from_dictionary`(*dictionary: dict*) → util.random\_variables.RV¶
    :   Construct random variable from dictionary.

        Parameters
        :   **dictionary** (*dict*) –

            A dictionary with the keys

            > - ”name” (mandatory)
            > - ”args” (optional)
            > - ”kwargs” (optional)

            as in scipy.stats.

        Note

        Either the “args” or the “kwargs” key has to be present.

    `pdf`(*x*)¶
    :   Probability density function.

        Parameters
        :   **x** (*float*) – Probability density at x.

        Returns
        :   **density** – Probability density at x.

        Return type
        :   float

    `pmf`(*x*)¶
    :   Probability mass function.

        Parameters
        :   **x** (*int*) – Probability mass at `x`.

        Returns
        :   **mass** – The mass at `x`.

        Return type
        :   float

    `rvs`()¶
    :   Sample from the RV.

        Returns
        :   **sample** – A sample from the random variable.

        Return type
        :   float

*class* `abcsmc.``RVBase`¶
:   Bases: `abc.ABC`

    Random variable abstract base class.

    Note

    The reason we introduced another random variable class is that `scipy.stats` distributions are not pickleable.
    This class is a thin wrapper around `scipy.stats` distributions to make them pickleable.
    It is important to be able to pickle them to execute the ACBSMC algorithm in a distributed cluster
    environment.

    *abstract* `cdf`(*x: float*) → float¶
    :   Cumulative distribution function.

        Parameters
        :   **x** (*float*) – Cumulative distribution function at x.

        Returns
        :   **density** – Cumulative distribution function at x.

        Return type
        :   float

    *abstract* `copy`() → util.random\_variables.RVBase¶
    :   Copy the random variable.

        Returns
        :   **copied\_rv** – A copy of the random variable.

        Return type
        :   RVBase

    *abstract* `pdf`(*x: float*) → float¶
    :   Probability density function.

        Parameters
        :   **x** (*float*) – Probability density at x.

        Returns
        :   **density** – Probability density at x.

        Return type
        :   float

    *abstract* `pmf`(*x*) → float¶
    :   Probability mass function.

        Parameters
        :   **x** (*int*) – Probability mass at `x`.

        Returns
        :   **mass** – The mass at `x`.

        Return type
        :   float

    *abstract* `rvs`() → float¶
    :   Sample from the RV.

        Returns
        :   **sample** – A sample from the random variable.

        Return type
        :   float

*class* `abcsmc.``RVDecorator`(*component: util.random\_variables.RVBase*)¶
:   Bases: `util.random_variables.RVBase`

    Random variable decorater base class.

    Implement a decorator pattern.

    Further decorators should derive from this class.

    It stores the decorated random variable in `self.component`.

    Overwrite the method `decorator_repr` to represent the decorator type.
    The decorated variable will then be automatically included in the call to `__repr__`.

    Parameters
    :   **component** (*RVBase*) – The random variable to be decorated.

    `cdf`(*x*)¶
    :   Cumulative distribution function.

        Parameters
        :   **x** (*float*) – Cumulative distribution function at x.

        Returns
        :   **density** – Cumulative distribution function at x.

        Return type
        :   float

    `component`¶
    :   The decorated random variable

    `copy`()¶
    :   Copy the random variable.

        Returns
        :   **copied\_rv** – A copy of the random variable.

        Return type
        :   RVBase

    `decorator_repr`() → str¶
    :   Represent the decorator itself.

        Template method.

        The `__repr__` method used `decorator_repr` and the `__repr__` of the
        decorated RV to build a combined representation.

        Returns
        :   **decorator\_repr** – A string representing the decorator only.

        Return type
        :   str

    `pdf`(*x*)¶
    :   Probability density function.

        Parameters
        :   **x** (*float*) – Probability density at x.

        Returns
        :   **density** – Probability density at x.

        Return type
        :   float

    `pmf`(*x*)¶
    :   Probability mass function.

        Parameters
        :   **x** (*int*) – Probability mass at `x`.

        Returns
        :   **mass** – The mass at `x`.

        Return type
        :   float

    `rvs`()¶
    :   Sample from the RV.

        Returns
        :   **sample** – A sample from the random variable.

        Return type
        :   float

*class* `abcsmc.``RangeEstimatorDistanceFunction`(*measures\_to\_use='all'*)¶
:   Bases: `abcsmc.distance_functions.DistanceFunctionWithMeasureList`

    Abstract base class for distance functions whose estimate is based on a range.

    It defines the two template methods `lower` and `upper`.

    Hence

    \[d(x, y) = \sum\_{i \in \text{measures}} \left | \frac{x\_i - y\_i}{u\_i - l\_i} \right |,\]

    where \(l\_i\) and \(u\_i\) are the lower and upper margins for measure \(i\).

    `__call__`(*x*, *y*)¶
    :   Abstract method. This method has to be overwritten by all concrete implementations.

        Evaluate the distance of the tentatively sampled particles relative to the measured data.

        Parameters
        :   - **x** (*dict*) – Summary statistics of the tentatively sampled parameter.
            - **x\_0** (*dict*) – Summary statistics of the measured data.

        Returns
        :   **distance** – Distance of the tentatively sampled particles to the measured data.

        Return type
        :   float

    `get_config`()¶
    :   Return configuration of the distance function.

        Returns
        :   **config** – Dictionary describing the distance function.

        Return type
        :   dict

    `initialize`(*sample\_from\_prior*)¶
    :   This method is called by the ABCSMC framework before the first usage of the distance function
        and can be used to calibrate it to the statistics of the samples.

        Per default, no calibration is made.

        Parameters
        :   **sample\_from\_prior** (*List**[**dict**]*) – List of dictionaries containig the summary statistics.

    *static* `lower`(*parameter\_list: List[float]*)¶
    :   Calculate the lower margin form a list of parameter values.

        Parameters
        :   **parameter\_list** (*List**[**float**]*) – List of values of a parameter.

        Returns
        :   **lower\_margin** – The lower margin of the range calculated from these parameters.

        Return type
        :   float

    *static* `upper`(*parameter\_list: List[float]*)¶
    :   Calculate the upper margin form a list of parameter values.

        Parameters
        :   **parameter\_list** (*List**[**float**]*) – List of values of a parameter.

        Returns
        :   **upper\_margin** – The upper margin of the range calculated from these parameters.

        Return type
        :   float

*class* `abcsmc.``SQLDataStore`(*db: str*)¶
:   Bases: `object`

    SQLData store for the ABCLoader class.

    Parameters
    :   **db** (*str*) – SQLAlchemy connection string.
        E.g.: sqlite:////home/user/my\_database.db.

*class* `abcsmc.``ZScoreDistanceFunction`(*measures\_to\_use='all'*)¶
:   Bases: `abcsmc.distance_functions.DistanceFunctionWithMeasureList`

    Calculate distance as sum of ZScores over the selected measures.
    The measured data is the reference for the ZScore.

    Hence

    \[d(x, y) = \sum\_{i \in \text{measures}} \left| \frac{x\_i-y\_i}{y\_i} \right|.\]

    `__call__`(*x*, *y*)¶
    :   Abstract method. This method has to be overwritten by all concrete implementations.

        Evaluate the distance of the tentatively sampled particles relative to the measured data.

        Parameters
        :   - **x** (*dict*) – Summary statistics of the tentatively sampled parameter.
            - **x\_0** (*dict*) – Summary statistics of the measured data.

        Returns
        :   **distance** – Distance of the tentatively sampled particles to the measured data.

        Return type
        :   float

# discriminatEM

### Navigation

- Installation
- Model selection from the command line with discriminatEM
- Quickstart
- The connectome package
- License

- Connectome models
- Connectome analysis
- Connectome noise
- Network shuffling
- Path enumeration sampling
- Connectome builder
- Connectome function
- Connectome ABC Tasks
- ABC-SMC
- Parallel job execution
- RNN

### Related Topics

- Documentation overview
  - Previous: Connectome ABC Tasks
  - Next: Parallel job execution

### Quick search

©2017, Emmanuel Klinger, Carsten Marr, Fabian J. Theis, Moritz Helmstaedter.
|
Powered by Sphinx 3.5.4
& Alabaster 0.7.12
