# Supplementary material for: Cellular connectomes as arbiters of local circuit models in the cerebral cortex
Source: Nat Commun. 2021 May 13;12:2785. doi: 10.1038/s41467-021-22856-z (PMC8119988; doi:10.1038/s41467-021-22856-z)
Supplement: Supplementary file 3 — Source Data [file 41467_2021_22856_MOESM3_ESM.zip › doc/parallel_api.html]

Parallel job execution — discriminatEM documentation

# Parallel job execution¶

*class* `parallel.``DefaultContext`(*tmp\_path*, *job\_nr*)¶
:   Does nothing special.

*class* `parallel.``ProfilingContext`(*tmp\_path*, *job\_nr*)¶
:   Profiles the running jobs and stores the profiles in the temporary job folder
    in the subdirectory “profiling”.

    Useful for debugging. Do not use in production.

*class* `parallel.``SGE`(*tmp\_directory: Optional[str] = None*, *memory: str = '3G'*, *time\_h: int = 100*, *python\_executable\_path: Optional[str] = None*, *sge\_error\_file: Optional[str] = None*, *sge\_output\_file: Optional[str] = None*, *parallel\_environment=None*, *name='map'*, *ram\_key='h\_vmem'*, *queue=None*, *priority=None*, *num\_threads: int = 1*, *execution\_context=<class 'parallel.execution\_contexts.DefaultContext'>*, *chunk\_size=1*)¶
:   Map a function to be executed on an SGE cluster environment.
    Reads a config file (if it exists) in the home directory which should look as the default
    in parallel.config.

    The mapper reads commonly used parameters from a configuration file stord in `~/.parallel`.
    An example configuration file could look as follows:

    ```
    #~/.parallel
    [DIRECTORIES]
    TMP=/tmp

    [BROKER]
    TYPE=REDIS   # can be SQLITE or REDIS

    [SGE]
    QUEUE=p.openmp
    PARALLEL_ENVIRONMENT=openmp
    PRIORITY=-500

    [REDIS]
    HOST=127.0.0.1
    ```

    Parameters
    :   - **tmp\_directory** (*str* *or* *None*) – Directory where temporary job pickle files are stored.
          If set to None a tmp directory is read from the ‘’~/.parallel’’ configuration file.
          It this file does not exist a tmp directory within the user home directory is created.
        - **memory** (*str*) – RAM requested by each job, e.g. “10G”.
        - **time\_h** (*int*) – Job run time in hours.
        - **python\_executable\_path** (*str* *or* *None*) – The python interpreter which executes the jobs.
          If set to None, the currently executing interpreter is used as returned by `sys.executable`.
        - **sge\_error\_file** (*str* *or* *None*) – File to which stderr messages from workers are stored.
          If set to None, a file within the tmp\_directory is used.
        - **sge\_output\_file** (*str* *or* *None*) – File to which stdout messages from workers are stored.
          If set to None, a file within the tmp\_directory is used.
        - **parallel\_environment** (*str*) – The SGE environment. This is what is passed to the -pe option in the qsub script.
        - **name** (*str*) – A name for the job.
        - **queue** (*str*) – The SGE queue.
        - **priority** (*int*) –

          SGE job priority. A value between -1000 and 0.
          Default: -500

          Note that a priority of 0 automatically enables the reservation flag.
        - **num\_threads** (*int**,* *default = 1*) – Number of threads for each worker.
          This also sets the environment variable MKL\_NUM\_THREADS, OMP\_NUM\_THREADS to the
          sepcified number to handle jobs which use OpenMP etc. correctly.
        - **execution\_context** (`DefaultContext`, `ProfilingContext`, `NamedPrinter`) – Any context manager can be passed here.
          The `__enter__` method is called before evaluating the function on the cluster.
          The `__exit__` method directly after the function run finished.
        - **chunk\_size** (*int**,* *default=1*) –

          Number of tasks executed within one job.

          > Warning
          >
          > If `chunk_size` is larger than 1, this can have bad side effects
          > as all the jobs within one chunk are executed within the python
          > process.

    Returns
    :   **sge** – Configured SGE mapper.

    Return type
    :   SGE

    `map`(*function*, *array*)¶
    :   Does what map(function, array) would do, but does it
        via an array job on the SGE by pickling objects, storing
        them in a temporary folder, submitting them to the SGE and
        then reading and returning the results.

        Parameters
        :   - **function** (*callable*) – The function to be mapped.
            - **array** (*iterable*) – The values to which the function is applied

        Returns
        :   **result\_list** – List of results of function application.
            This list can also contain `Exception` objects.

        Return type
        :   list

*class* `parallel.``Slurm`(*tmp\_directory: Optional[str] = None*, *memory: str = '3G'*, *time\_h: int = 100*, *name='map'*, *python\_executable\_path: Optional[str] = None*, *slurm\_error\_file: Optional[str] = None*, *slurm\_output\_file: Optional[str] = None*, *partition=None*, *niceness=None*, *num\_threads: int = 1*, *execution\_context=<class 'parallel.execution\_contexts.DefaultContext'>*, *chunk\_size=1*)¶
:   Map a function to be executed with Slurm workload manager.
    Reads a config file (if it exists) in the home directory which should look as the default
    in parallel.config.

    The mapper reads commonly used parameters from a configuration file stord in `~/.parallel`.
    An example configuration file could look as follows:

    ```
    #~/.parallel
    [DIRECTORIES]
    TMP=/tmp

    [BROKER]
    TYPE=REDIS   # can be SQLITE or REDIS

    [SLURM]
    PARTITION=p.gaba
    NICENESS=0

    [REDIS]
    HOST=127.0.0.1
    ```

    Parameters
    :   - **tmp\_directory** (*str* *or* *None*) – Directory where temporary job pickle files are stored.
          If set to None a tmp directory is read from the ‘’~/.parallel’’ configuration file.
          It this file does not exist a tmp directory within the user home directory is created.
        - **memory** (*str*) – RAM requested by each job, e.g. “10G”.
        - **time\_h** (*int*) – Job run time in hours.
        - **python\_executable\_path** (*str* *or* *None*) – The python interpreter which executes the jobs.
          If set to None, the currently executing interpreter is used as returned by `sys.executable`.
        - **slurm\_error\_file** (*str* *or* *None*) – File to which stderr messages from workers are stored.
          If set to None, a file within the tmp\_directory is used.
        - **slurm\_output\_file** (*str* *or* *None*) – File to which stdout messages from workers are stored.
          If set to None, a file within the tmp\_directory is used.
        - **name** (*str*) – A name for the job.
        - **partition** (*str*) – The Slurm partition.
        - **niceness** (*int*) – Slurm job niceness.
          Default: 0
        - **num\_threads** (*int**,* *default = 1*) – Number of threads for each worker.
          This also sets the environment variable MKL\_NUM\_THREADS, OMP\_NUM\_THREADS to the
          sepcified number to handle jobs which use OpenMP etc. correctly.
        - **execution\_context** (`DefaultContext`, `ProfilingContext`, `NamedPrinter`) – Any context manager can be passed here.
          The `__enter__` method is called before evaluating the function on the cluster.
          The `__exit__` method directly after the function run finished.
        - **chunk\_size** (*int**,* *default=1*) –

          Number of tasks executed within one job.

          > Warning
          >
          > If `chunk_size` is larger than 1, this can have bad side effects
          > as all the jobs within one chunk are executed within the python
          > process.

    Returns
    :   **slurm** – Configured Slurm mapper.

    Return type
    :   Slurm

    `map`(*function*, *array*)¶
    :   Does what map(function, array) would do, but does it
        via an array job on Slurm by pickling objects, storing
        them in a temporary folder, submitting them to the Slurm and
        then reading and returning the results.

        Parameters
        :   - **function** (*callable*) – The function to be mapped.
            - **array** (*iterable*) – The values to which the function is applied

        Returns
        :   **result\_list** – List of results of function application.
            This list can also contain `Exception` objects.

        Return type
        :   list

`parallel.``sge_available`()¶
:   Makes a simple heuristic test to check if the SGE is available on the machine.
    It tries to execute the `qstat` command. In case it is found, it is assumed
    that the SGE is available.

    Returns
    :   **available** – Whether SGE is available or not.

    Return type
    :   bool

`parallel.``slurm_available`()¶
:   Checks if the Slurm workload manager is available.

# discriminatEM

### Navigation

- Installation
- Model selection from the command line with discriminatEM
- Quickstart
- The connectome package
- License

- Connectome models
- Connectome analysis
- Connectome noise
- Network shuffling
- Path enumeration sampling
- Connectome builder
- Connectome function
- Connectome ABC Tasks
- ABC-SMC
- Parallel job execution
- RNN

### Related Topics

- Documentation overview
  - Previous: ABC-SMC
  - Next: RNN

### Quick search

©2017, Emmanuel Klinger, Carsten Marr, Fabian J. Theis, Moritz Helmstaedter.
|
Powered by Sphinx 3.5.4
& Alabaster 0.7.12
