# Supplementary material for: Cellular connectomes as arbiters of local circuit models in the cerebral cortex
Source: Nat Commun. 2021 May 13;12:2785. doi: 10.1038/s41467-021-22856-z (PMC8119988; doi:10.1038/s41467-021-22856-z)
Supplement: Supplementary file 3 — Source Data [file 41467_2021_22856_MOESM3_ESM.zip › doc/index.html]

discriminatEM — discriminatEM documentation

# discriminatEM¶

## User’s Guide¶

- Installation
  - Preparation
  - PIP
  - Running the unit- and integration tests
- Model selection from the command line with discriminatEM
  - Installation
  - Optional: configuration of the parallel environment
  - Running model selection
  - Examination of the results
  - Reproduction of Figures 4a, 4b and 4c of the manuscript
- Quickstart
  - Network generation
  - Network analysis
  - Noise
- The connectome package
  - Creating networks - Sampling from stochastic network models
  - Networks
  - Network analysis
  - Noise in networks
  - Monte Carlo sampling based analysis
  - Building composite models
  - Functional model testing
  - ABC with network models
- License
  - Author
  - License text

## API reference¶

- Connectome models
- Connectome analysis
- Connectome noise
- Network shuffling
- Path enumeration sampling
- Connectome builder
- Connectome function
  - Tasks
  - Criteria
  - Test runners
  - Test results
- Connectome ABC Tasks
- ABC-SMC
- Parallel job execution
- RNN

## Indices and tables¶

- Index
- Module Index
- Search Page

# discriminatEM

### Navigation

- Installation
- Model selection from the command line with discriminatEM
- Quickstart
- The connectome package
- License

- Connectome models
- Connectome analysis
- Connectome noise
- Network shuffling
- Path enumeration sampling
- Connectome builder
- Connectome function
- Connectome ABC Tasks
- ABC-SMC
- Parallel job execution
- RNN

### Related Topics

- Documentation overview
  - Next: Installation

### Quick search

©2017, Emmanuel Klinger, Carsten Marr, Fabian J. Theis, Moritz Helmstaedter.
|
Powered by Sphinx 3.5.4
& Alabaster 0.7.12
