# Supplementary material for: Cellular connectomes as arbiters of local circuit models in the cerebral cortex
Source: Nat Commun. 2021 May 13;12:2785. doi: 10.1038/s41467-021-22856-z (PMC8119988; doi:10.1038/s41467-021-22856-z)
Supplement: Supplementary file 3 — Source Data [file 41467_2021_22856_MOESM3_ESM.zip › doc/installation.html]

Installation — discriminatEM documentation

# Installation¶

## Preparation¶

This package requires Python 3.6 or later.
Cython and Numpy should be installed before installation of this package,
for example via pip:

```
pip install numpy
pip install cython
```

These packages are required for a successful installation.
If the Anaconda Python distribution is used,
Numpy and Cython should be already included.

## PIP¶

The package is installed from the provided archive via pip:

```
pip install discriminatEM-0.1.3.tar.gz
```

Installation via git will be made available upon publication.

### Windows¶

Visual Studio 2015 is required in order to build this package from source

Warning

It is crucial to mark the ticks for the C++ language and the Visual Studio Common tools
in the Visual Studio 2015 installer. Otherwise an error message
“vcvarsall.bat not found” will be seen.

## Running the unit- and integration tests¶

To run the unit- and integration tests, change into the source code root directory.
This is the directory containing the setup.py file.
Then install the package requirements:

```
pip install -r requirements.txt
```

Compile the binary extensions in-place:

```
python setup.py build_ext --inplace
```

and execute the test suits:

```
python -m unittest
```

This discovers the available tests and runs them.

Some of the tests involve stochastic model sampling and computationally expensive procedures.
These are disabled by default.
To also enable these long running tests, set the environment variable `RUN_LONG_TESTS=true`.

Note

Tests involving stochastic network generation
may fail from time to time although there is no problem.
If an error states that a quantity should be less than some value, but the
actually obtained number is slightly above, this can be safely ignored.

# discriminatEM

### Navigation

- Installation
  - Preparation
  - PIP
  - Running the unit- and integration tests
- Model selection from the command line with discriminatEM
- Quickstart
- The connectome package
- License

- Connectome models
- Connectome analysis
- Connectome noise
- Network shuffling
- Path enumeration sampling
- Connectome builder
- Connectome function
- Connectome ABC Tasks
- ABC-SMC
- Parallel job execution
- RNN

### Related Topics

- Documentation overview
  - Previous: discriminatEM
  - Next: Model selection from the command line with discriminatEM

### Quick search

©2017, Emmanuel Klinger, Carsten Marr, Fabian J. Theis, Moritz Helmstaedter.
|
Powered by Sphinx 3.5.4
& Alabaster 0.7.12
