# Supplementary material for: Cellular connectomes as arbiters of local circuit models in the cerebral cortex
Source: Nat Commun. 2021 May 13;12:2785. doi: 10.1038/s41467-021-22856-z (PMC8119988; doi:10.1038/s41467-021-22856-z)
Supplement: Supplementary file 3 — Source Data [file 41467_2021_22856_MOESM3_ESM.zip › doc/quickstart.html]

Quickstart — discriminatEM documentation

# Quickstart¶

This tutorial provides a brief overview over the basic functionality of this
package.

Matplotlib is imported for visualization

```
import matplotlib.pyplot as plt
```

## Network generation¶

The layered model can be imported form the `connectome.model` package

```
from connectome.model import LL
```

The parameters of a model can be pre-defined

```
ll = LL(nr_neurons=2000, inh_ratio=.1,
        p_exc=.2, p_inh=.5, reciprocity_exc=.2)
ll
```

```
<LL nr_neurons=2000, p_exc=0.2, reciprocity_exc=0.2, inh_ratio=0.1, p_inh=0.5, nr_exc_subpopulations=∅>
```

A network with three layers can now be generated

```
network = ll(nr_exc_subpopulations=3)
network
```

```
<Network nr_exc=1800, nr_inh=200>
```

and visualized

```
plt.matshow(network.adjacency_matrix, cmap="RdBu");
```

Similarly, a network with five layers is generated and visualized, executing

```
network_5 = ll(nr_exc_subpopulations=5)
plt.matshow(network_5.adjacency_matrix, cmap="RdBu");
```

Please note that some parameters in the second call were still specified from
the first call and therefore not passed again.

## Network analysis¶

Summary statistics of a network are calculated with the help of the `connectome.analysis` package.

```
from connectome.analysis import RelativeCycleAnalysis
```

The number of cycles, relative to chance is computed with

```
cycle_analysis = RelativeCycleAnalysis(length=5)
cycle_analysis
```

```
<RelativeCycleAnalysis length=5, network=∅>
```

```
cycle_analysis(network=network)
```

```
{'relative_cycles_5': 0.19235267510727008}
```

The network contains less cycles of length 5 than expected by chance.
A value above 1 indicates more cycles than expected by chance,
a value below 1 indicates less cycles than expected by chance.
The network’s reciprocity is calculated executing

```
from connectome.analysis import RelativeReciprocityEstimator
RelativeReciprocityEstimator(network=network)()
```

```
{'relative_reciprocity_ee': 1.0030832455128713,
 'relative_reciprocity_ei': 0.9991984266639169,
 'relative_reciprocity_ie': 0.999198426663917,
 'relative_reciprocity_ii': 0.9993940697569447}
```

The reciprocity obtained here, is close to the one expected from a pairwise
random network.

## Noise¶

Noise models are implemented in the `connectome.noise` package

```
from connectome.noise import RemoveAddNoiseAndSubsample
```

Random removal of half of the network’s connections with simultaneous
reshuffling of 20% of the connections is simulated via

```
noise = RemoveAddNoiseAndSubsample(subsampling_fraction=.5, fraction_remove_and_add=.2)
noise
```

```
<RemoveAddNoiseAndSubsample subsampling_fraction=<InputChannel: subsampling_fraction=0.5>, network=<InputChannel: network=∅>, fraction_remove_and_add=<InputChannel: fraction_remove_and_add=0.2>>
```

```
noisy_network = noise(network=network)
noisy_network
```

```
<Network nr_exc=900, nr_inh=100>
```

This network has after perturbation only 1000 instead of 2000 neurons before.

```
plt.matshow(noisy_network.adjacency_matrix, cmap="RdBu");
```

Additionally, connections on off-diagonal blocks appear;
these were generated by the shuffling procedure.
The number of cycles is also altered:

```
cycle_analysis(network=noisy_network)
```

```
{'relative_cycles_5': 0.68771057755341169}
```

The perturbed network has more cycles than the noise free version (0.192, see above).

# discriminatEM

### Navigation

- Installation
- Model selection from the command line with discriminatEM
- Quickstart
  - Network generation
  - Network analysis
  - Noise
- The connectome package
- License

- Connectome models
- Connectome analysis
- Connectome noise
- Network shuffling
- Path enumeration sampling
- Connectome builder
- Connectome function
- Connectome ABC Tasks
- ABC-SMC
- Parallel job execution
- RNN

### Related Topics

- Documentation overview
  - Previous: Model selection from the command line with discriminatEM
  - Next: The connectome package

### Quick search

©2017, Emmanuel Klinger, Carsten Marr, Fabian J. Theis, Moritz Helmstaedter.
|
Powered by Sphinx 3.5.4
& Alabaster 0.7.12
