# Supplementary material for: Cellular connectomes as arbiters of local circuit models in the cerebral cortex
Source: Nat Commun. 2021 May 13;12:2785. doi: 10.1038/s41467-021-22856-z (PMC8119988; doi:10.1038/s41467-021-22856-z)
Supplement: Supplementary file 3 — Source Data [file 41467_2021_22856_MOESM3_ESM.zip › readme.pdf]

Model selection from the command line with discriminatEM

Installation

The code is assumed to be run on a Linux environment with Python 3.6 or later installed. For example, the Anaconda Python 3.6 distribution can be used. It is installed via:

```
wget https://repo.continuum.io/archive/Anaconda3-4.4.0-Linux-x86_64.sh
bash Anaconda3-4.4.0-Linux-x86_64.sh
```

following the guided installation process.

> ***Note***
>
> The Anaconda installer asks at the end of the installation whether to use Anaconda Python as the default Python:
>
> ```
> Do you wish the installer to prepend the Anaconda3 install location
> to PATH in your /home/username/.bashrc ? [yes|no]
> [no] >>>
> ```
>
> If this is positively answered, the path to the Anaconda installation is prepended to the `PATH` environment variable and subsequent calls to `pip` (see below) use the Anaconda Python pip (check with the command `which pip`). If the answer is no, it has to be manually ensured that the correct Python installation is used.

Then, *discriminatEM* can be installed from the provided `.tar.gz` file:

```
pip install discriminatEM-0.1.3.tar.gz
```

Optional: configuration of the parallel environment

SGE (UGE) like environments can be used by *discriminatEM*. However, they need to be configured properly. The following information is required

- the path to an existing directory for temporary files
- SGE/UGE queue name
- SGE/UGE parallel environment name
- IP address of a redis-server (installation instructions are provided below)

This information is then assembled in a configuration file `~/.parallel` residing within the home directory. The content should be similar to the following:

```
[DIRECTORIES]
TMP=/path/to/my/tmp

[SGE]
```

```
QUEUE=p.openmp
PARALLEL_ENVIRONMENT=openmp
PRIORITY=-500

[BROKER]
TYPE=REDIS

[REDIS]
HOST=WWW.XXX.YYY.ZZZ
```

The `TMP` directory has to be replaced with an appropriate one and is used to store temporary job files. Also, the values for the SGE `QUEUE` and `PARALLEL_ENVIRONMENT` have to be replaced. Running:

```
qconf -sql
```

yields a list of all defined queues, from which one can be chosen for the `QUEUE`. Running:

```
qconf -spl
```

yields a list of all defined parallel environments, from which one can be chosen for the `PARALLEL_ENVIRONMENT`.

Redis can be installed via:

```
conda install redis
```

and started with:

```
redis-server --protected-mode no
```

The IP address of the host on which redis is running is to be entered for the `HOST` value: `WWW.XXX.YYY.ZZZ` is to be replaced by the IP of the host on which the redis server is running. The IP address can be retrieved with the `ifconfig` command.

Important

The redis-server has to be running throughout the complete ABC run. It manages the communication between the *discriminatEM* main process and the jobs started on the SGE/UGE cluster.

Running model selection

For example:

```
discriminatEM --noise-prior="beta(2, 10)" --noise="[0.2]"\
              --subsampling="[0.9]" abcsmc.db
```

executes an ABCSMC model selection run with a Beta(2, 10) prior on connectome samples perturbed with `noise=0.2` and subsampling (fractional measurement) 0.9. The results are stored in `abcsmc.db`. Additionally, a folder `abcsmc.db.results` is created with confusion matrix plots. The syntax for the noise prior follows the scipy.stats distributions. However, a delta point prior can also be used:

```
discriminatEM --noise-prior=0 --noise="[0.2]"\
              --subsampling="[0.9]" abcsmc.db
```

starts a run with no noise in the prior, but still applied to the connectome sample.

> ***Note***
>
> The arguments `--noise` and `--subsampling` are lists. Several values can be provided here. The full cross product of provided noise and subsampling values is executed.

Examination of the results

Plots and text files are generated in the directory `abcsmc.db.result` (assuming that the chosen database name was `abcsmc.db`, in general, the path is `<database>.results`).

Reproduction of Figures 4a, 4b and 4c of the manuscript

Figure 4a: noise-free:

```
discriminatEM --noise-prior=0 --noise="[0]" fig_4a.db
```

Figure 4b: noise of intensity 0.15 on the samples, but not in the prior:

```
discriminatEM --noise-prior=0 --noise="[0.15]" fig_4b.db
```

Figure 4c: Beta(2,10) prior and noise of intensity 0.15 on the samples:

```
discriminatEM --noise-prior="beta(2,10)" --noise="[0.15]" fig_4c.db
```

(The `--subsampling` argument can be omitted since `--subsampling=1` is the default value and this is the value used for Figure 4)

> ***Note***
>
> The model selection runs are stochastic, therefore the obtained results may vary from the ones in Fig. 4a-c. This is expected especially for the case of a noisy connectome under a noise-free prior (Fig. 4b).
