# Supplementary figures and images for: Cellular connectomes as arbiters of local circuit models in the cerebral cortex
Source: Nat Commun. 2021 May 13;12:2785. doi: 10.1038/s41467-021-22856-z (PMC8119988; doi:10.1038/s41467-021-22856-z)

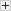

Supplement: Supplementary file 3 — Source Data [file 41467_2021_22856_MOESM3_ESM.zip › doc/_static/plus.png]

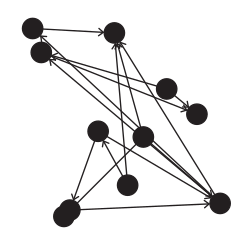

Supplement: Supplementary file 3 — Source Data [file 41467_2021_22856_MOESM3_ESM.zip › doc/_static/logo.png]

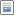

Supplement: Supplementary file 3 — Source Data [file 41467_2021_22856_MOESM3_ESM.zip › doc/_static/file.png]

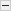

Supplement: Supplementary file 3 — Source Data [file 41467_2021_22856_MOESM3_ESM.zip › doc/_static/minus.png]

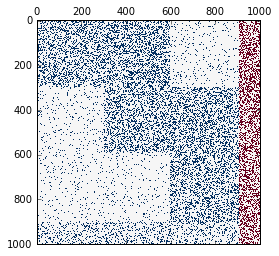

Supplement: Supplementary file 3 — Source Data [file 41467_2021_22856_MOESM3_ESM.zip › doc/_images/quickstart_30_0.png]

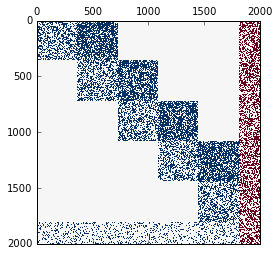

Supplement: Supplementary file 3 — Source Data [file 41467_2021_22856_MOESM3_ESM.zip › doc/_images/quickstart_12_0.png]

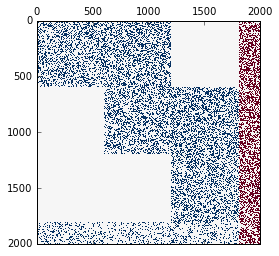

Supplement: Supplementary file 3 — Source Data [file 41467_2021_22856_MOESM3_ESM.zip › doc/_images/quickstart_10_0.png]

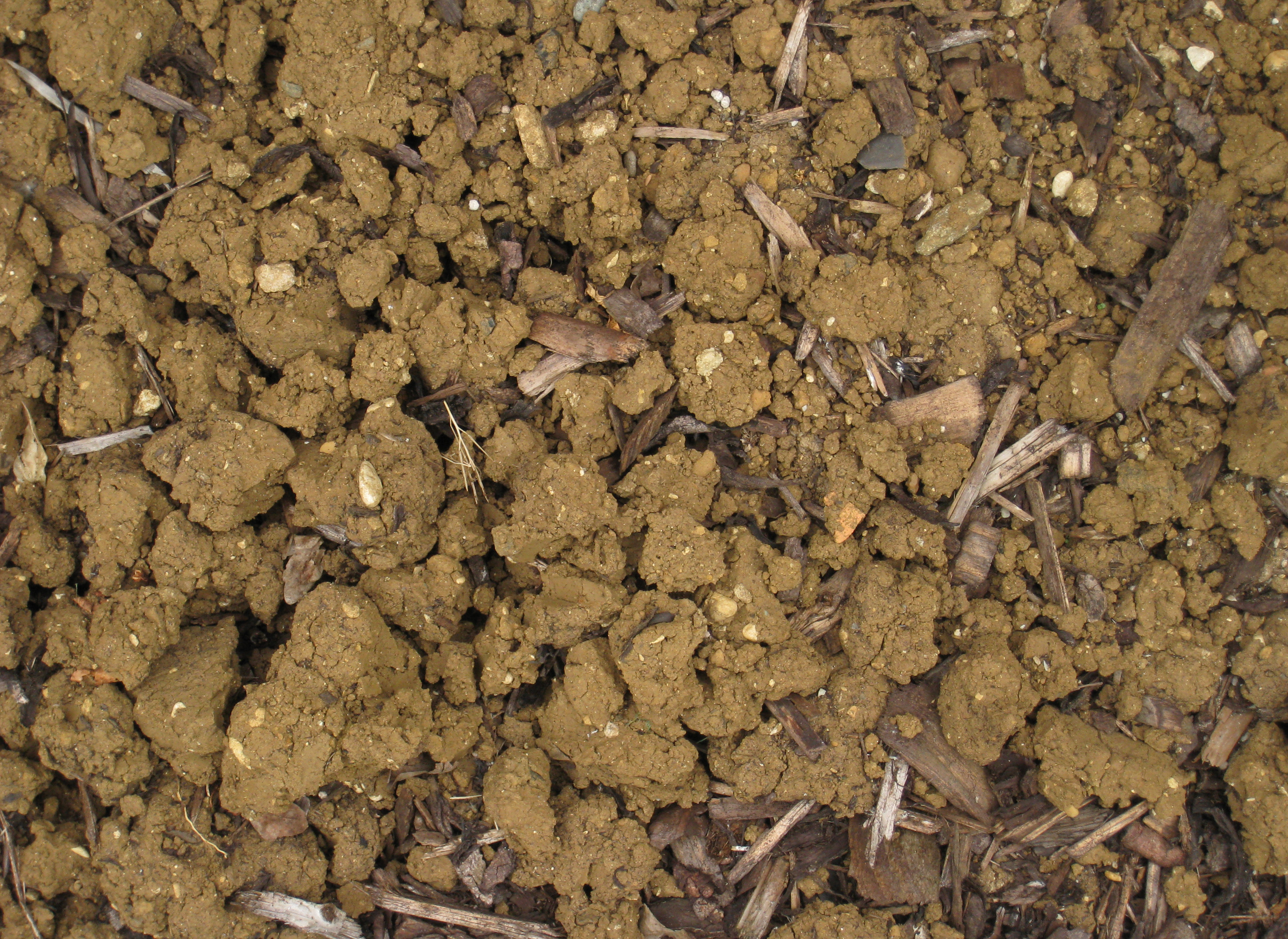

Supplement: Supplementary file 3 — Source Data [file 41467_2021_22856_MOESM3_ESM.zip › figure-7/python/texture/data/01_cracked_soil.JPG]

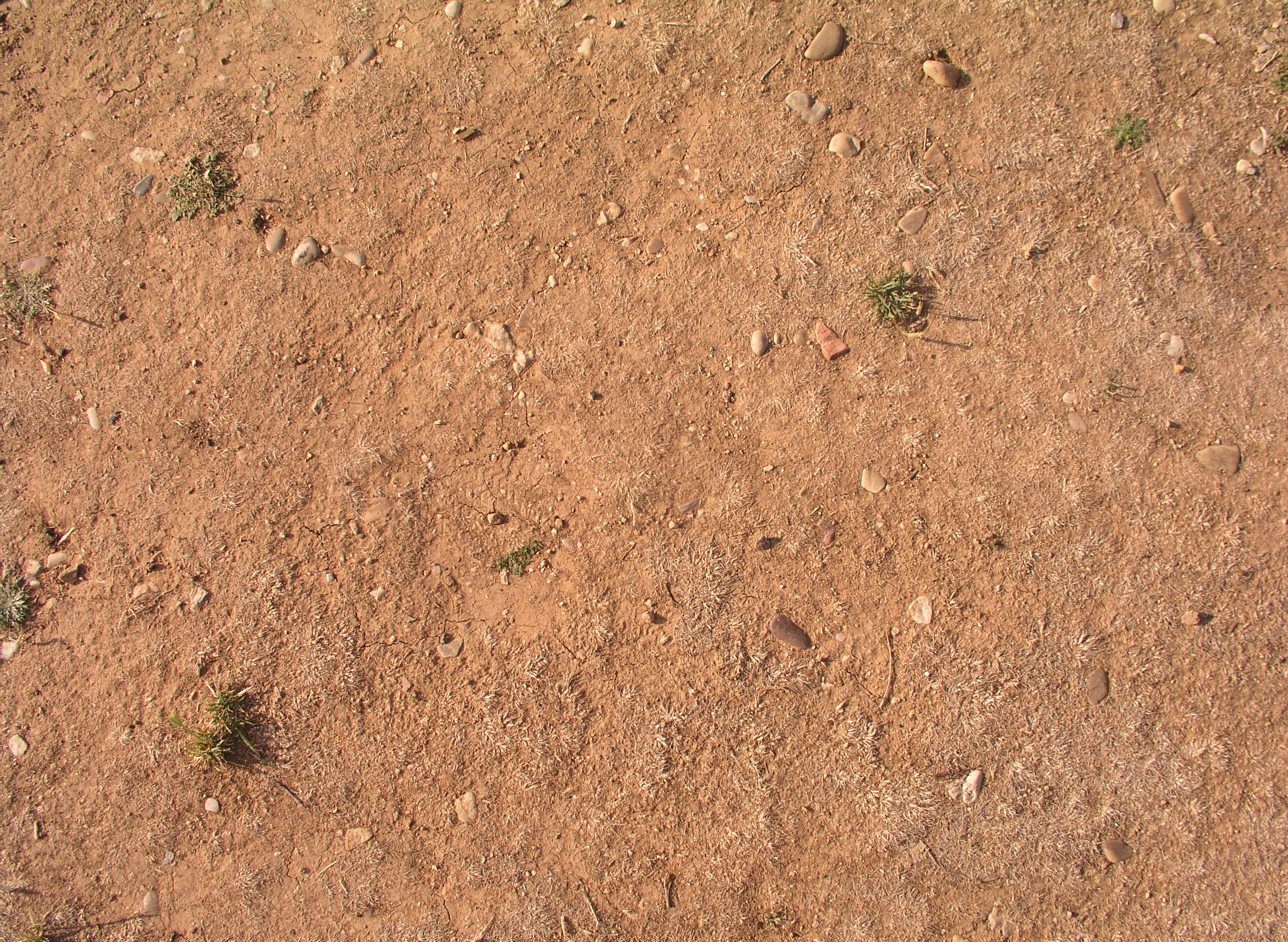

Supplement: Supplementary file 3 — Source Data [file 41467_2021_22856_MOESM3_ESM.zip › figure-7/python/texture/data/00_bare_earth.JPG]

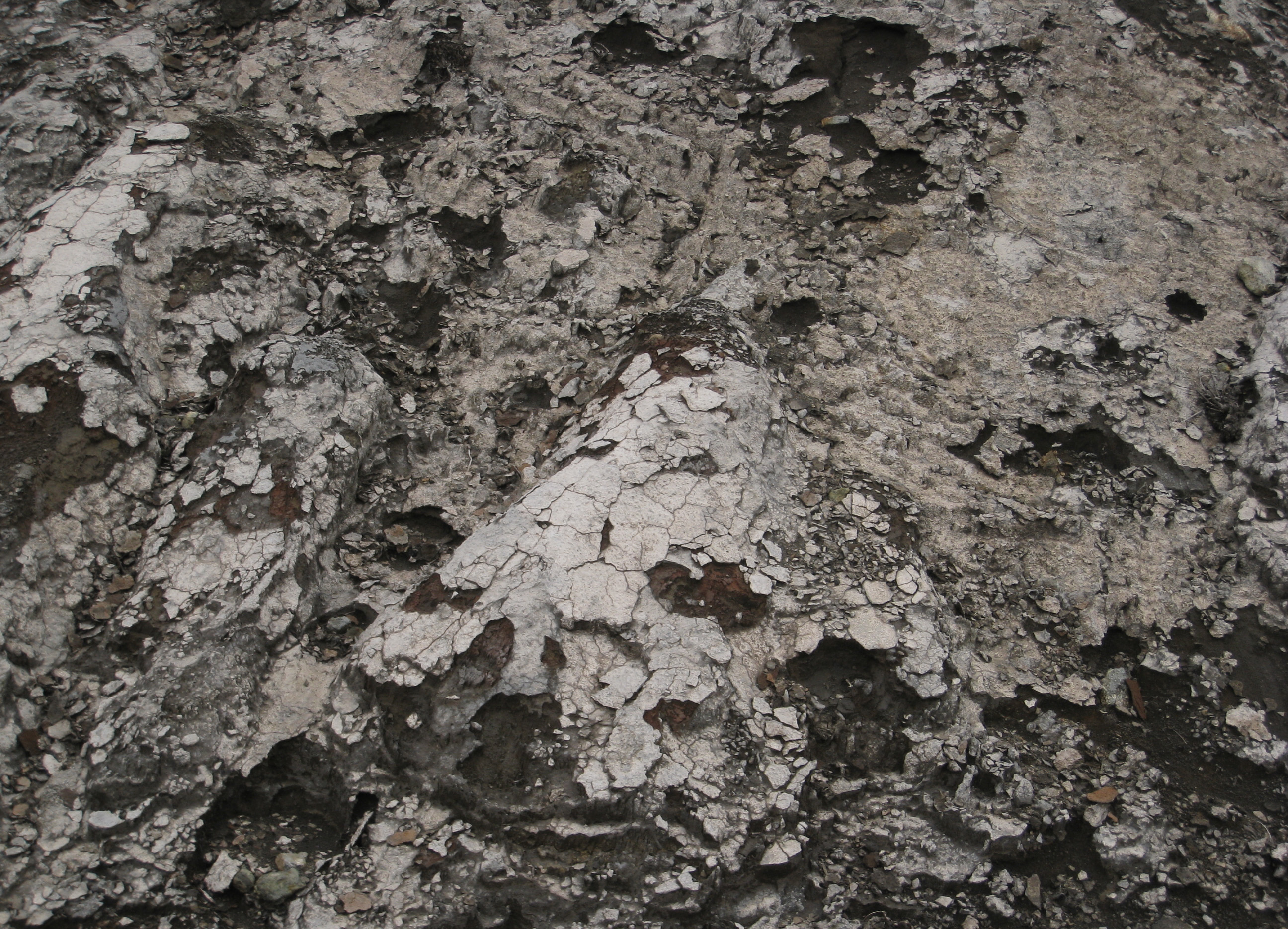

Supplement: Supplementary file 3 — Source Data [file 41467_2021_22856_MOESM3_ESM.zip › figure-7/python/texture/data/04_splintered_muddy.JPG]

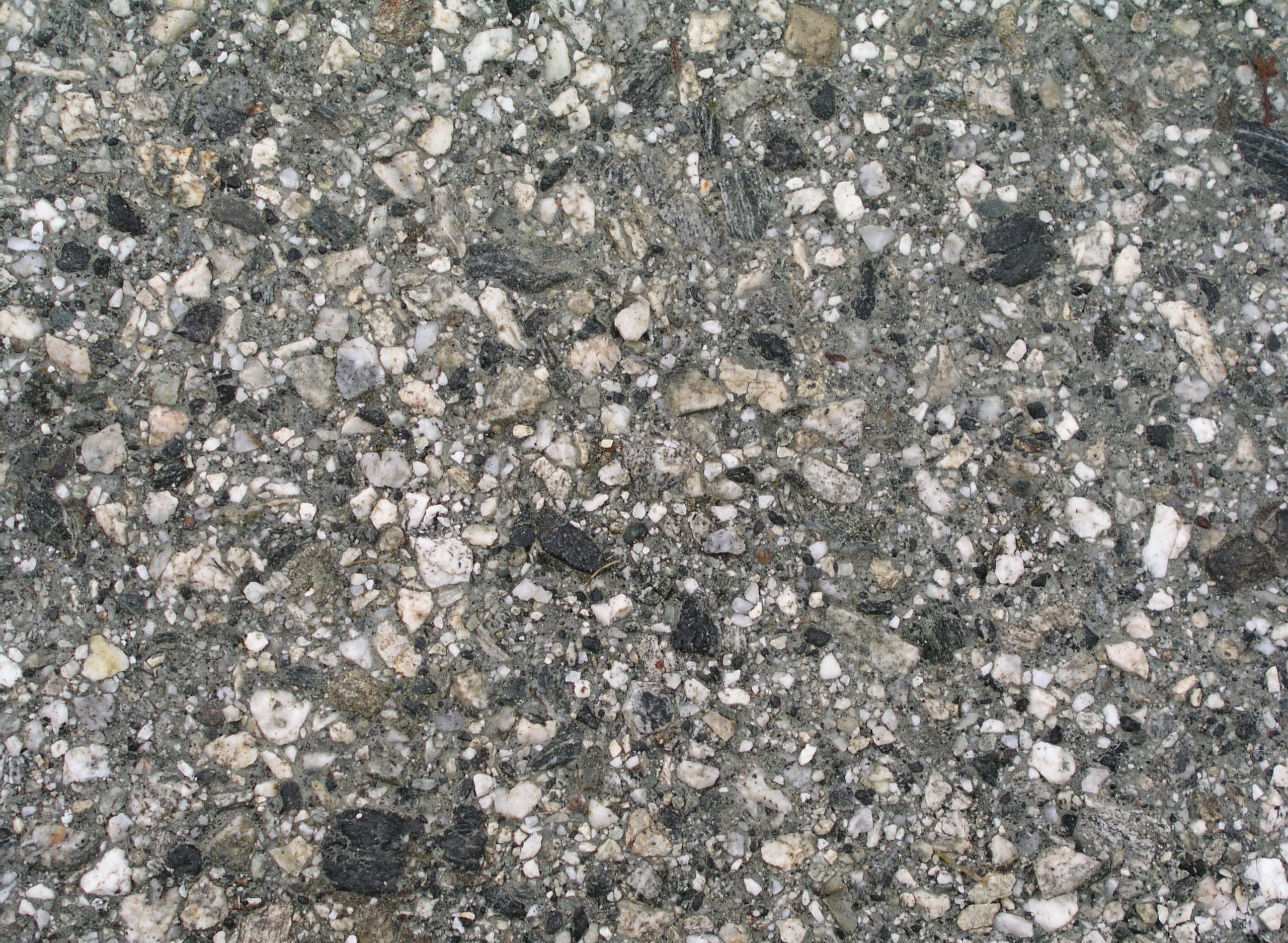

Supplement: Supplementary file 3 — Source Data [file 41467_2021_22856_MOESM3_ESM.zip › figure-7/python/texture/data/03_stony_earth.JPG]

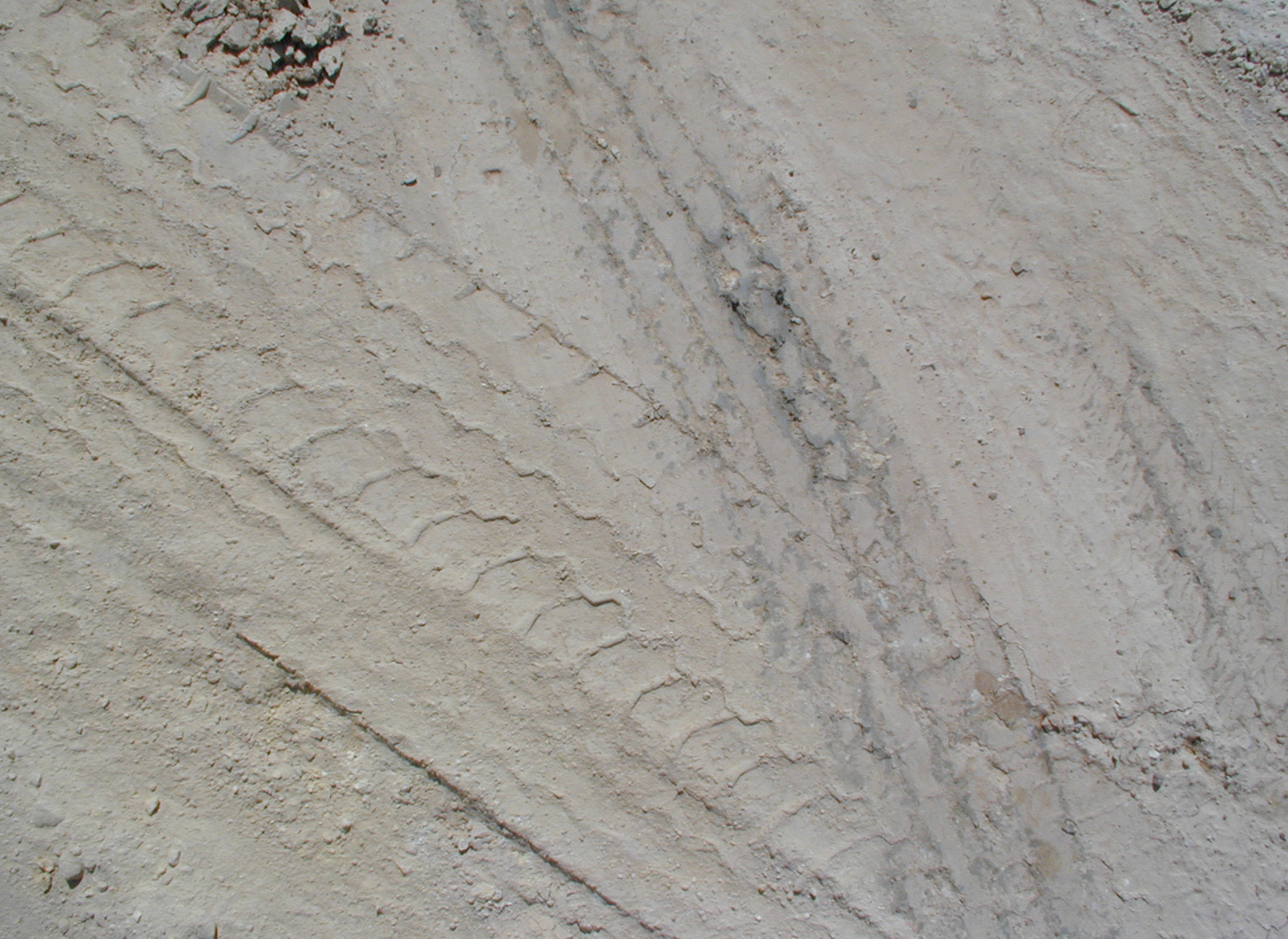

Supplement: Supplementary file 3 — Source Data [file 41467_2021_22856_MOESM3_ESM.zip › figure-7/python/texture/data/07_tyre_marks.JPG]

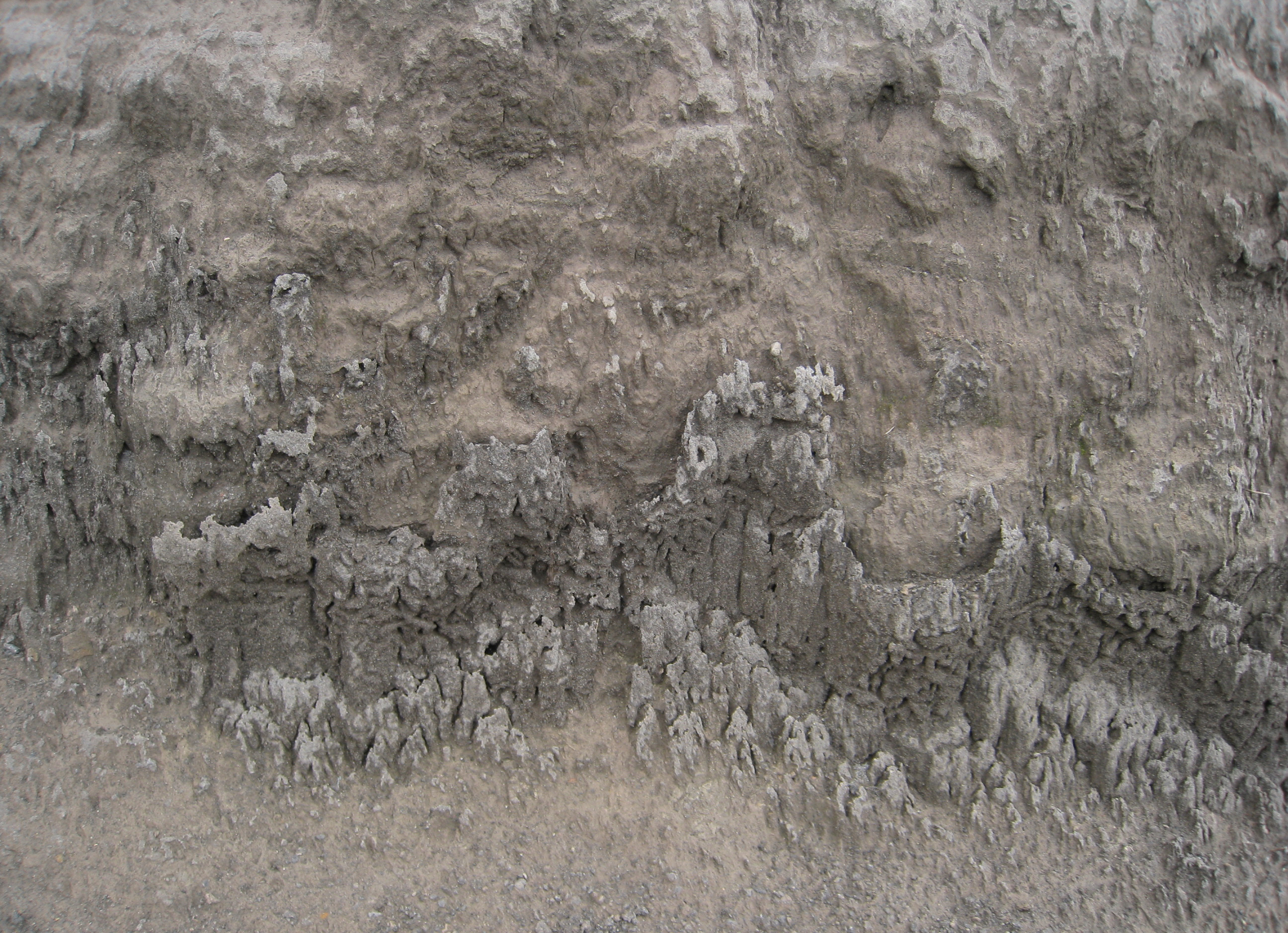

Supplement: Supplementary file 3 — Source Data [file 41467_2021_22856_MOESM3_ESM.zip › figure-7/python/texture/data/06_eroded_earth.JPG]

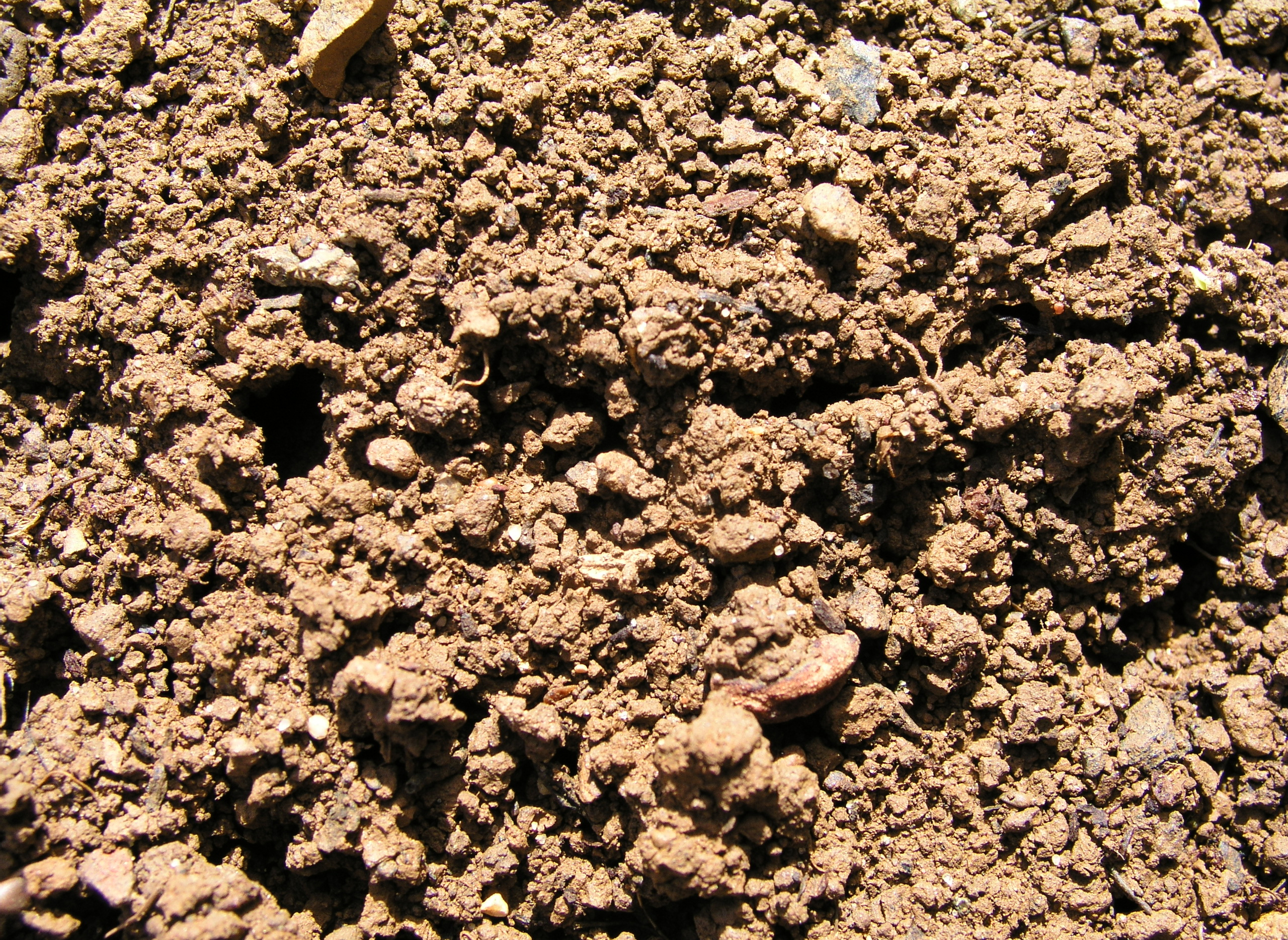

Supplement: Supplementary file 3 — Source Data [file 41467_2021_22856_MOESM3_ESM.zip › figure-7/python/texture/data/02_dried_earth_clots.JPG]

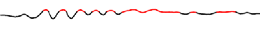

Supplement: Supplementary file 3 — Source Data [file 41467_2021_22856_MOESM3_ESM.zip › figure-7/python/memory/data/2012 Clack, Svoboda, Myers, PLoS Comput Biol, Figure 7, Trace G10.png]

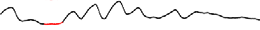

Supplement: Supplementary file 3 — Source Data [file 41467_2021_22856_MOESM3_ESM.zip › figure-7/python/memory/data/2012 Clack, Svoboda, Myers, PLoS Comput Biol, Figure 7, Trace G5.png]
